# Effects of aquaculture practices on *Vibrio* population dynamics and oyster microbiome

**DOI:** 10.1128/aem.01985-25

**Published:** 2025-12-15

**Authors:** Esam Almuhaideb, Nur A. Hasan, Christopher Grim, Shah Manzur Rashed, Salina Parveen

**Affiliations:** 1 Department of Agriculture, Food and Resource Sciences, University of Maryland Eastern Shore 14705, Princess Anne, Maryland, USA; 2 Center for Bioinformatics and Computational Biology, University of Maryland 271850 , College Park, Maryland, USA; 3 EzBiome Inc., Gaithersburg, Maryland, USA; 4 Human Foods Program, U.S. Food and Drug Administration, College Park, Maryland, USA; 5 Cosmos ID, Germantown, Maryland, USA; Anses, Maisons-Alfort Laboratory for Food Safety, Maisons-Alfort, France

**Keywords:** oyster microbiome, shotgun metagenomics, *Crassostrea virginica*, *Vibrio *spp., aquaculture practices

## Abstract

Oyster aquaculture is essential for ensuring a sustainable food source. Despite stringent controls, cases of oyster-related illnesses linked to pathogenic *Vibrio parahaemolyticus* (*Vp*) and *Vibrio vulnificus* (*Vv*) persist. This study investigated the impact of aquaculture practices on the oyster microbiome and pathogen levels, focusing on two common systems: on-bottom and floating cages. From June to November 2019, monthly samples were collected from the Chesapeake Bay, including oysters and water from each aquaculture system. Oyster samples included both fresh and temperature-abused oysters. The study utilized the most probable number and real-time PCR (MPN-qPCR) method to quantify total and pathogenic *Vp* and *Vv* in water and oyster samples. DNA was extracted from oyster homogenates and filtered water samples for shotgun metagenomic sequencing. The results revealed significant impacts of aquaculture practices on the diversity of the oyster microbiome, particularly affecting the distribution of phages, antibiotic resistance, and virulence factor genes. Shotgun metagenomic sequencing consistently showed higher genetic representation of *Vibrio* in floating cages for both fresh and temperature-abused oyster samples. MPN-qPCR results differed between practices, showing higher *Vibrio* levels in bottom cages for fresh oysters and higher levels in floating cages under temperature abuse. These discrepancies are likely explained by the stable conditions in bottom cages, the effects of temperature abuse, and the growth bias inherent to the MPN method. These results underscore the need for a holistic, time-sensitive approach, taking into account microbial states and the dynamic aspects of the oyster environment to understand the complex relationship between aquaculture practices and the oyster microbiome.

**IMPORTANCE:**

This study holds great importance for food safety, antibiotic resistance surveillance, aquaculture management, and environmental health. Unraveling the population dynamics of microbial communities in oysters and their responses to different aquaculture practices enhances our ability to ensure safer seafood, monitor antibiotic resistance, optimize aquaculture methods, and mitigate potential public health challenges. Moreover, it demonstrates the applicability of advanced metagenomic tools for future research. Furthermore, this research addresses critical aspects of food safety, food security, public health, and sustainable aquaculture practices, making it highly relevant in today’s context.

## INTRODUCTION

The consumption of raw and undercooked oysters, particularly those harvested from warm coastal waters, can result in *Vibrio* infections (1–4). Among the various *Vibrio* species, *Vibrio parahaemolyticu*s and *Vibrio vulnificus* stand out as significant pathogens responsible for causing vibriosis in humans (1–6). These bacteria thrive in marine environments and can proliferate to elevated levels in oysters, especially during the warmer months (1–6). Despite the implementation of *Vibrio* Control Plans mandated by the National Shellfish Sanitation Program, which involve quality and safety measures like best management practices and hazard analysis and critical control points, oyster-related illnesses caused by pathogenic *Vibrio* continue to rise (6–8). The complexity of *Vibrio* dynamics in oysters suggests that many factors influence its prevalence. This calls for a closer look at how various elements, such as aquaculture practices, might further influence the microbial communities in oysters (3, 9).

Oyster aquaculture is a key marine industry in the Chesapeake Bay, offering a sustainable and reliable food source (10). In this region, various growing systems are utilized, including the popular bottom cages equipped with 6-inch legs that elevate them above the seafloor (11). However, there is a noticeable shift in the industry toward incorporating a floating cage system. A floating cage is attached to air-filled pontoons, allowing it to float in the subsurface of the water (12). These floating cages offer numerous advantages, such as promoting optimal oyster growth, enhancing shell shape and appearance, and ensuring consistent production rates (12). Additionally, the floating cages can be periodically flipped, facilitating effective control of fouling (12). This transition to floating cages signifies a remarkable development in oyster farming practices in the Chesapeake Bay. While these advancements improve oyster farming efficiency, aquaculture practices can also influence the microbial communities associated with oysters, including *Vibrio* species.

Previous studies in North Carolina, Louisiana, and Massachusetts showed that the levels of *V. parahaemolyticus* and *V. vulnificus* in oysters can significantly differ between bottom and floating cages (13–15). However, research on the effects of aquaculture practices on *Vibrio* remains limited (13–16). Moreover, most studies have relied on culture-dependent methods, which primarily detect culturable microorganisms and may not fully capture the complexities of the oyster microbiome (4, 5, 13–16). Culture-independent techniques like next-generation sequencing, particularly metagenomic shotgun sequencing, enable the direct sequencing of nucleic acids from microbial communities in their natural habitats (17, 18). This approach provides valuable insights into the composition, diversity, and response of the oyster microbiome to various factors. However, since shotgun sequencing provides relative abundance estimates based on sequencing read counts, it does not measure actual bacterial concentrations, particularly *Vibrio* species. To overcome this limitation, the most probable number and real-time PCR (MPN-qPCR) technique was used alongside shotgun metagenomics. MPN-qPCR combines enrichment culture with qPCR detection, allowing quantification of target gene markers of *V. parahaemolyticus* and *V. vulnificus* and providing actual abundance measurements (19).

In addition to aquaculture practices, it is important to investigate post-harvest conditions, recognizing the potential impact of improper handling on *Vibrio* levels and the oyster microbiome (20–25). High levels of *V. parahaemolyticus* were observed in shellstock oysters obtained from restaurants in Gainesville, FL, indicating a potential occurrence of temperature abuse during handling or storage (21, 23). Additionally, when oysters are stored at ambient temperatures, *V. vulnificus* may increase up to 100-fold within 10 hours (22). An increase in the levels of *Vibrionaceae* in oysters during commercial transport and storage at various temperatures was also observed as the temperature increased (20). Furthermore, different storage temperatures have been found to impact the oyster microbiome (24, 25). Assessing the impact of aquaculture practices on *Vibrio* and the oyster microbiome at both harvest and post-harvest stages, alongside environmental factors, using MPN-qPCR and shotgun metagenomic methods, provides a comprehensive understanding of *Vibrio* dynamics and the oyster microbiome, laying a strong foundation for risk assessment.

**Fig 1:**
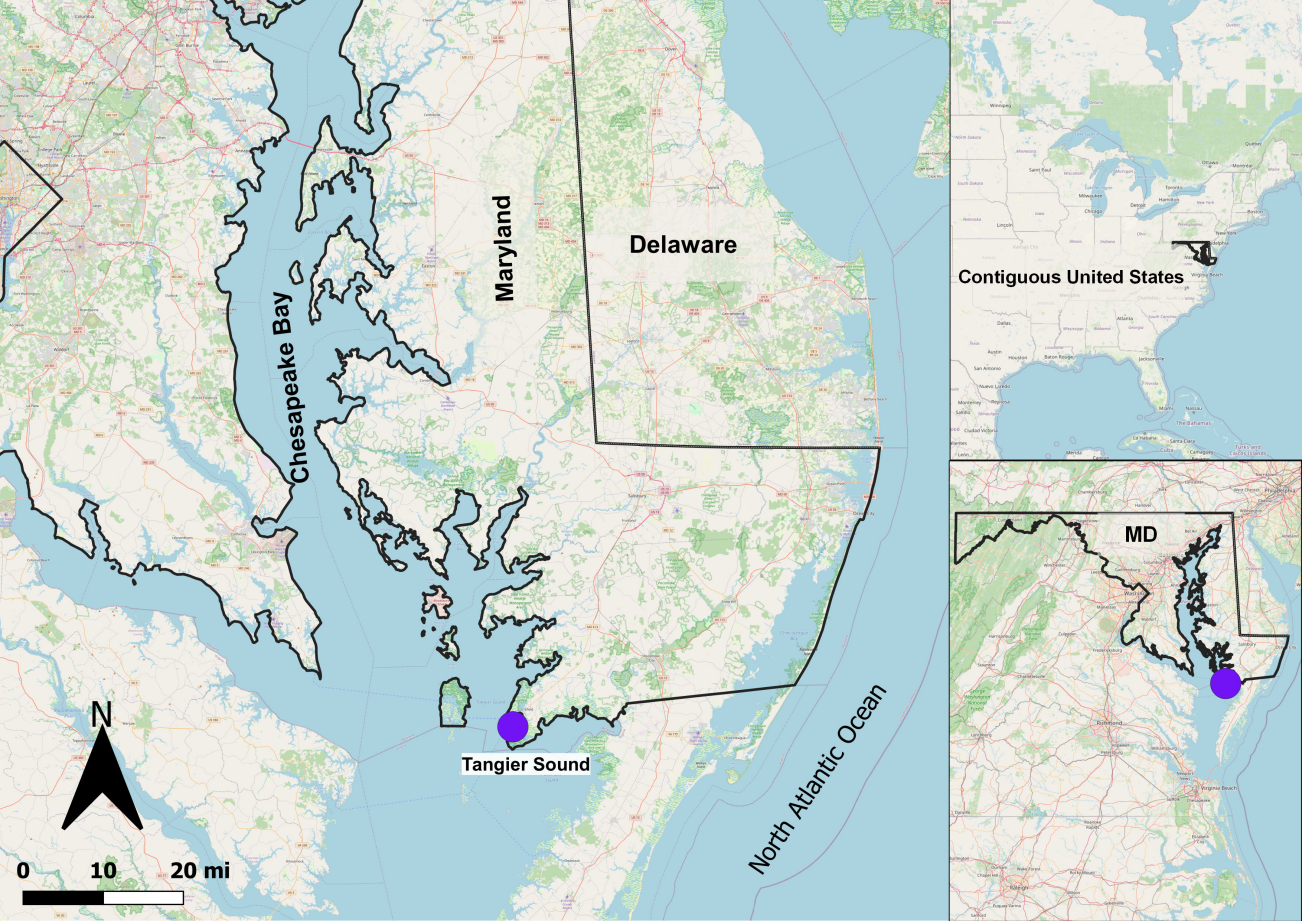
Map of the Chesapeake Bay sampling site. Map created in QGIS 3.14.15 using OpenStreetMap and U.S. Census TIGER/Line data.

The aim of this study was to investigate the effects of two aquaculture practices, namely bottom and floating cages, on the dynamics of the eastern oyster (*Crassostrea virginica*) microbiome collected from the Chesapeake Bay (Fig. 1). The study utilized the homogenate of fresh oysters, as well as temperature-abused oysters (Table 1), aiming to comprehensively grasp how aquaculture practices influence *Vibrio* population dynamics, as well as those of the oyster microbiomes, and if there is a differential effect on both fresh and temperature-abused oysters.

**TABLE 1:** Number and type of oyster^*a*^ and water samples

Sample type	No. of OY/W samples^*c*^	Sampling events	Total
Fresh oyster homogenate from bottom cages (FOHB)	3	6	18
Fresh oyster homogenate from floating cages (FOHF)	3	6	18
Temperature-abused oyster homogenate from bottom cages (AOHB)^*b*^	3	6	18
Temperature-abused oyster homogenate from floating cages (AOHF)^*b*^	3	6	18
Water from the bottom cages area (WBA)	1	6	6
Water from the floating cages area (WFA)	1	6	6

^*a*^ Oysters were processed individually.

^*b*^ Oysters assigned for temperature abuse were placed at 25°C for 24 h before processing.

^*c*^ OY, oysters; W, water.

## RESULTS

### DNA yield of the oyster samples

Before DNA extraction, we performed saponin treatment to eliminate host DNA. DNA concentration analysis revealed a broader and higher range for bottom oysters compared to floating oysters in both fresh and abused samples. Specifically, DNA concentrations ranged from 0.4 to 28.7 ng/µL in fresh oyster homogenate from bottom cage (FOHB) samples, 0.5 to 17 ng/µL in fresh oyster homogenate from floating cage (FOHF) samples, 0.3 to 32.9 ng/µL in temperature-abused oyster homogenate from bottom cage (AOHB) samples, and 0.3 to 18.3 ng/µL in temperature-abused oyster homogenate from floating cage (AOHF) samples (Fig. S1A). In comparison, water samples exhibited higher levels and greater variability in DNA concentration, ranging from 64.7 to 235.6 ng/µL in water from the bottom cages area (WBA) samples and from 67.1 to 248.1 ng/µL in water from the floating cages area (WFA) samples (Fig. S1B). Bottom-caged oysters yielded higher total and average DNA concentrations compared to their floating counterparts (Table 2).

**TABLE 2:** Average and total DNA concentration, sequencing raw reads, and hits reads^*a*^

Variable/sample type		FOHB	FOHF	AOHB	AOHF	WBA	WFA
DNA yield (ng/μL)	Avg.	4.5	2.5	3.2	2.9	148.9	179.2
	Sum	80	44	58	52	894	1,075
Raw reads	Avg.	55,872,301	74,473,964	57,975,454	58,054,661	18,403,150	19,759,083
	Sum	670,467,609	893,687,564	1,043,558,184	1,044,983,902	110,418,902	118,554,496
Bacterial hits	Avg.	2,179,866	3,401,371	396,585	418,315	790,500	827,500
	Sum	26,158,388	40,816,446	7,138,535	7,529,672	4,743,000	4,965,000
Phages hits	Avg.	41,624	89,490	1,045,734	299,529	37,880	46,605
	Sum	499,488	1,073,876	18,823,208	5,391,528	227,280	279,627
Viruses hits	Avg.	13,136	13,003	19,165	54,514	52,494	35,572
	Sum	157,628	156,032	344,961	981,249	314,961	213,430
Protists hits	Avg.	32,777	29,024	429	667	38,630	28,666
	Sum	393,327	348,286	7,728	12,007	231,780	171,996
Fungi hits	Avg.	16,682	116,505	3,594	43,246	15,444	12,171
	Sum	200,187	1,398,059	64,700	778,427	92,663	73,023
ARGs hits	Avg.	173	141	239	6,714	87	105
	Sum	2,080	1,690	4,296	120,843	519	627
VFGs hits	Avg.	784	1,079	1,774	474	528	544
	Sum	9,410	12,953	31,925	8,532	3,170	3,261
All biomarker hits	Avg.	2,285,042	3,650,612	1,467,520	823,459	935,562	951,161
	Sum	27,420,508	43,807,342	26,415,353	14,822,258	5,613,373	5,706,964
Microbial hits	Avg.	2,284,085	3,649,392	1,465,507	816,271	934,947	950,513
	Sum	27,409,018	43,792,699	26,379,132	14,692,883	5,609,684	5,703,076

^*a*^ Avg., average (the mean value calculated across replicates); Sum, obtained by adding the values from all replicates; ARGs, antibiotic resistant genes; VFGs, virulence factor genes; FOHB, fresh oyster homogenate from bottom cages; FOHF, fresh oyster homogenate from floating cages; AOHB, temperature-abused oyster homogenate from bottom cages; AOHF, temperature-abused oyster homogenate from floating cages; WBA, water from the bottom cages area; WFA, water from the floating cages area.

### Community diversity of the oyster microbiome

The average and total count of the raw and hits reads obtained from FOHB, FOHF, AOHB, AOHF, WBA, and WFA samples are displayed in Table 2. Additionally, the number of biomarkers identified and their abundance scores are shown in Table 3. In both fresh and temperature-abused oyster samples, we observed a higher number of identified bacterial species in oysters from floating cages compared to those from bottom cages (Table 3). Notably, this difference in bacterial species identification was not primarily driven by the total number of reads. The discrepancy in the percentage of bacterial hits relative to the total reads was minimal across all sample types, with differences in ratios not exceeding 0.6% (Table 2). For example, even though the total read counts varied between FOHF and FOHB samples, the percentage of identified bacterial reads was similar, 4.5% for FOHF and 3.9% for FOHB, suggesting that the higher bacterial diversity observed in floating cage oysters may be influenced by factors other than the starting read count, potentially including the conditions associated with their growing system. Additionally, the bacterial species identified in the WFA samples (645) exceeded that of the WBA samples (566) (Table 3). The abundance score of antimicrobial-resistant genes (ARGs) in the AOHF was also approximately 43 times higher than in AOHB (Table 3). This notable disparity in abundance can be primarily attributed to the significantly elevated presence of the tetracycline resistance gene *tet*C, which exhibited substantially higher levels in the AOHF oysters.

**TABLE 3:** Number of biomarkers identified and their abundance score among sample types^*a*^

Sample type	Bacteria	Phages	Viruses	Protists	Fungi	ARGs	VFGs
FOHB	288 (979,393)	96 (297,765)	25 (11,796)	29 (267)	14 (322)	8 (12,781)	26 (80,830)
FOHF	364 (1,457,644)	86 (495,870)	11 (2,338)	22 (259)	23 (1,084)	2 (1,418)	36 (60,789)
AOHB	222 (107,129)	79 (129,062,876)	40 (260,967)	3 (581)	7 (35)	8 (427,890)	23 (4,991,870)
AOHF	267 (230,581)	109 (29,231,207)	56 (430,925)	5 (1,160)	7 (26)	17 (18,704,857)	44 (1,200,469)
WAB	566 (320,008)	112 (204,731)	45 (4,908)	60 (1,068)	72 (309)	30 (36,053)	47 (69,402)
WAF	645 (351,500)	104 (236,609)	39 (3,887)	59 (1,019)	66 (264)	39 (67,069)	49 (99,537)

^*a*^ Number in parentheses indicates the abundance score; ARGs, antibiotic resistant genes; VFGs, virulence factor genes; FOHB, fresh oyster homogenate from bottom cages; FOHF, fresh oyster homogenate from floating cages; AOHB, temperature-abused oyster homogenate from bottom cages; AOHF, temperature-abused oyster homogenate from floating cages; WBA, water from the bottom cages area; WFA, water from the floating cages area.

### Abundance and diversity of bacterial taxa

**Fig 2:**
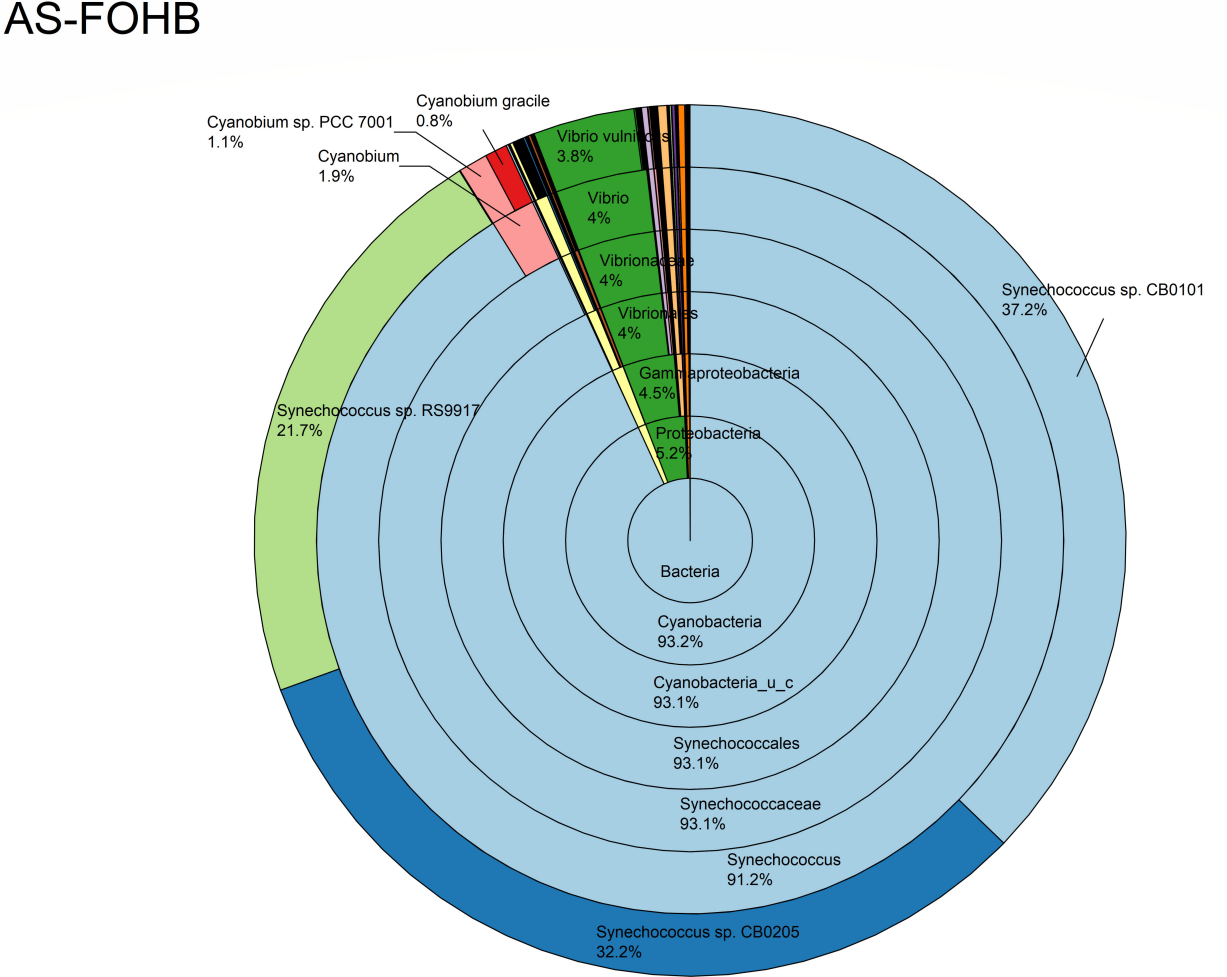
Relative abundance distribution of bacterial taxa per sample type. AS abundance score.

**Fig 3:**
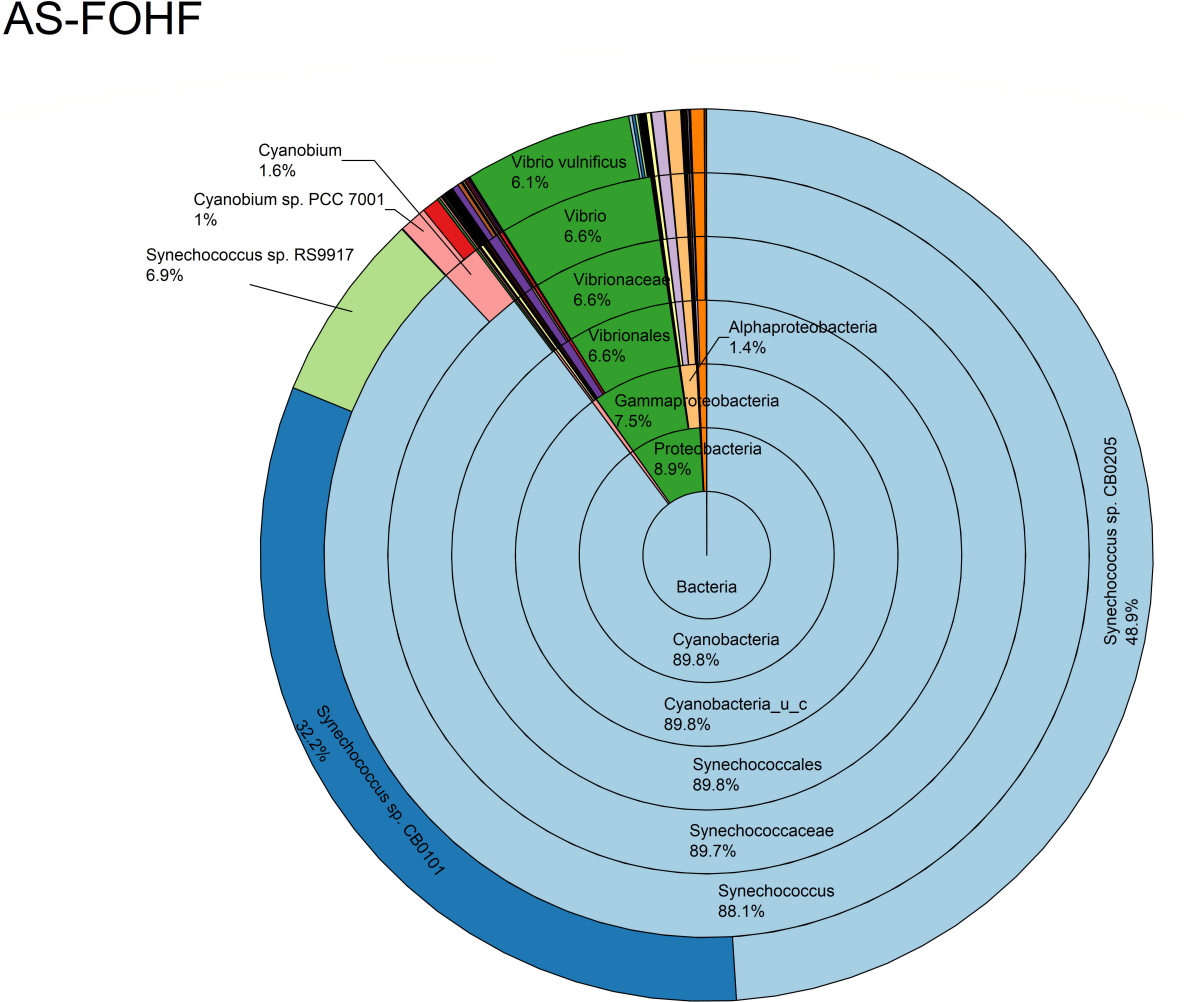
Relative abundance distribution of bacterial taxa per sample type.

**Fig 4:**
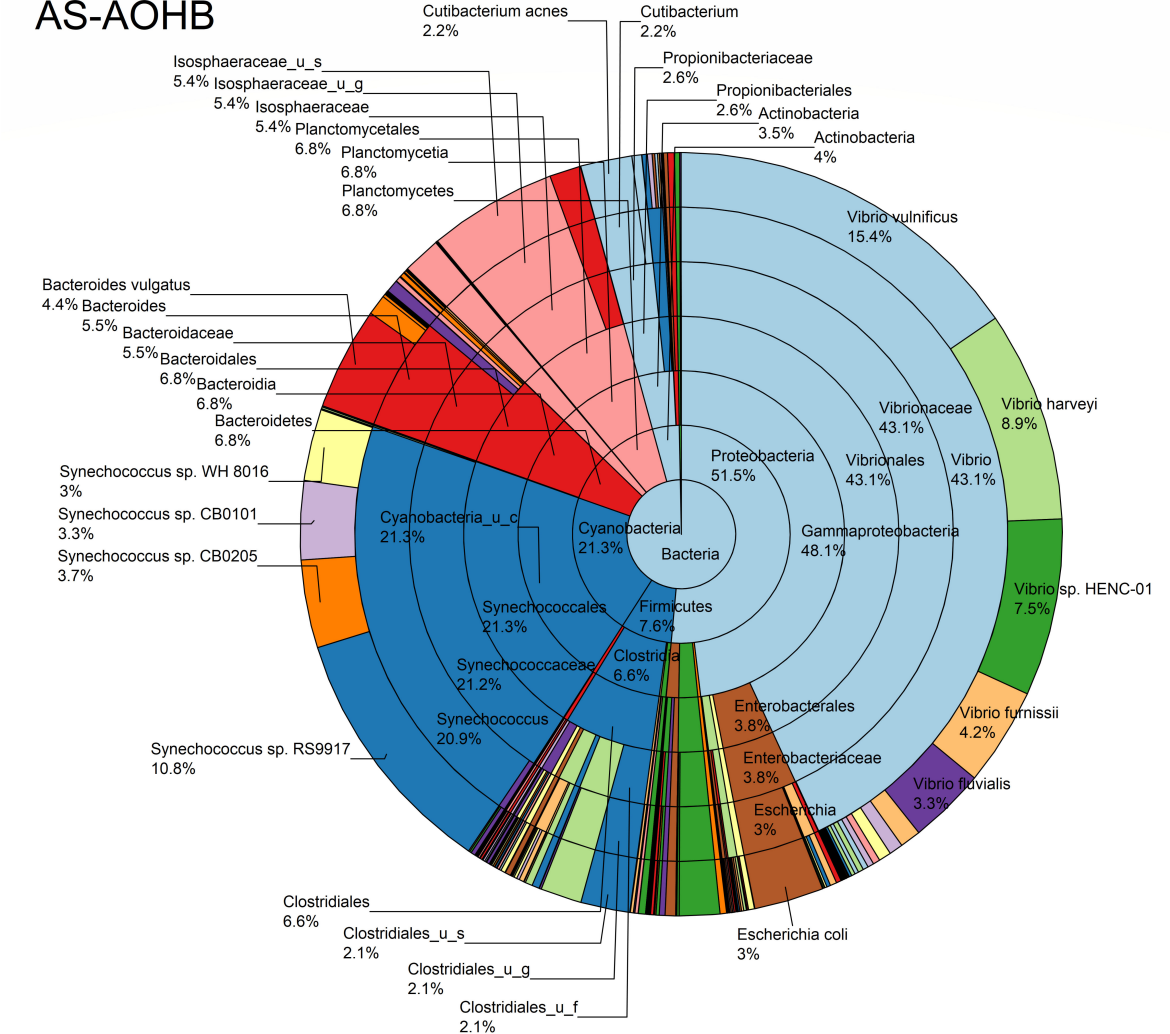
Relative abundance distribution of bacterial taxa per sample type.

**Fig 5:**
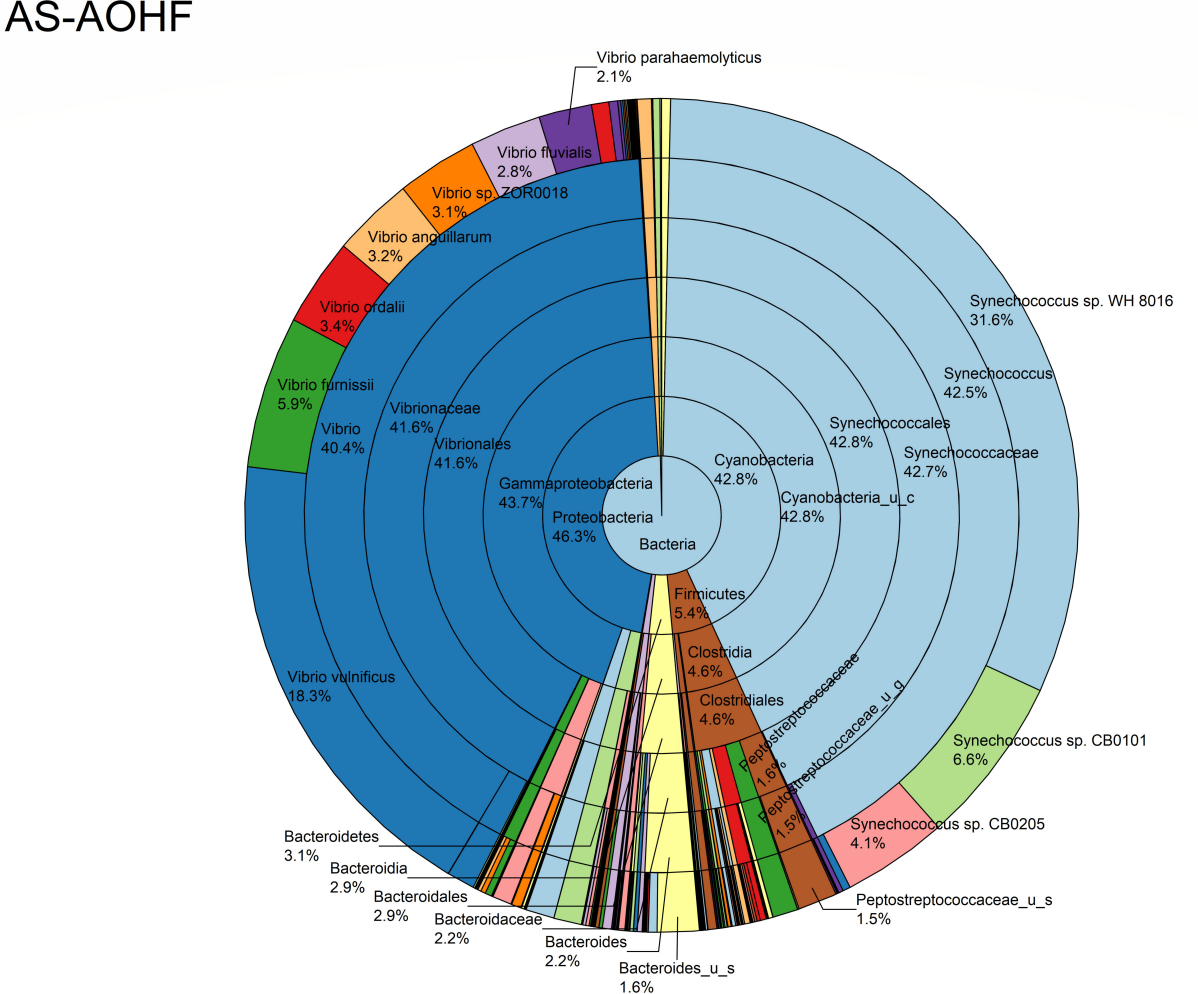
Relative abundance distribution of bacterial taxa per sample type.

The relative abundance analysis highlighted the dominance of *Synechococcus* sp. CB0205, sp. CB0101, and sp. RS9917 in both FOHB and FOHF samples (Fig. 2 and 3). *Synechococcus* sp. CB0205 and sp. CB0101 had a higher presence in FOHF compared to FOHB, while *Synechococcus* sp. RS9917 was more prevalent in FOHB than in FOHF (Fig. 2 and 3). In the AOHB and AOHF samples, the dominant species included *Synechococcus* sp. WH 8016, sp. RS9917, and *V. vulnificus*, with *V. vulnificus* notably more prevalent in AOHF samples (Fig. 4 and 5). In the water samples, *Synechococcus* sp. CB0205 and sp. CB0101 were identified as the dominant species (Fig. S2 and S3).

**Fig 6:**
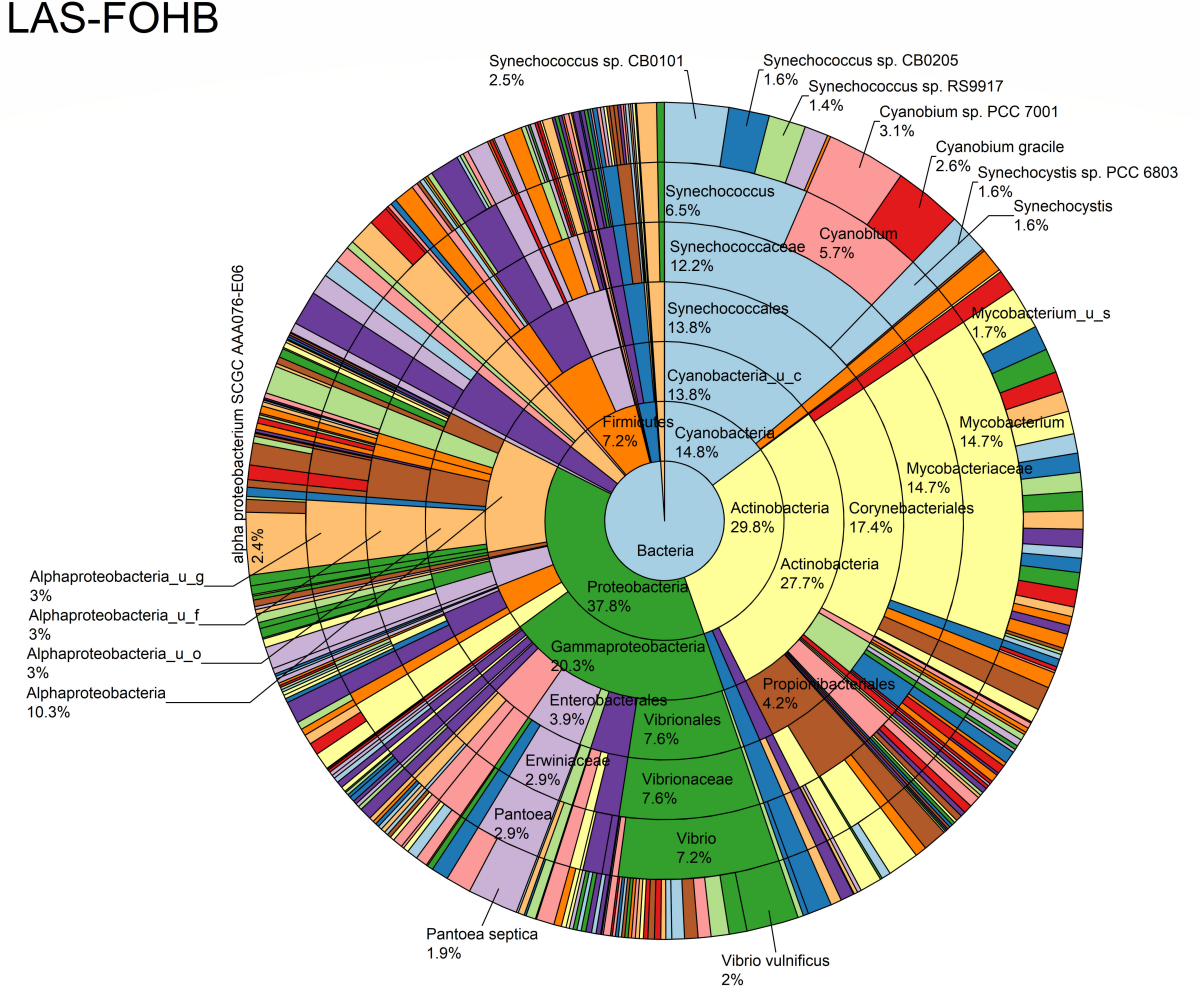
Log relative abundance distribution of bacterial taxa per sample type. LAS, log abundance score.

**Fig 7:**
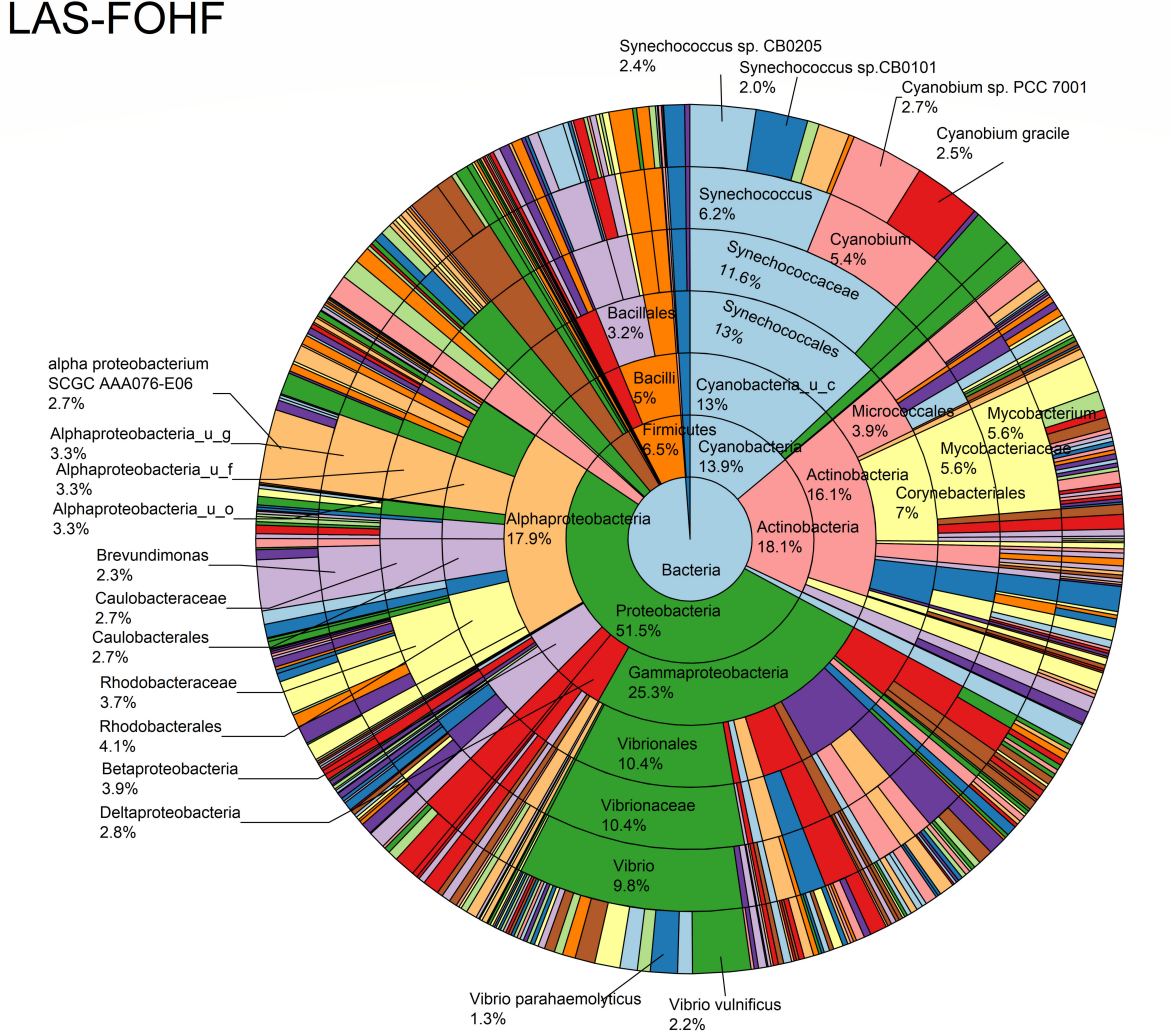
Log relative abundance distribution of bacterial taxa per sample type.

**Fig 8:**
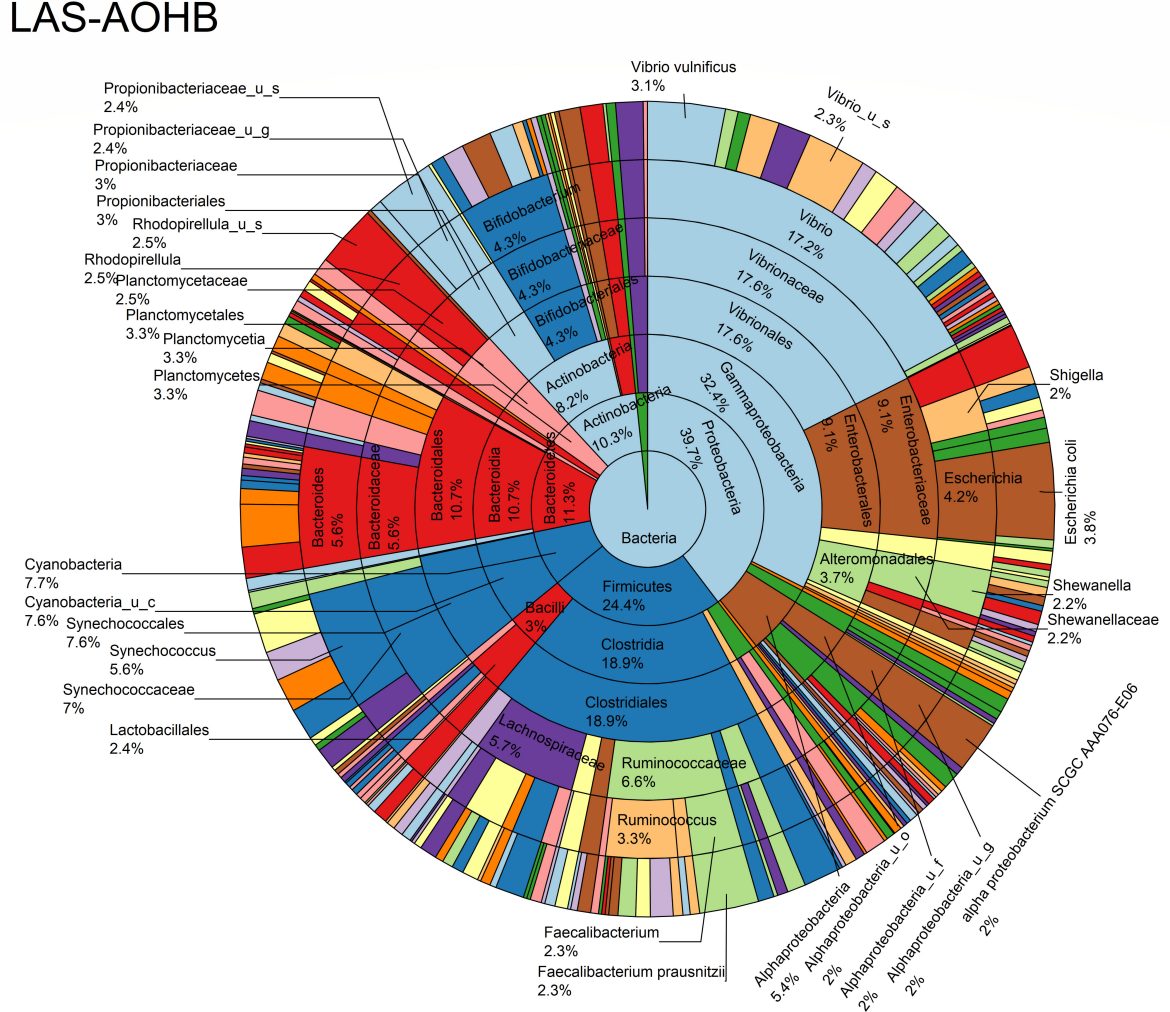
Log relative abundance distribution of bacterial taxa per sample type.

**Fig 9:**
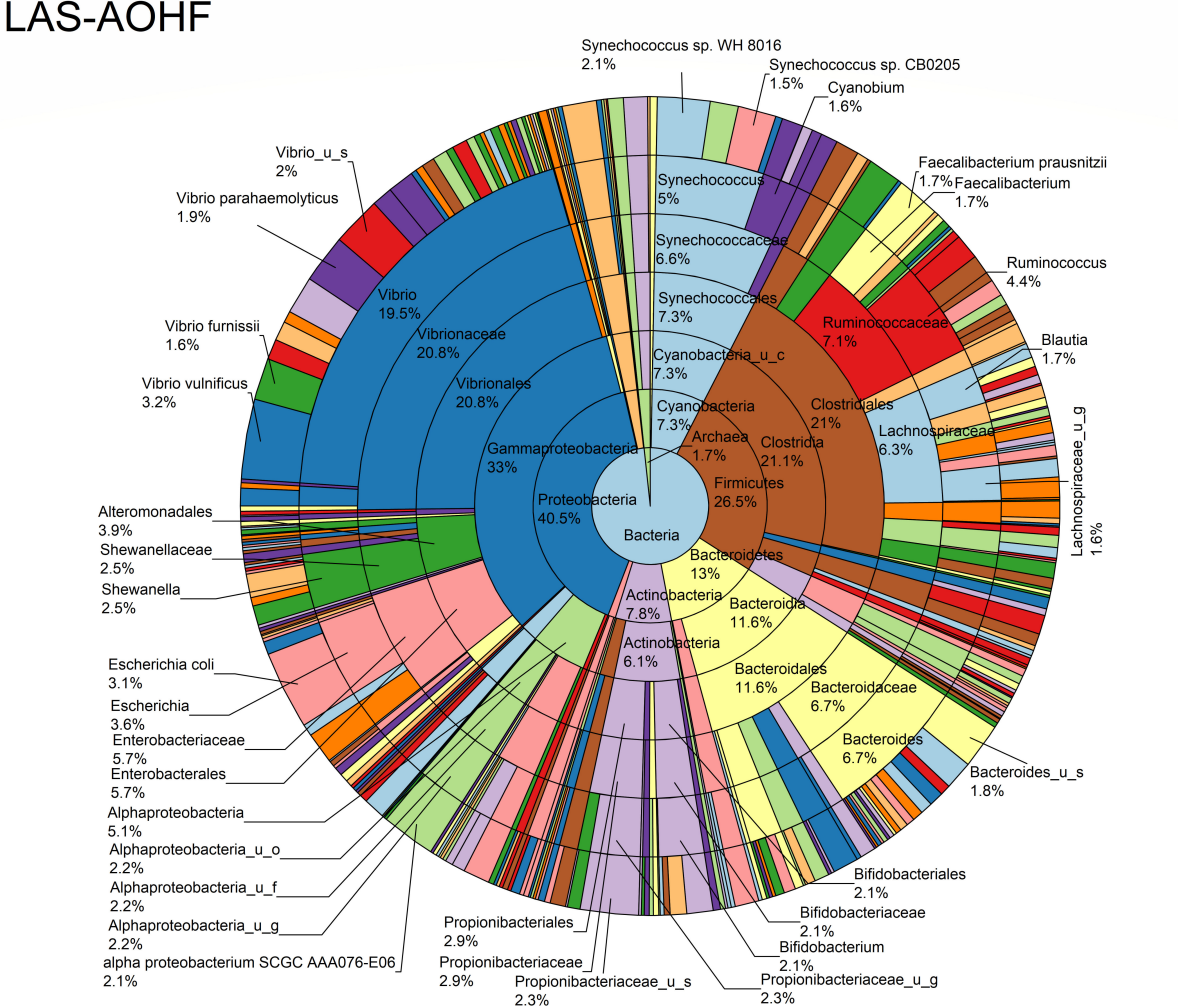
Log relative abundance distribution of bacterial taxa per sample type.

**Fig 10:**
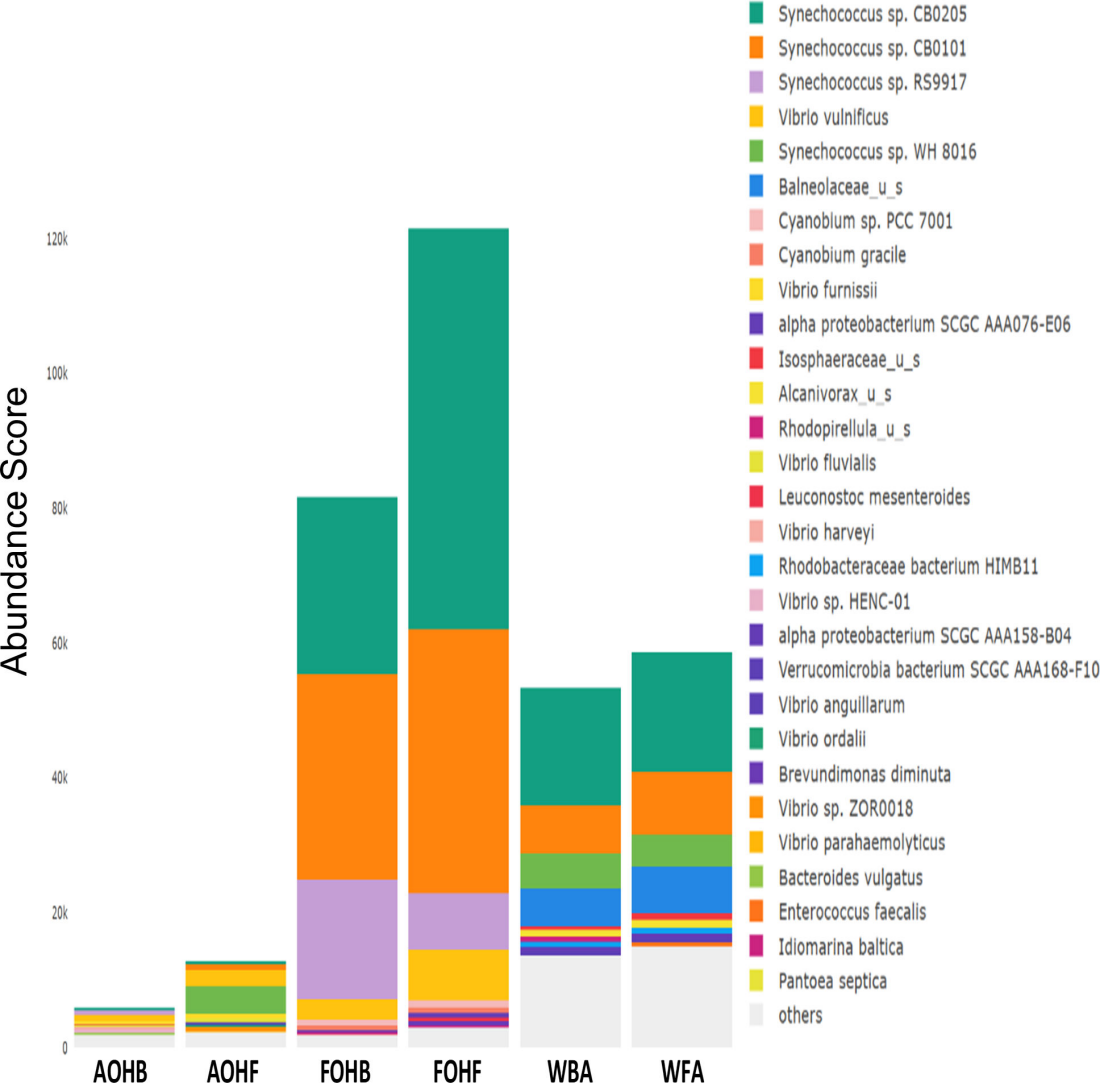
Relative abundance of bacterial species in relation to sample type.

**Fig 11:**
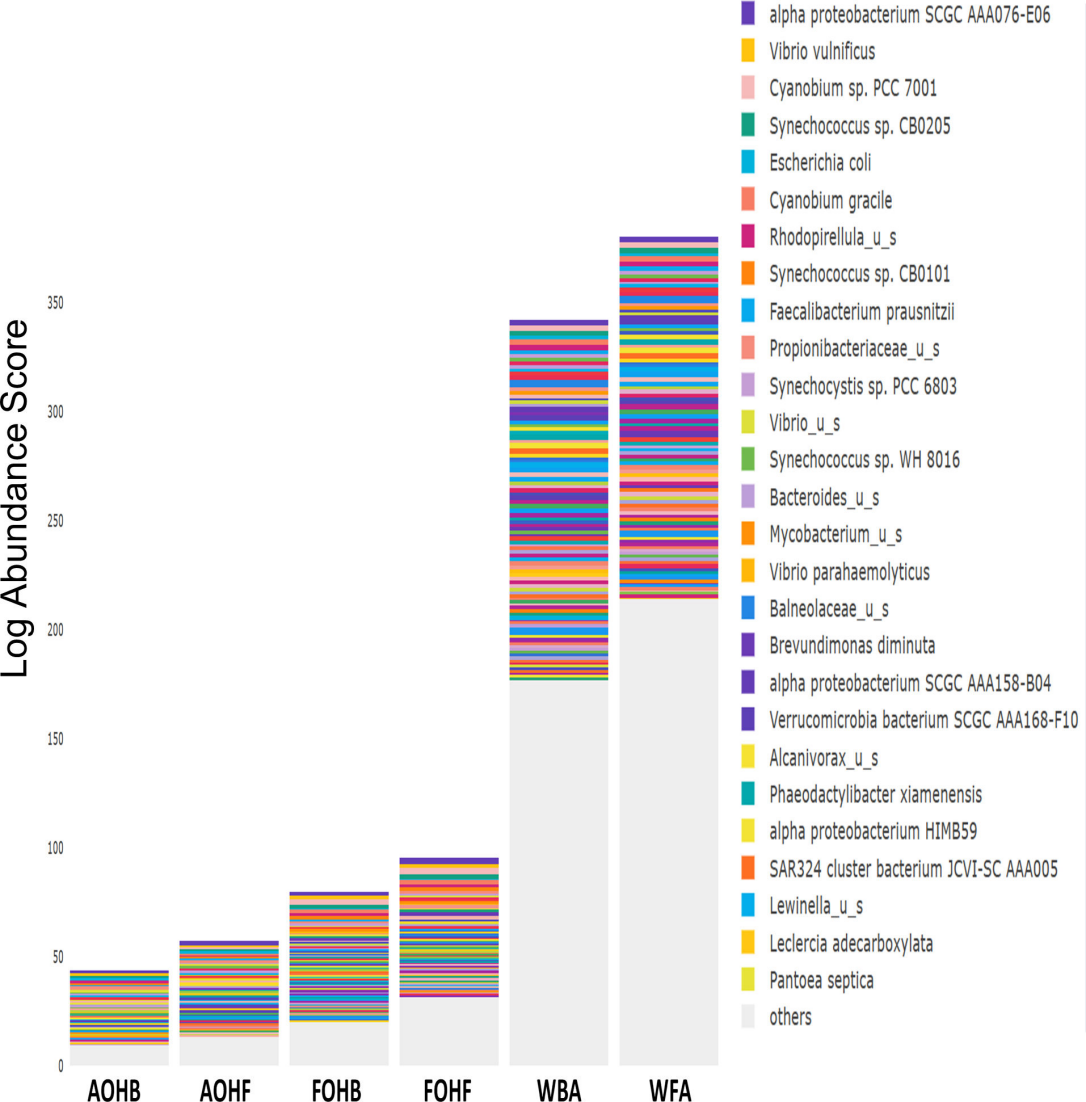
Log relative abundance of bacterial species in relation to sample type.

The analysis of log relative abundance in this study unveiled distinct dominant species within each oyster group. In FOHB, *Cyanobium* sp. PCC 7001 accounted for 3.1% of the abundance, while alpha proteobacterium SCGC AAA076-E06 was dominant in FOHF at 2.7% (Fig. 6 and 7). In contrast, *Escherichia coli* dominated AOHB at 3.8%, and *V. vulnificus* (3.2%) was prevalent in AOHF (Fig. 8 and 9). Notably, several *Vibrio* species, particularly *V. vulnificus* and *V. parahaemolyticus,* displayed higher log relative abundance in the floating cages (Fig. 6 to 9; Data Set S1). *V. vulnificus* consistently exhibited a higher log relative abundance than *V. parahaemolyticus* in all oyster groups (Data Set S1). Furthermore, the prevalence of *Vibrio* spp. in water samples was significantly lower compared to oyster samples (Fig. S4 and S5). This finding highlights that oysters can bioaccumulate *Vibrio* spp. even when the *Vibrio* levels in the oyster environment are low (26). It is important to note analysis of the raw abundance data (untransformed sequencing read counts) indicated that *Synechococcus* was the dominant taxon in both oyster and water samples, except in AOHB, primarily due to extremely high counts in a subset of replicates that skewed the mean value (Fig. 10). To mitigate the influence of these outliers and better visualize the patterns among consistently abundant taxa, we applied a log-transformation of the abundance scores. This revealed a different community structure, where taxa with consistent presence across replicates, such as *Cyanobium* sp. PCC 7001 and *C. gracile* in fresh oysters, and *E. coli* and *V. vulnificus* in temperature-abused oysters, emerged as the dominant groups (Fig. 11).

Statistical analysis of the abundance distribution indicated that the species, such as *Cyanobium* sp. PCC 7001, alpha proteobacterium SCGC AAA076-E06, *E. coli*, *V. vulnificus*, *V. parahaemolyticus*, and *Synechococcus* sp. CB0205, were not significantly different between practices (*P* > 0.05) (Fig. S6 to S11). However, temperature abuse, which represents post-harvest mishandling, can influence the effects of the aquaculture practices on bacterial species differently (Fig. S6 to S11). For example, in fresh oysters, the prevalence of *Cyanobium* sp. PCC 7001, *V. vulnificus*, and *Synechococcus* sp. CB0205 was notably different between practices, but such distinctions were less apparent in abused oysters, indicating that other factors may introduce bias and mask the effects of aquaculture practices on the prevalence of bacterial species in oysters (Fig. S6 to S11).

**Fig 12:**
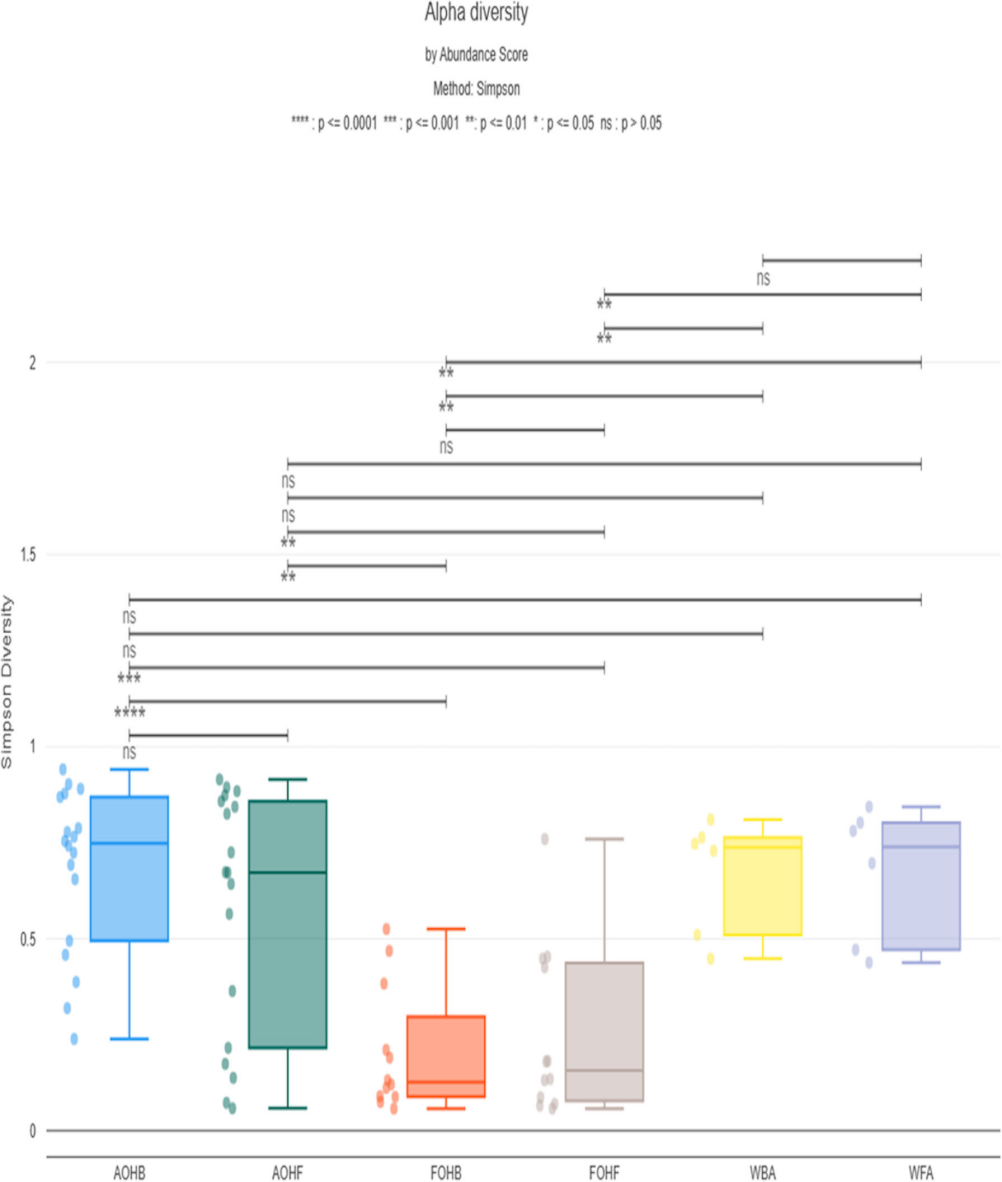
Simpson index representing the richness and evenness of bacterial species within all sample types. Each dot represents the Simpson diversity value for an individual sample. Statistical comparisons of medians across sample types were performed using a Wilcoxon rank-sum test.

The alpha diversity, as measured using the Simpson index, produced median values of 0.057 in the FOHB to 0.94 in the AOHB (Fig. 12). Bacterial richness and evenness were not significantly different between practices among both fresh and temperature-abused oysters as indicated by the Wilcoxon Rank Sum test (*P* > 0.05). Beta diversity between practices was measured using Bray-Curtis, and the permutational multivariate analysis of variance (PERMANOVA) test of the Bray-Curtis values indicated that microbial composition and their relative abundance were not significantly different between practices in both fresh and temperature-abused oysters (*P* > 0.05). Alpha and beta diversity of the surface water from the bottom and floating cages areas were not significantly different.

### Taxonomic composition analysis of bacteria between practices

**Fig 13:**
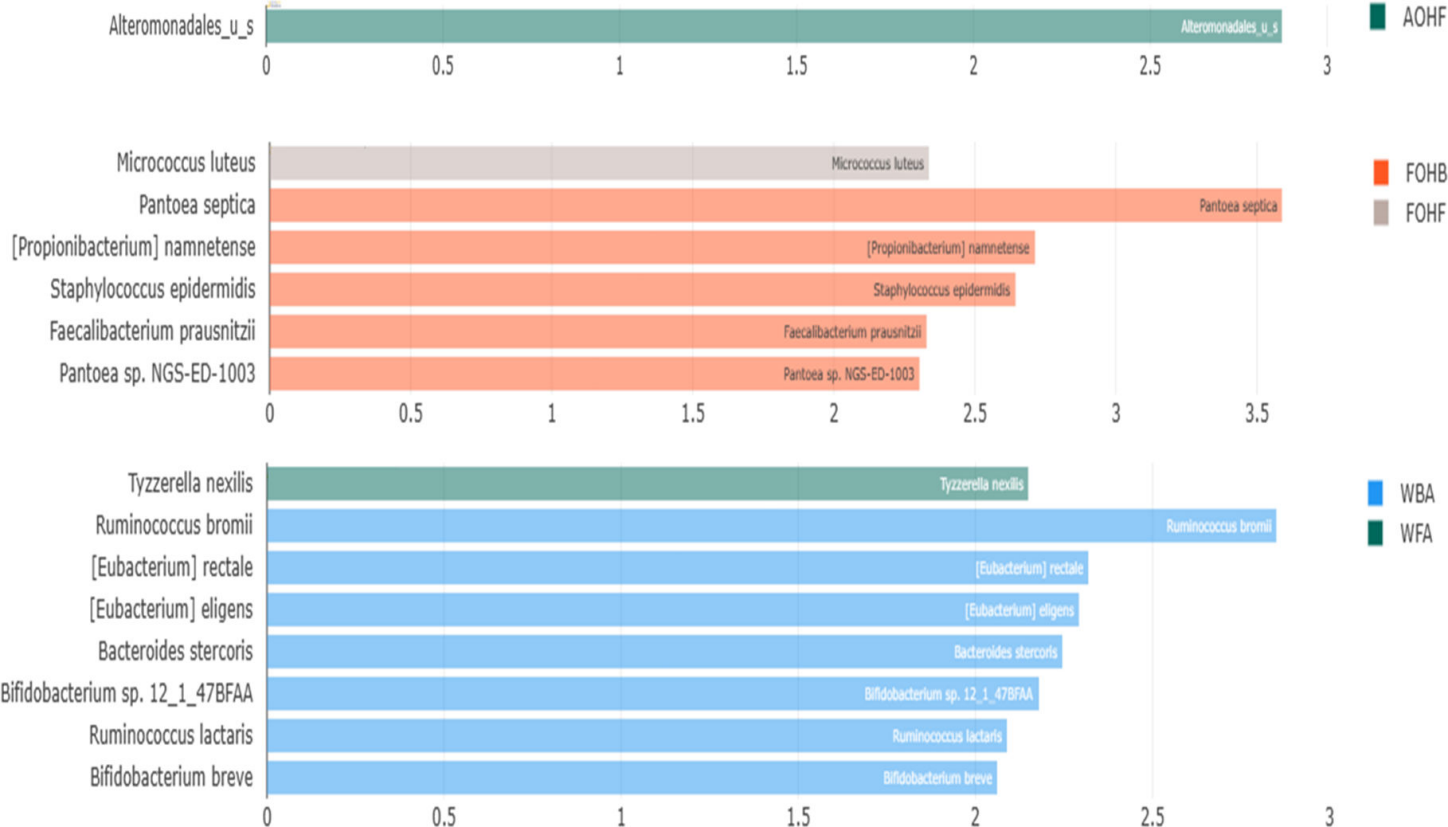
Distinctive bacterial species in relation to sample type.

In contrast to the overall community diversity indices, significant differences in the oyster microbiome were observed between sample types, using the linear discriminant analysis (LDA) effect size (LEfSe) analysis. Fifteen differential bacterial taxa (LEfSe: LDA score > 2) were identified to be taxonomic signatures of a particular sample type (Fig. 13). For example, at the bacterial species level, *Alteromonadales* sp. in the AOHF; *Micrococcus luteus* in the FOHF; *Pantoea septica*, *Propionibacterium namnetense*, *Staphylococcus epidermidis*, *Faecalibacterium prausnitzii*, and *Pantoea* sp. NGS-ED-1003 in the FOHB; *Tyzzerella nexilis* in the WFA; *Ruminococcus bromii*, *Eubacterium rectale*, *Eubacterium eligens*, *Bacteroides stercoris*, *Bifidobacterium* sp. 12_1_47BFAA, *Ruminococcus lactaris*, and *Bifidobacterium breve* in the WBA were the distinctive bacterial species (Fig. 13).

### Abundance and diversity of DNA viruses, fungi, and protists

This study focused on DNA viruses and phages, as the nucleic acid extraction and sequencing methods targeted DNA. Oyster samples displayed a limited presence of identified viruses, protists, and fungi, whereas the abundance of identified phages was comparatively higher (Table 3). The virus that had the highest relative abundance within the FOHB and FOHF samples was *Chrysochromulina ericina* virus (13% and 35%), while the marine snail-associated circular virus was the dominant virus in the AOHB (8%) and AOHF (9%) samples, respectively (Fig. S12). Furthermore, aquaculture practices did not cause notable changes in the dominant viruses in AOHB/AOHF and FOHB/FOHF, as similar viruses consistently ranked among the top ten dominant species in both fresh and temperature-abused oysters (Fig. S12 and S13). The water samples displayed a similar trend, with the majority of the top ten dominant viruses being shared between WBA and WFA samples (Fig. S12 and S13). *Escherichia* virus HK630, *Cyanophage* KBS-S-2A, and *Synechococcus* phage S-CBS1 were the predominant phages identified in AOHB (10%)/AOHF (9%), FOHB (4%)/FOHF (3%), and WBA (3%)/WFA (3%), respectively (Fig. S14). In contrast to the diversity seen in viruses, the majority of the phages dominating both oyster and water samples belonged to the *Synechococcus* species (Fig. S15), which is consistent with the observed dominance of *Synechococcus* bacteria in our samples.

The protists and fungi, as measured by read abundance, were notably lower in all sample types compared to other microbial targets, as expected (Table 3). *Salpingoeca rosetta* and *Chromera velia* were the dominant protists in FOHB, WBA, WFA, and FOHF samples, respectively, while *Entamoeba hartmanni* dominated the AOHB and AOHF samples (Fig. S16). Among fungi identified, *Agaricomycetes* sp. dominated the AOHF, FOHF, WBA, and WFA samples, while *Malassezia restricta* and *Fungi* sp. were the dominant species in the AOHB and FOHB samples, respectively (Fig. S17).

Simpson analysis (alpha diversity) was conducted, and the resultant values were used for the statistical analysis using the Wilcoxon test. The findings revealed that, except for phages in fresh oysters, where alpha richness and evenness exhibited a significant difference (*P* < 0.05) between FOHB and FOHF samples (Fig. S18), aquaculture practices and water samples from each system did not yield significant effects on the richness and evenness of phages in AOHB/AOHF and WBA/WFA samples, nor did they significantly impact viruses in any sample type. Statistical analysis for the Simpson values of protists and fungi could not be calculated due to their low abundance. Regarding beta diversity, Bray-Curtis analysis was performed, and the resulting values were used for the PERMANOVA test, which indicated that only the composition and relative abundance of fungi in the FOHB/FOHF samples exhibited significant differences (Fig. S19).

### Taxonomic composition analysis of DNA viruses, protists, and fungi between practices

In the temperature-abused oysters and water samples, there were no discriminative viruses, protists, and fungi between practices based on LEfSe analysis. On the other hand, a total of 23 differential phage taxa (LEfSe: LDA score > 2) were identified as the taxonomic signatures of the fresh oyster samples, with *Staphylococcus* phage StB20-like and *Synechococcus* phage S-RSM4 emerging as the most significantly distinctive phages of the FOHB and FOHF samples, respectively (Fig. S20). LEfSe analysis did not reveal large populations to be significantly discriminative regarding non-phage viruses, protists, and fungi in the fresh oyster samples.

### Abundance and diversity of antimicrobial resistance and virulence factor genes

A total of 10 unique ARGs were identified in the fresh oyster, 23 in the temperature-abused oyster, and 49 in the seawater samples (Data Set S1; Fig. S21). In contrast to the fresh oyster samples, the temperature-abused samples from the floating cages (AOHF) had a higher overall abundance score of ARGs compared to oysters from the bottom (AOHB) (Table 3). Tetracycline *tet*C was the most predominant ARG in both AOHB (51%) and AOHF (39%). However, despite its lower relative proportion in AOHF, its absolute log abundance score was substantially higher, more than 14 logs greater than *tet*C in AOHB (Data Set S1). Other dominant ARGs included aminoglycoside aadD (26%) in FOHB, multidrug-resistant (MDR)-efflux-pump mexJ (50%) in FOHF, MDR-efflux-pump mdtP (8%) in WBA, and regulator cpxR (5%) in WFA (Fig. S21). Furthermore, tetracycline *tetC* appeared more frequently in the AOHF replicates (Fig. S22). Three MDR-efflux-pump genes were exclusively identified in floating cage oysters (AOHF and FOHF) (Data Set S1). In contrast, a greater number of MDR-efflux-pump genes were detected in the water samples, where they were also more prevalent (Data Set S1).

Virulence factor genes (VFGs) were identified across all sample types, with some unique to specific sample types, while others were shared across multiple samples, resulting in 23 in AOHB, 44 in AOHF, 24 in FOHB, 36 in FOHF, 47 in WBA, and 49 in WFA samples (Data Set S1). These VFGs were associated with 22 bacterial species, and a majority of these genes (104) were linked with *Bacteroides fragilis*, *Francisella tularensis*, *Enterobacter aerogenes*, *Bacteroides thetaiotaomicron*, *E. coli*, and *Vibrio cholerae* (Data Set S1). The total abundance score of the VFGs in AOHB and FOHB was higher than in AOHF and FOHF (Table 3). The dominant VFGs included *E. coli* GENE *iss* in the AOHB (35%) and AOHF (27%), *Staphylococcus lentus* GENE *repL* in the FOHB (20%), *E. aerogenes* GENE *tniA* in the FOHF (5%), *Klebsiella pneumoniae tnp* in the WBA (5%), and *Enterobacter* GENE *hylA* in the WFA (6%) samples (Fig. S23). *E. coli iss* was primarily detected in the AOHB/AOHF samples (Fig. S24).

The Simpson analysis of the alpha diversity in the ARGs and VFGs produced values that were not significantly different between the growing systems. Bray-Curtis analysis of beta diversity indicated that the composition and relative abundance of the ARGs and VFGs in the fresh oysters were significantly distinct between bottom and floating oysters (*P* value < 0.05) (Fig. S25 and S26).

### Prevalence of *V. parahaemolyticus* and *V. vulnificus*

**Fig 14:**
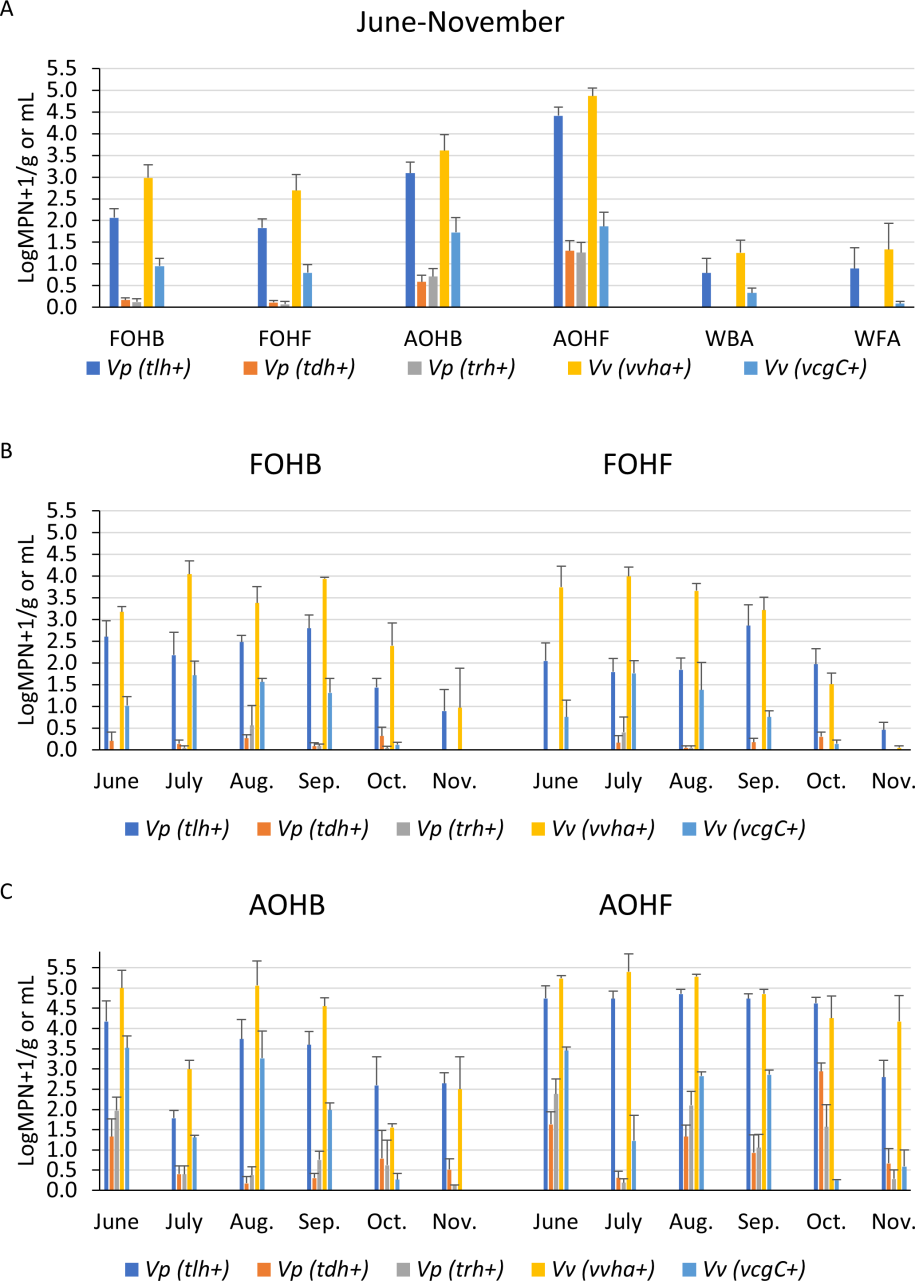
Averages of total and pathogenic *V. parahaemolyticus* (*Vp*) and *V. vulnificus* (*Vv*) in relation to sample type, practices, and months. *tlh,* thermolabile hemolysin; *tdh,* thermostable hemolysin; *trh,* thermostable-related hemolysin; *vvh*A, *V. vulnificus* hemolysin A gene; *vcg*C, virulence correlated gene-clinical genotype. (**A**) *Vibrio* levels across sample types and practices. (**B**) Monthly *Vibrio* levels in fresh oysters from bottom and floating cages. (**C**) Monthly *Vibrio* levels in temperature-abused oysters from bottom and floating cages.

Oysters and water samples from the bottom and floating systems were assessed for the presence of total and pathogenic *V. parahaemolyticus* (*tlh* and *tdh*/*trh* genes) and *V. vulnificus* (*vvhA* and *vcgC* genes), using the quantitative MPN-qPCR method. In general, results indicated that individual oysters, even from the same practice and month, can differ up to 2.8 LogMPN/g higher in abundance of total *V. parahaemolyticus* and *V. vulnificus* (Data Set S1). The average LogMPN/g indicated that total and pathogenic *V. parahaemolyticus* and *V. vulnificus* were more prevalent in the FOHB than in FOHF samples (Table 4; Fig. 14A). In contrast, total and pathogenic *V. parahaemolyticus* and *V. vulnificus* were more prevalent in the AOHF than in the AOHB samples (Table 4; Fig. 14A). On a monthly basis, no specific trends of the abundance of total and pathogenic *V. parahaemolyticus* and *V. vulnificus* were observed between FOHB and FOHF samples (Fig. 14B). However, total and pathogenic *V. parahaemolyticus* and total *V. vulnificus* tend to be more prevalent in the AOHF than in the AOHB (Fig. 14C). Pathogenic *V. vulnificus*, however, tends to be more prevalent in the AOHB than in the AOHF (Fig. 14C). Yet, the LogMPN level of total and pathogenic *V. parahaemolyticus* and *V. vulnificus* in oysters was not significantly different between practices (*P* > 0.05).

**TABLE 4:** Comparison of levels of *V. parahaemolyticus* (*Vp*) and *V. vulnificus* (*Vv*)^*a*^

Sample type	Min–Max Avg. (LogMPN/g or mL)				
	*Vp* (*tlh*+)	*Vp* (*tdh*+)	*Vp* (*trh*+)	*Vv* (*vvh*A+)	*Vv* (*vcg*C+)
FOHB	0.283–3.38 2.064	ND–0.723 0.168	ND–1.471 0.122	ND–4.628 2.982	ND–2.314 0.953
FOHF	0.133–3.632 1.827	ND–0.484 0.114	ND–1.093 0.075	ND–4.628 2.694	ND–2.632 0.798
AOHB	1.398–5.171 3.089	ND–2.16 0.586	ND–2.632 0.709	1.215–5.66 3.612	ND–4.378 1.726
AOHF	2.382–5.366 4.415	ND–3.324 1.301	ND–2.868 1.264	3.168–6.279 4.865	ND–3.632 1.867
WBA	0.039–1.975 0.795	ND–0.019 0.006	ND–0.01 0.003	0.002–1.975 1.256	ND–0.681 0.332
WFA	0.016–3.168 0.894	ND–0.007 0.002	ND–0.004 0.001	0.002–3.632 1.332	ND–0.287 0.09

^*a*^ *tlh*, thermolabile hemolysin; *tdh*, thermostable hemolysin; *trh*, thermostable-related hemolysin; *vvh*A*, V. vulnificus* hemolysin A gene; *vcg*C, virulence correlated gene-clinical genotype; ND, not detected. FOHB, fresh oyster homogenate from bottom cages; FOHF, fresh oyster homogenate from floating cages; AOHB, temperature-abused oyster homogenate from bottom cages; AOHF, temperature-abused oyster homogenate from floating cages; WBA, water from the bottom cages area; WFA, water from the floating cages area.

## DISCUSSION

This is the most current study to date to examine microbiome profiles of the eastern oyster and the prevalence of the total and pathogenic *V. parahaemolyticus* and *V. vulnificus* using the shotgun metagenomic sequencing, along with the MPN-qPCR method on individual oysters from different aquaculture systems in the Chesapeake Bay. Compared to previously reported studies of the eastern oyster microbiome, this study included a larger number of oyster replicates and sampling time (27–30).

In our study, we applied saponin treatment with the aim of minimizing host DNA contamination and enriching microbial DNA for shotgun metagenomic sequencing. Saponin has been successfully used across various sample types, such as human and food samples (31–36). However, contrary to its expected outcome, the saponin treatment in our case did not significantly reduce the host DNA proportion, as evidenced by the ratio of raw reads to microbial hits (Table 2). This outcome suggests that saponin may have limitations in effectively minimizing host DNA in oyster tissues. Previous studies in other matrices have shown variable outcomes depending on tissue type, the specific chemical agents and concentrations used, and the commercial host DNA depletion kits applied (32, 37–40). Therefore, bivalve tissues may require protocol optimization to improve host DNA depletion efficiency. Despite this, the treatment did not negatively impact the detection of key bacterial genera commonly associated with the eastern oyster microbiome. Genera, such as *Synechococcus*, *Cyanobium*, *Vibrio*, *Photobacterium*, *Escherichia*, *Shewanella*, *Francisella*, *Mycobacterium*, and *Bacteroides*, which belong to the phyla Cyanobacteria, Proteobacteria, Actinobacteria, and Bacteroidetes, were still detected with relatively high abundance across various sample types. These findings align with those of previous studies (30, 41–47). Additionally, the Firmicutes phylum, though its constituent genera were not as prominent, was also relatively abundant in some samples, consistent with earlier research (42, 44, 45, 47). Conversely, certain classes and genera, such as Mollicutes (*Mycoplasmatota*), Chlamydiae (*Chlamydiota*), Spirochaetia (*Spirochaetota*), and Fusobacteriia (*Fusobacteriota*), along with the phylum Verrucomicrobia, which have been reported as dominant in other eastern oyster microbiome studies (30, 42–45, 47), were either absent or present at very low levels in our data. These differences may be due to biases introduced by sequencing methodologies, such as the choice of primers in 16S rRNA sequencing, which target different variable regions (e.g., V1–V2, V4–V5) (48–50). Such primer selections can influence which taxa are detected, and PCR amplification itself can introduce biases by preferentially amplifying certain taxa over others (48–50). By contrast, shotgun metagenomics does not target rRNA genes; rather, it sequences total DNA, thereby avoiding primer bias and providing an unbiased profile of the entire microbial community (17, 18). This approach enables high-confidence taxonomic assignment of the most abundant taxa present and can simultaneously identify bacteria, viruses, fungi, and protists (17, 18). It has also been reported that saponin treatment for host DNA depletion can, to some degree, alter microbial profiles by reducing the abundance of Gram-negative bacteria (51), which may further contribute to the observed differences. Although key oyster-associated bacterial genera were consistently detected in our study, the potential impact of saponin treatment on community structure should be further evaluated in future experiments.

The oyster microbiomes were analyzed at the species and strain levels, which provided more details about the microbial composition and their relative abundance, antimicrobial resistance genes, and the virulence factors associated with each microbial community. Shotgun sequencing analysis revealed differences in oyster microbiome diversity between the different aquaculture systems. These variations can be primarily linked to several factors, including original microbial load, physical stresses, and aquaculture practices.

The higher DNA concentrations in the bottom oysters suggest potential differences in microbial load between bottom and floating oysters. However, given the influence of various factors, including the saponin treatment, DNA concentration alone may not fully reflect microbial abundance. Shotgun metagenomic sequencing indicated that bacterial hits were the most abundant across all sample types, followed by phages.

Alpha diversity, measured using the Simpson index, did not significantly differ between bottom and floating cage oysters. However, a non-significant trend toward greater bacterial richness was consistently observed in floating cage oysters under both fresh and abused conditions. This trend aligns with the higher number of total bacterial hits identified in this study. This suggests a potential, though not statistically significant, influence of aquaculture practice on oyster microbiome diversity. Beta diversity analysis using Bray-Curtis showed no significant differences in microbial composition and relative abundance between practices in both fresh and abused oysters, which indicates that aquaculture practices may affect the alpha diversity of the oyster microbiome more than beta diversity. LEfSe analysis identified discriminative bacterial taxa between practices, with distinct species characterizing each group. Notably, significant differences in bacterial prevalence were observed between practices in the fresh oysters, but these distinctions were less apparent in abused oysters, confirming that the influence of aquaculture practices can be altered by other factors, which is in this context the temperature abuse treatment applied to oysters. Analysis of dominant bacterial species revealed the predominance of *Vibrio* spp., especially *V. vulnificus*, in floating cages, potentially suggesting a selective influence of aquaculture practices on the prevalence of specific bacterial species. It is worth highlighting that the overall abundance score of pathogenic *V. vulnificus* NBRC 15645 = ATCC 27562 appeared to be higher in the floating cage oysters compared to the bottom cage oysters under both fresh and abused conditions. However, as this trend was based on detection in a limited number of replicates (data not shown), it should be interpreted with caution and requires confirmation in future studies with a larger sample size. If confirmed, future work could explore potential contributing factors, such as wave dynamics, differences in filtration rates, interactions with phytoplankton, or other system-specific environmental variables, for possible explanations.

Oyster samples exhibited low identified viruses, protists, and fungi, with a higher abundance of phages. Although most of the dominant phages in the oysters and water samples belong to the *Synechococcus* genus, Simpson index analysis showed that significant differences in phage richness and evenness between FOHB and FOHF were observed at the species level. Within oyster samples, a set of dominant viruses identified were consistently among the top ten across different aquaculture practices. The influence of aquaculture practices on these dominant viruses appeared to be relatively minimal.

Our analysis revealed that the oyster viromes were largely dominated by viral families within the order Caudovirales, particularly Siphoviridae, Myoviridae, Autographiviridae, and Podoviridae (Data Set S1). In addition, we identified a significant representation of viral families outside Caudovirales, including Circoviridae and Virgaviridae, which were also prominent within the oyster viromes. These observations are in agreement with previous studies, highlighting the diverse viral composition in oysters and demonstrating the alignment of our results with established research on oyster viromes (52, 53).

Protists and fungi were present in lower abundance in oyster samples than other microbial types, which might be caused by factors such as their accumulation rates within oysters, DNA extraction biases, or the effect of the aquaculture practices. Notably, their reduced abundance in oyster samples aligns with their limited levels in the water samples, suggesting that aquaculture practices or other factors have no effect on their presence in oysters and primarily reflect their natural abundance in the environment. The abundance of ARGs was notably higher in AOHF compared to AOHB, while the abundance of VFGs was higher in AOHB than in AOHF. Alpha diversity analysis indicated no significant differences in ARGs and VFGs between growing systems, while beta diversity analysis revealed significant differences in the composition and relative abundance of these genes between FOHB and FOHF samples.

The findings of this study underscore the dynamic and fluctuating nature of the effect of aquaculture practices on the oyster microbiome. Just as the oyster microbiome changes over time, the effects of these practices may not manifest uniformly across all microbial components within the oyster community simultaneously. Importantly, these results raise an interesting prospect that microbial communities within the oyster microbiome may serve as indicators for one another. Drawing an analogy from our observations with phages, ARGs, and VFGs, the significant effects of aquaculture practices on these biomarkers, specifically in the fresh oysters, may signify a broader impact on the others, even if their diversity analyses did not reveal significance at a specific moment in time. This temporal variability stresses the need for a holistic, time-sensitive approach when evaluating influence on the oyster microbiome. By embracing the notion of fluctuation and change over time, we recognized that a snapshot of the microbiome at a single time point may not fully capture the broader influence of aquaculture practices. Future research should explore these temporal dynamics to uncover the intricate, continuously changing relationship between aquaculture practices and the oyster microbiome.

The results of this study indicate that individual oysters from the same niche can exhibit differences in the abundance of *V. parahaemolyticus* and *V. vulnificus*, highlighting the complexity of microbial dynamics within oyster populations. Similar trends were reported in previous studies on the abundance of these bacteria in individual oysters (54–57). These findings support and reinforce the notion that oyster-associated *Vibrio* populations can exhibit considerable heterogeneity even when oysters were collected from the same source during the same sampling time. Environmental conditions, oyster health, or even the inherent stochasticity of microbial communities might play roles in driving these differences.

Shotgun metagenomic sequencing showed a consistent pattern: both *V. parahaemolyticus* and *V. vulnificus* were more represented in floating cage oysters (FOHF and AOHF) than in bottom cage oysters (FOHB and AOHB). This indicates a greater presence of genetic material from both pathogens relative to the total microbial community, but not necessarily absolute pathogen levels. The MPN-qPCR results, however, revealed a different trend. In fresh oysters, higher MPN levels were found in bottom cages, while in temperature-abused oysters, higher levels were seen in floating cages. One possible explanation is that the stable bottom environment may support *Vibrio* populations that remain metabolically active, allowing them to proliferate more efficiently during laboratory enrichment and resulting in higher MPN estimates. This advantage, however, may not persist under temperature abuse. Post-harvest temperature abuse provides favorable conditions for *Vibrio* proliferation directly within oyster tissues, which may override pre-existing viability differences and shift the balance toward higher levels in floating-cage oysters (6). It is important to note that the nature of the MPN assay itself may also influence the variability observed. As an enrichment-based and dilution-to-extinction method, MPN favors cells capable of rapid outgrowth in enrichment media. Differences in strain composition, background microbiota, or even random distribution of cells among replicate tubes can amplify small initial differences and produce divergent results (58).

### Conclusion

This study offers a novel and insightful exploration of the intricate relationship between aquaculture practices and the oyster microbiome. As the first of its kind, to the best of our knowledge, it sheds light on the dynamic and fluctuating nature of microbial communities within oysters subjected to different aquaculture systems. This dynamic nature, influenced by aquaculture practices, doesn't manifest uniformly across all microbial components within the oyster community, indicating the potential use of specific biomarkers as indicators for the broader microbial community. These findings highlight the need for time-resolved sampling strategies to capture the dynamic nature of the oyster microbiome under varying aquaculture practices.

While trends in *Vibrio* abundance were observed between systems, no statistically significant differences in absolute *Vibrio* abundance were detected. However, the study highlights the importance of considering culturable populations when assessing aquaculture practice effects, as demonstrated by the discrepancies between metagenomic sequencing and MPN-qPCR data. These disparities raise intriguing questions about the sensitivity of processing methods and the potential role of enrichment processes in *Vibrio* proliferation in bottom-cage oysters. Ultimately, this research contributes to the expanding knowledge of aquaculture practices and their impact on oyster microbial communities, emphasizing the complex interactions at play and paving the way for more effective oyster farming methods.

## MATERIALS AND METHODS

### Study location and sampling

Eastern oysters (*Crassostrea virginica*) and water samples were collected from Tangier Sound (37°58′10.0″N 75°53′10.0″W) in the Chesapeake Bay (Fig. 1). Oysters were cultured in floating and bottom cages located within the same lease area, approximately 30 m apart, to ensure comparable environmental conditions. Bottom cages were positioned at a depth of ~1.7 m resting ~15 cm above the sediment, whereas floating subsurface cages were positioned ~0.3–0.5 m below the water surface. Oysters and water samples from both systems were collected using a boat to minimize disturbance of bottom sediments. The oysters used in both systems originated from the same hatchery stocks and genetic lines and were of market size (2–3 in) at the time of sampling. Physicochemical water parameters, including temperature, salinity, turbidity, dissolved oxygen, chlorophyll a, and pH, were recorded at the time of each sampling using a YSI EXO2 (Yellow Springs Instrument Co., Yellow Springs, OH) (Data Set S1). These measures were collected to ensure that environmental conditions were comparable between floating and bottom systems.

Samples were collected once a month from June to November 2019 from two different aquaculture systems, bottom and floating cages. Six oysters from each system were harvested and placed into plastic collection bags. Also, 1 L of water was collected from the overlying water of each system at the same time using sterile Nalgene bottles. After harvesting, oysters and water bottles were put into a cooler with ice using a sheet of bubble wrap to ensure no direct contact between the ice and oysters. A Smart Button Data Logger was used to confirm that the temperature during transportation is lower than 10°C (6).

### Processing of oyster and water samples

A total of 12 water and 72 oyster samples were used in this study (Table 1). Water samples were divided into two groups based on the growing systems area as follows: (i) water collected from the floating cages area (WFA), (ii) water collected from the bottom cages area (WBA) (Table 1). Oysters were processed individually and divided into four groups based on the harvest and post-harvest stages and the growing systems as follows: (i) fresh oyster homogenates from floating cages (FOHF), (ii) fresh oyster homogenates from bottom cages (FOHB), (iii) temperature-abused oysters from floating cages (AOHF), (iv) temperature-abused oysters from bottom cages (AOHB) (Table 1). Oysters assigned for temperature abuse were placed at 25°C for 24 hours before processing. This temperature was selected to simulate post-harvest handling under warm ambient conditions, as oysters close their shells once removed from the water and can support rapid *Vibrio* proliferation if not refrigerated (6). Oysters were cleaned using a scrub brush under the tap water before they were shucked with sterile knives. Tissue and liquor from each oyster were placed into a stomacher bag, buffered in 1:1 (wt/vol) phosphate buffer saline (PBS), and pressed using hand force to prepare the sample homogenate.

### Preparation of shotgun metagenomic samples

Aliquots of 1.5 mL from each homogenate were collected into 2 mL screw tubes, and 500 mL from each aquaculture-system water sample was filtered through a 0.2 µm Sterivex filter. Each oyster homogenate and the filters from each water sample were stored at −80°C until DNA extraction.

### Total DNA extraction, library preparation, and sequencing

Saponin treatment was performed prior to DNA extraction to reduce host DNA contamination, following the method described in our previous study (59). Briefly, 1 mL of each oyster homogenate was centrifuged at 2,000 × *g* for 30 seconds, followed by multiple wash and centrifugation steps to remove host DNA. After treatment with saponin and Turbo DNase, the resulting supernatant was used for DNA extraction. The saponin treatment selectively depletes eukaryotic host DNA by exploiting fundamental differences in cell membrane composition. Saponin specifically binds to cholesterol in eukaryotic membranes, creating pores that lead to osmotic lysis and the release of intracellular host DNA (31–36). The subsequent addition of Turbo DNase enzymatically degrades this exposed DNA. As prokaryotic cells lack cholesterol in their membranes, they remain protected from this lysis and DNA degradation. During the final DNA extraction process, only the intact genomic DNA is purified, while the degraded host DNA is removed during the washing steps, thereby enriching the sample for microbial DNA (31–36). DNeasy PowerSoil Kit and DNeasy PowerWater Kit (Qiagen, USA) were used to extract DNA from the oyster and filters of the water samples, respectively, following the manufacturer’s recommendation. DNA concentration was obtained using a Nano Drop (Thermo Fisher Scientific, USA). Preparation of sequencing libraries was performed using Nextera XT DNA Library Preparation Kit (Illumina Inc., USA) following the manufacturer’s protocol. DNA sequencing was performed by CosmosID using the Illumina HiSeq X (60, 61), following established protocols designed to capture microbial diversity and enable meaningful abundance score comparisons across sample types.

### Microbial detection and antibiotic resistance/virulence factor prediction

FASTQ files generated were analyzed by the CosmosID-HUB Microbiome bioinformatics software package (61). The system uses a data-mining k-mer algorithm that quickly separates millions of short DNA sequence reads into the specific genomes that produced them. The system has two phases: a pre-computation phase that uses curated databases of reference genomes, virulence markers, and antibiotic resistance markers to create a phylogeny tree of microbes and sets of k-mer fingerprints associated with different branches and leaves of the tree (61). The second part is a per-sample computation phase, where the system searches hundreds of millions of short sequence reads against the fingerprint sets to accurately detect and classify microorganisms in the sample (61). To prevent false positive identifications, the system applies a filtering threshold based on internal statistical scores that are determined by analyzing a wide range of metagenomes (61). This approach was also used to accurately detect genetic markers for virulence and antibiotic resistance.

### Preparation of MPN-qPCR samples

The MPN-qPCR assay was used to quantify *Vibrio* levels, which combines an enrichment step in alkaline peptone water (APW) with subsequent molecular detection by qPCR (19). From each oyster homogenate and water sample, three 1 mL aliquots were transferred to three tubes containing 9 mL of APW. Additionally, 1 mL of each homogenate and water sample was diluted with 9 mL of PBS and serially diluted up to 10^−8^ and 10^−6^, respectively. From each dilution, three 1 mL aliquots were inoculated into three tubes, each containing 9 mL of APW. Subsequently, all inoculated tubes were incubated overnight at 37°C.

After incubation, cell lysis was performed by transferring 1 mL of each positive (turbid) tube into a 1.5 mL microcentrifuge tube. The tubes were placed in a heat block for 10 minutes at 100°C. Boiled tubes were immersed immediately in ice until they were cold. Samples were centrifuged for 2 minutes at 14,000 × *g*, then frozen at −80°C until the qPCR detection of total *V. parahaemolyticus (tlh* gene) and total *V. vulnificus* (*vvh*A gene) was performed (62, 63). Samples testing positive for either species were subjected to further qPCR testing for *V. vulnificus* virulence correlated (*vcgC*) gene (62, 64) and *V. parahaemolyticus* pathogenic gene markers, thermostable direct hemolysin (*tdh*) and thermostable related hemolysin (*trh*) (63, 65) using two-stage qPCR cycling parameters described previously (62–64). MPN values were determined based on the qPCR results using the MPN method outlined in the Bacterial Analytical Manual of the US Food and Drug Administration (58).

### Data and statistical analysis

The alpha diversity of richness and evenness for oyster microbiomes within each sample type was assessed using the Simpson index. To determine if there were significant differences in alpha diversities between the sample types, a non-parametric Wilcoxon Rank Sum test, which does not assume normality, was used for pairwise comparisons, and a significance level of (*P* < 0.05) was used (61). To evaluate the similarities of the microbial community between sample types (beta diversity), the Bray-Curtis index was utilized. Statistical significance in beta diversities was determined using PERMANOVA (61). Furthermore, the abundance distribution and any significant differences in individual taxa with respect to sample type were identified using the Wilcoxon Rank Sum test (61). LDA effect size (LEfSe) was applied to identify biomarkers showing statistically significant and biologically consistent differences among the oyster sample types (LDA score > 2; *P* < 0.05) (61). LEfSe analysis was performed using the standard workflow described by Segata et al. (66). Briefly, the Kruskal-Wallis test identified the differentially abundant features, followed by a multiple testing correlation (false discovery rate). LDA was then used to estimate the effect size. To statistically assess the LogMPN level of total and pathogenic *V. parahaemolyticus* and *V. vulnificus* between practices, the Mann-Whitney test was used for pairwise comparisons, and a significance level of *P* <0.05 was used. To enhance the visualization of less dominant community members, a log abundance score transformation was applied.

## Data Availability

Raw sequence reads have been deposited in the NCBI database under BioProject number PRJNA1036668, accession numbers SAMN38147177 to SAMN38147182, SAMN38147192 to SAMN38147197, SAMN38147210 to SAMN38147215, and PRJNA1069116, accession numbers SAMN39612594 to SAMN39612647.

## SUPPLEMENTAL MATERIAL

Data Set S1 aem.01985-25-s0001.xlsx Abundance scores of microbial taxa, antibiotic-resistant genes, and virulence factors in addition to MPN-qPCR values and water parameters.

Supplemental figures aem.01985-25-s0002.pdf Figures S1 to S26.

ASM does not own the copyrights to Supplemental Material that may be linked to, or accessed through, an article. The authors have granted ASM a non-exclusive, world-wide license to publish the Supplemental Material files. Please contact the corresponding author directly for reuse.

## References

[1] Parveen S, Hettiarachchi KA, Bowers JC, Jones JL, Tamplin ML, McKay R, Beatty W, Brohawn K, Dasilva LV, Depaola A. 2008. Seasonal distribution of total and pathogenic Vibrio parahaemolyticus in Chesapeake Bay oysters and waters. Int J Food Microbiol 128:354–361. doi:10.1016/j.ijfoodmicro.2008.09.019 18963158

[2] Johnson CN, Bowers JC, Griffitt KJ, Molina V, Clostio RW, Pei S, Laws E, Paranjpye RN, Strom MS, Chen A, Hasan NA, Huq A, Noriea NF 3rd, Grimes DJ, Colwell RR. 2012. Ecology of Vibrio parahaemolyticus and Vibrio vulnificus in the coastal and estuarine waters of Louisiana, Maryland, Mississippi, and Washington (United States). Appl Environ Microbiol 78:7249–7257. doi:10.1128/AEM.01296-12 22865080 PMC3457101

[3] Froelich BA, Noble RT. 2016. Vibrio bacteria in raw oysters: managing risks to human health. Philos Trans R Soc Lond B Biol Sci 371:20150209. doi:10.1098/rstb.2015.0209 26880841 PMC4760139

[4] Parveen S, Jacobs J, Ozbay G, Chintapenta LK, Almuhaideb E, Meredith J, Ossai S, Abbott A, Grant A, Brohawn K, Chigbu P, Richards GP. 2020. Seasonal and geographical differences in total and pathogenic Vibrio parahaemolyticus and Vibrio vulnificus levels in seawater and oysters from the delaware and chesapeake bays determined using several methods. Appl Environ Microbiol 86:e01581–20. doi:10.1128/AEM.01581-20 32978135 PMC7657622

[5] Food and Agriculture Organization of the United Nations, World Health Organization. 2020. Microbiological Risk Assessment Series No. 20. Risk assessment tools for Vibrio parahaemolyticus and Vibrio vulnificus associated with seafoods, p 31

[6] U.S. Food and Drug Administration. 2024. National shellfish sanitation program: guide for the control of molluscan shellfish-2023 revision

[7] U.S. Centers for Disease Control and Prevention (CDC). 2022. Surveillance for foodborne disease outbreaks, 1994-2020. U.S. Department of Health and Human Services, CDC, National Outbreak Reporting System (NORS). Available from: https://wwwn.cdc.gov/norsdashboard. Retrieved 10 Jan 2025.

[8] Delahoy MJ, Shah HJ, Weller DL, Ray LC, Smith K, McGuire S, Trevejo RT, Scallan Walter E, Wymore K, Rissman T, McMillian M, Lathrop S, LaClair B, Boyle MM, Harris S, Zablotsky-Kufel J, Houck K, Devine CJ, Lau CE, Tauxe RV, Bruce BB, Griffin PM, Payne DC. 2023. Preliminary incidence and trends of infections caused by pathogens transmitted commonly through food — foodborne diseases active surveillance network, 10 U.S. Sites, 2022. MMWR Morb Mortal Wkly Rep 72:701–706. doi:10.15585/mmwr.mm7226a1 37384552 PMC10328488

[9] U.S. Centers for Disease Control and Prevention FDASNF. 2017. FoodNet 2015 surveillance report (final data), on centers for disease control and prevention, foodborne diseases active surveillance network (FoodNet). Available from: https://www.cdc.gov/foodnet/reports/annual-reports-2015.html. Retrieved 10 Jan 2025.

[10] Maryland Department of Natural Resources. 2019. Maryland chesapeake bay oyster management plan (May 2019). Annapolis, MD

[11] Maryland Department of Natural Resources (MDNR). 2016. Oyster management review: 2010-2015. Available from: https://dnr.maryland.gov/fisheries/Documents/FiveYearOysterReport.pdf. Retrieved 10 Jan 2025.

[12] Walton WC, Davis JE, Chaplin GI, Rikard FS, Hanson TR, Waters P, Swann D. 2012. Off-bottom oyster farming. Available from: https://extension.rwfm.tamu.edu/wp-content/uploads/sites/8/2013/09/Off-Bottom-Oyster-Farming.pdf. Retrieved 10 Jan 2025.

[13] Canty R, Blackwood D, Noble R, Froelich B. 2020. A comparison between farmed oysters using floating cages and oysters grown on-bottom reveals more potentially human pathogenic Vibrio in the on-bottom oysters. Environ Microbiol 22:4257–4263. doi:10.1111/1462-2920.14948 32079036

[14] Cole KM, Supan J, Ramirez A, Johnson CN. 2015. Suspension of oysters reduces the populations of Vibrio parahaemolyticus and Vibrio vulnificus. Lett Appl Microbiol 61:209–213. doi:10.1111/lam.12449 26031606

[15] Scro AK, Westphalen J, Kite-Powell HL, Brawley JW, Smolowitz RM. 2022. The effect of off-bottom versus on-bottom oyster culture on total and pathogenic Vibrio spp. abundances in oyster tissue, water and sediment samples. Int J Food Microbiol 379:109870. doi:10.1016/j.ijfoodmicro.2022.109870 35961160

[16] Martínez‐Porchas M, Vargas‐Albores F. 2017. Microbial metagenomics in aquaculture: a potential tool for a deeper insight into the activity. Rev Aquac 9:42–56. doi:10.1111/raq.12102

[17] Wooley JC, Godzik A, Friedberg I. 2010. A primer on metagenomics. PLoS Comput Biol 6:e1000667. doi:10.1371/journal.pcbi.1000667 20195499 PMC2829047

[18] Quince C, Walker AW, Simpson JT, Loman NJ, Segata N. 2017. Shotgun metagenomics, from sampling to analysis. Nat Biotechnol 35:833–844. doi:10.1038/nbt.3935 28898207

[19] Kaysner CA, DePaola A Jr, Jones J. 2004. Bacteriological analytical manual (BAM): Vibrio. Available from: https://www.fda.gov/food/laboratory-methods-food/bam-chapter-9-vibrio. Retrieved 10 Jan 2025.

[20] Cook DW, Ruple AD. 1989. Indicator bacteria and Vibrionaceae multiplication in post-harvest shellstock oysters. J Food Prot 52:343–349. doi:10.4315/0362-028X-52.5.343 31003275

[21] DePaola A, Hopkins LH, Peeler JT, Wentz B, McPhearson RM. 1990. Incidence of Vibrio parahaemolyticus in U.S. coastal waters and oysters. Appl Environ Microbiol 56:2299–2302. doi:10.1128/aem.56.8.2299-2302.1990 2403249 PMC184726

[22] Cook DW. 1997. Refrigeration of oyster shellstock: conditions which minimize the outgrowth of Vibrio vulnificus. J Food Prot 60:349–352. doi:10.4315/0362-028X-60.4.349 31195541

[23] Ellison RK, Malnati E, Depaola A, Bowers J, Rodrick GE. 2001. Populations of Vibrio parahaemolyticus in retail oysters from Florida using two methods. J Food Prot 64:682–686. doi:10.4315/0362-028x-64.5.682 11348000

[24] Fernandez-Piquer J, Bowman JP, Ross T, Tamplin ML. 2012. Molecular analysis of the bacterial communities in the live Pacific oyster (Crassostrea gigas) and the influence of postharvest temperature on its structure. J Appl Microbiol 112:1134–1143. doi:10.1111/j.1365-2672.2012.05287.x 22429335

[25] Ostrensky A, Horodesky A, Faoro H, Balsanelli E, Sfeir MZT, Cozer N, Pie MR, Dal Pont G, Castilho-Westphal GG. 2018. Metagenomic evaluation of the effects of storage conditions on the bacterial microbiota of oysters Crassostrea gasar (Adanson, 1757). J Appl Microbiol 125:1435–1443. doi:10.1111/jam.14045 29992707

[26] Morris JG, Acheson D. 2003. Cholera and other types of vibriosis: a story of human pandemics and oysters on the half shell. Clin Infect Dis 37:272–280. doi:10.1086/375600 12856219

[27] Unzueta-Martínez A, Girguis PR. 2025. Taxonomic diversity and functional potential of microbial communities in oyster calcifying fluid. Appl Environ Microbiol 91:e0109424. doi:10.1128/aem.01094-24 39665561 PMC11784444

[28] Brumfield KD, Enke S, Swan BK, Carrasquilla L, Lee MD, Stern DB, Gieser L, Hasan NA, Usmani M, Jutla AS, Huq A, Caviness K, Goodrich JS, Bull R, Colwell RR. 2025. Hybridization capture sequencing for Vibrio spp. and associated virulence factors. mBio 16:e0051625. doi:10.1128/mbio.00516-25 40558084 PMC12345146

[29] Pathak A, Marquez M, Stothard P, Chukwujindu C, Su J-Q, Zhou Y, Zhou X-Y, Jagoe CH, Chauhan A. 2024. A seasonal study on the microbiomes of diploid vs. triploid eastern oysters and their denitrification potential. iScience 27:110193. doi:10.1016/j.isci.2024.110193 38984199 PMC11231605

[30] Pimentel ZT, Dufault-Thompson K, Russo KT, Scro AK, Smolowitz RM, Gomez-Chiarri M, Zhang Y. 2021. Microbiome analysis reveals diversity and function of Mollicutes associated with the eastern oyster, Crassostrea virginica. mSphere 6:e00227-21. doi:10.1128/mSphere.00227-21 33980678 PMC8125052

[31] Bloomfield SJ, Zomer AL, O’Grady J, Kay GL, Wain J, Janecko N, Palau R, Mather AE. 2023. Determination and quantification of microbial communities and antimicrobial resistance on food through host DNA-depleted metagenomics. Food Microbiol 110:104162. doi:10.1016/j.fm.2022.104162 36462818

[32] Hasan MR, Rawat A, Tang P, Jithesh PV, Thomas E, Tan R, Tilley P. 2016. Depletion of human DNA in spiked clinical specimens for improvement of sensitivity of pathogen detection by next-generation sequencing. J Clin Microbiol 54:919–927. doi:10.1128/JCM.03050-15 26763966 PMC4809942

[33] Klosinska K, Reece E, Kenny E, Renwick J. 2022. Reducing human DNA bias in cystic fibrosis airway specimens for microbiome analysis. J Microbiol Methods 200:106540. doi:10.1016/j.mimet.2022.106540 35853495

[34] Marquet M, Zöllkau J, Pastuschek J, Viehweger A, Schleußner E, Makarewicz O, Pletz MW, Ehricht R, Brandt C. 2022. Evaluation of microbiome enrichment and host DNA depletion in human vaginal samples using Oxford Nanopore’s adaptive sequencing. Sci Rep 12:4000. doi:10.1038/s41598-022-08003-8 35256725 PMC8901746

[35] Islam Sajib MS, Brunker K, Oravcova K, Everest P, Murphy ME, Forde T. 2024. Advances in host depletion and pathogen enrichment methods for rapid sequencing–based diagnosis of bloodstream infectices in host depleon. J Mol Diagn 26:741–753. doi:10.1016/j.jmoldx.2024.05.008 38925458 PMC12178389

[36] Ali J, Bellankimath AB, Hira J, Chapagain C, Ahmad R. 2025. Development and optimization of the host DNA depletion in blood cultures using a saponin and SAN nucleases-based method. bioRxiv. doi:10.1101/2025.07.14.664698 PMC13099825 42027458

[37] Bruggeling CE, Garza DR, Achouiti S, Mes W, Dutilh BE, Boleij A. 2021. Optimized bacterial DNA isolation method for microbiome analysis of human tissues. Microbiologyopen 10:e1191. doi:10.1002/mbo3.1191 34180607 PMC8208965

[38] Marotz CA, Sanders JG, Zuniga C, Zaramela LS, Knight R, Zengler K. 2018. Improving saliva shotgun metagenomics by chemical host DNA depletion. Microbiome 6:42. doi:10.1186/s40168-018-0426-3 29482639 PMC5827986

[39] Marchukov D, Li J, Juillerat P, Misselwitz B, Yilmaz B. 2023. Benchmarking microbial DNA enrichment protocols from human intestinal biopsies. Front Genet 14:1184473. doi:10.3389/fgene.2023.1184473 37180976 PMC10169731

[40] Heravi FS, Zakrzewski M, Vickery K, Hu H. 2020. Host DNA depletion efficiency of microbiome DNA enrichment methods in infected tissue samples. J Microbiol Methods 170:105856. doi:10.1016/j.mimet.2020.105856 32007505

[41] Pathak A, Marquez M, Stothard P, Chukwujindu C, Su J-Q, Zhou Y, Zhou X-Y, Jagoe CH, Chauhan A. 2023. Microbiome analysis of the eastern oyster as a function of ploidy and seasons. bioRxiv. doi:10.1101/2023.08.10.552804

[42] Hines IS, Markov Madanick J, Smith SA, Kuhn DD, Stevens AM. 2023. Analysis of the core bacterial community associated with consumer-ready eastern oysters (Crassostrea virginica). PLoS One 18:e0281747. doi:10.1371/journal.pone.0281747 36812164 PMC9946220

[43] Pimentel ZT, Dufault-Thompson K, Russo KT, Scro AK, Smolowitz RM, Gomez-Chiarri M, Zhang Y. 2020. Diversity and function of the eastern oyster (Crassostrea virginica) microbiome. bioRxiv. doi:10.1101/2020.09.08.288811 PMC8125052 33980678

[44] Pierce ML, Ward JE. 2018. Microbial ecology of the Bivalvia, with an emphasis on the family ostreidae. J Shellfish Res 37:793–806. doi:10.2983/035.037.0410

[45] Pierce ML, Ward JE. 2019. Gut microbiomes of the eastern oyster (Crassostrea virginica) and the blue mussel (Mytilus edulis): temporal variation and the influence of marine aggregate-associated microbial communities. mSphere 4:e00730-19. doi:10.1128/mSphere.00730-19 31826972 PMC6908423

[46] Chauhan A, Wafula D, Lewis DE, Pathak A. 2014. Metagenomic assessment of the eastern oyster-associated microbiota. Genome Announc 2:e01083-14. doi:10.1128/genomeA.01083-14 PMC4208335 25342691

[47] King GM, Judd C, Kuske CR, Smith C. 2012. Analysis of stomach and gut microbiomes of the eastern oyster (Crassostrea virginica) from Coastal Louisiana, USA. PLoS One 7:e51475. doi:10.1371/journal.pone.0051475 23251548 PMC3520802

[48] Logares R, Sunagawa S, Salazar G, Cornejo-Castillo FM, Ferrera I, Sarmento H, Hingamp P, Ogata H, de Vargas C, Lima-Mendez G, Raes J, Poulain J, Jaillon O, Wincker P, Kandels-Lewis S, Karsenti E, Bork P, Acinas SG. 2014. Metagenomic 16S rDNA Illumina tags are a powerful alternative to amplicon sequencing to explore diversity and structure of microbial communities. Environ Microbiol 16:2659–2671. doi:10.1111/1462-2920.12250 24102695

[49] Clooney AG, Fouhy F, Sleator RD, O’ Driscoll A, Stanton C, Cotter PD, Claesson MJ. 2016. Comparing apples and oranges?: next generation sequencing and its impact on microbiome analysis. PLoS One 11:e0148028. doi:10.1371/journal.pone.0148028 26849217 PMC4746063

[50] Tessler M, Neumann JS, Afshinnekoo E, Pineda M, Hersch R, Velho LFM, Segovia BT, Lansac-Toha FA, Lemke M, DeSalle R, Mason CE, Brugler MR. 2017. Large-scale differences in microbial biodiversity discovery between 16S amplicon and shotgun sequencing. Sci Rep 7:6589. doi:10.1038/s41598-017-06665-3 28761145 PMC5537354

[51] Longhi G, Argentini C, Fontana F, Tarracchini C, Mancabelli L, Lugli GA, Alessandri G, Lahner E, Pivetta G, Turroni F, Ventura M, Milani C. 2024. Saponin treatment for eukaryotic DNA depletion alters the microbial DNA profiles by reducing the abundance of Gram-negative bacteria in metagenomics analyses. Microbiome Res Rep 3:4. doi:10.20517/mrr.2023.02 38455080 PMC10917613

[52] Dupont S, Lokmer A, Corre E, Auguet J-C, Petton B, Toulza E, Montagnani C, Tanguy G, Pecqueur D, Salmeron C, Guillou L, Desnues C, La Scola B, Bou Khalil J, de Lorgeril J, Mitta G, Gueguen Y, Escoubas J-M. 2020. Oyster hemolymph is a complex and dynamic ecosystem hosting bacteria, protists and viruses. Anim Microbiome 2:12. doi:10.1186/s42523-020-00032-w 33499958 PMC7807429

[53] Jiang J-Z, Fang Y-F, Wei H-Y, Zhu P, Liu M, Yuan W-G, Yang L-L, Guo Y-X, Jin T, Shi M, Yao T, Lu J, Ye L-T, Shi S-K, Wang M, Duan M, Zhang D-C. 2023. A remarkably diverse and well-organized virus community in a filter-feeding oyster. Microbiome 11:2. doi:10.1186/s40168-022-01431-8 36611217 PMC9825006

[54] Kaufman GE, Bej AK, Bowers J, DePaola A. 2003. Oyster-to-oyster variability in levels of Vibrio parahaemolyticus. J Food Prot 66:125–129. doi:10.4315/0362-028x-66.1.125 12540193

[55] Klein SL, Lovell CR. 2017. The hot oyster: levels of virulent Vibrio parahaemolyticus strains in individual oysters. FEMS Microbiol Ecol 93:fiw232. doi:10.1093/femsec/fiw232 27827805

[56] Bienlien LM, Audemard C, Reece KS, Carnegie RB. 2022. Impact of parasitism on levels of human-pathogenic Vibrio species in eastern oysters. J Appl Microbiol 132:760–771. doi:10.1111/jam.15287 34487403

[57] Audemard C, Reece KS, Latour RJ, Bienlien LM, Carnegie RB. 2023. Influence of oyster genetic background on levels of human-pathogenic Vibrio spp. Aquaculture 562:738763. doi:10.1016/j.aquaculture.2022.738763

[58] Blodgett R. 2023. Bacteriological analytical manual (BAM), appendix 2: most probable number from serial dilutions. U.S. Food & Drug Administration. Available from: https://www.fda.gov/food/laboratory-methods-food/bam-appendix-2-most-probable-number-serial-dilutions. Retrieved 10 Jan 2025.

[59] Almuhaideb E, Hasan NA, Grim C, Rashed SM, Parveen S. 2025. Comparative evaluation of specimen type and processing conditions for studying oyster microbiomes. Front Microbiol 15:1504487. doi:10.3389/fmicb.2024.1504487 39845046 PMC11750828

[60] Illumina. 2017. Accelerate your next breakthrough with our comprehensive methods guide. Illumina. Available from: https://www.illumina.com/landing/methods-guide.html. Retrieved 10 Jan 2025.

[61] CosmosID. 2022. CosmosID metagenomics cloud. cosmosID Inc. Available from: www.cosmosid.com. Retrieved 10 Jan 2025.

[62] Campbell MS, Wright AC. 2003. Real-time PCR analysis of Vibrio vulnificus from oysters. Appl Environ Microbiol 69:7137–7144. doi:10.1128/AEM.69.12.7137-7144.2003 14660359 PMC309878

[63] Nordstrom JL, Vickery MCL, Blackstone GM, Murray SL, DePaola A. 2007. Development of a multiplex real-time PCR assay with an internal amplification control for the detection of total and pathogenic Vibrio parahaemolyticus bacteria in oysters. Appl Environ Microbiol 73:5840–5847. doi:10.1128/AEM.00460-07 17644647 PMC2074920

[64] Baker-Austin C, Gore A, Oliver JD, Rangdale R, McArthur JV, Lees DN. 2010. Rapid in situ detection of virulent Vibrio vulnificus strains in raw oyster matrices using real-time PCR. Environ Microbiol Rep 2:76–80. doi:10.1111/j.1758-2229.2009.00092.x 23766001

[65] Shirai H, Ito H, Hirayama T, Nakamoto Y, Nakabayashi N, Kumagai K, Takeda Y, Nishibuchi M. 1990. Molecular epidemiologic evidence for association of thermostable direct hemolysin (TDH) and TDH-related hemolysin of Vibrio parahaemolyticus with gastroenteritis. Infect Immun 58:3568–3573. doi:10.1128/iai.58.11.3568-3573.1990 2228229 PMC313699

[66] Segata N, Izard J, Waldron L, Gevers D, Miropolsky L, Garrett WS, Huttenhower C. 2011. Metagenomic biomarker discovery and explanation. Genome Biol 12:R60. doi:10.1186/gb-2011-12-6-r60 21702898 PMC3218848

