# Supplementary material for: Effects of aquaculture practices on Vibrio population dynamics and oyster microbiome
Source: Appl Environ Microbiol. 2025 Dec 15;92(1):e01985-25. doi: 10.1128/aem.01985-25 (PMC12838246; doi:10.1128/aem.01985-25)
Supplement: Supplemental figures — Figures S1 to S26. [file aem.01985-25-s0002.pdf]

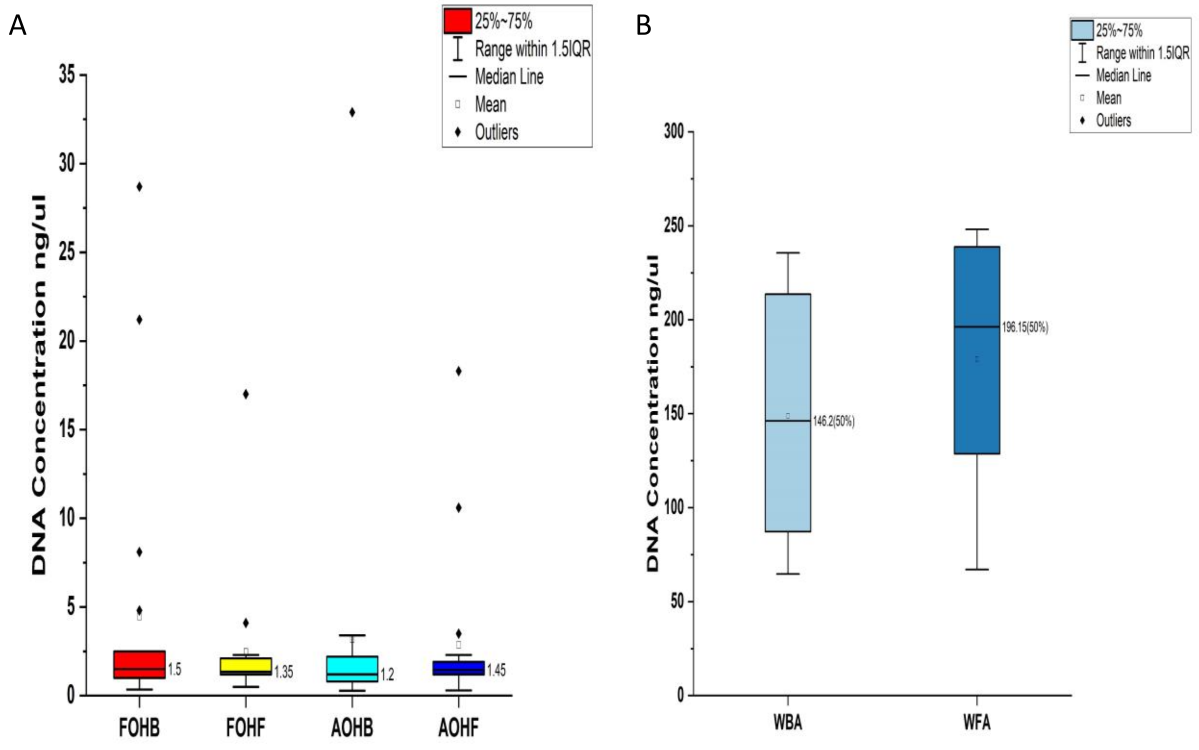

FIG S1: Concentration of DNA isolated from each sample type. A) oyster samples, B) water samples. FOHB: Fresh oyster homogenate from bottom cages; FOHF: Fresh oyster homogenate from floating cages; AOHB: Temperature-abused oyster homogenate from bottom cages; AOHF: Temperature-abused oyster homogenate from floating cages; WBA: Water from the bottom cages area; WFA: Water from the floating cages area.

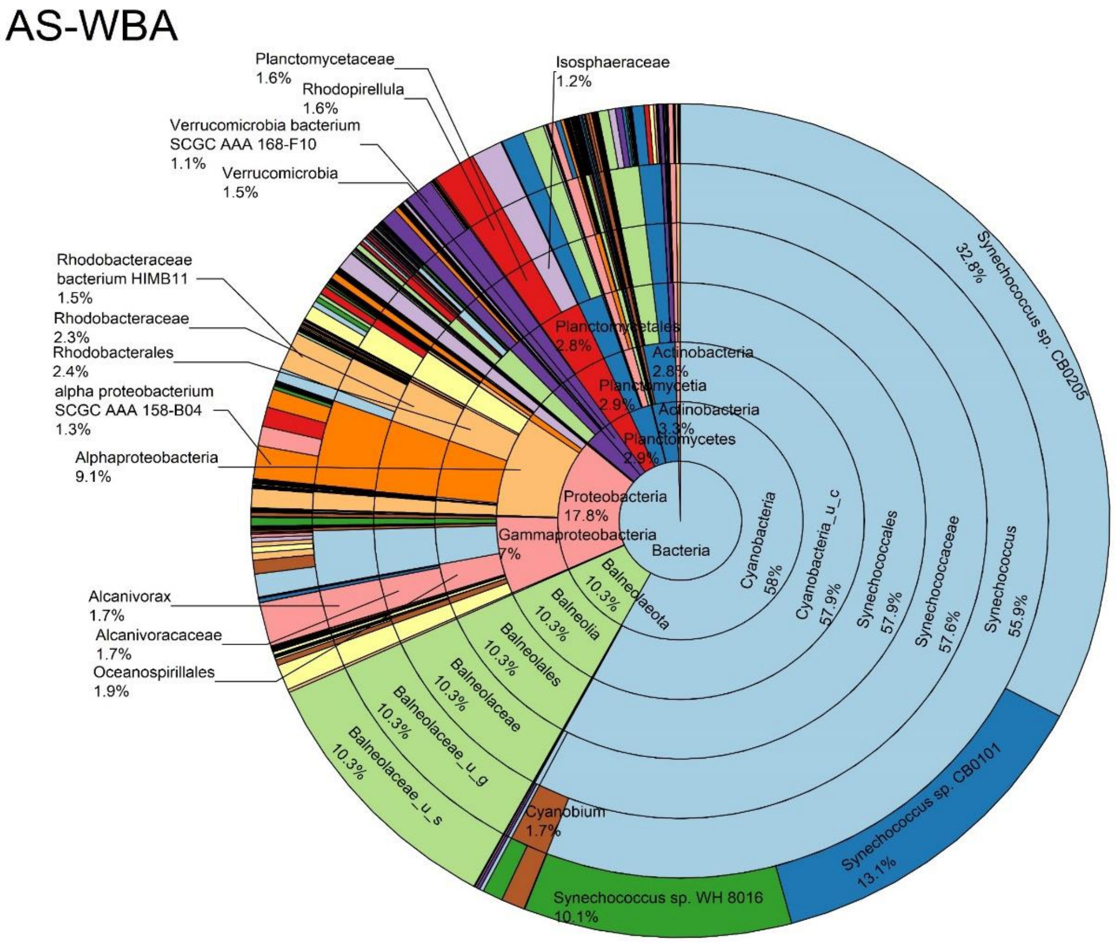

FIG S2: Relative abundance distribution of bacterial taxa per sample type. AS: abundance score. WBA: Water from the bottom cages area.

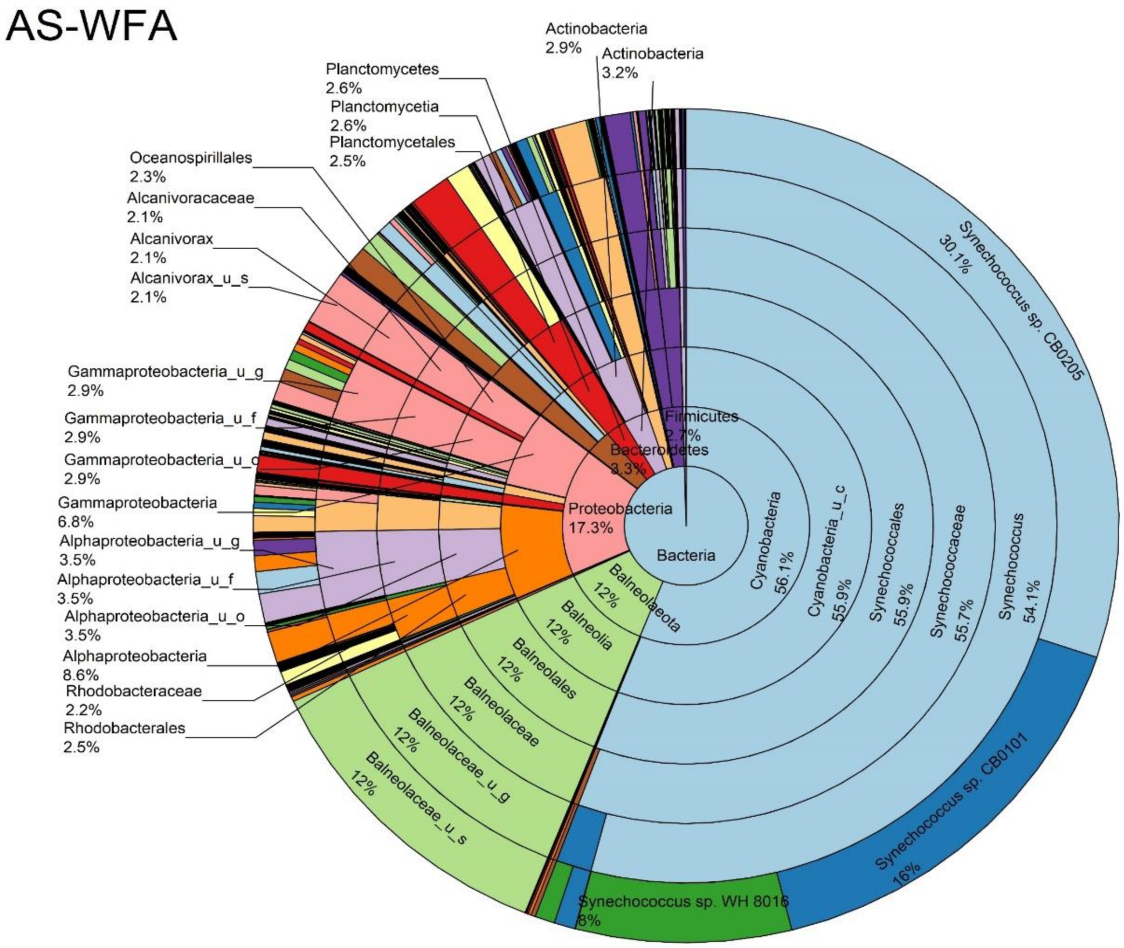

FIG S3: Relative abundance distribution of bacterial taxa per sample type. AS: abundance score. WFA: Water from the floating cages area.

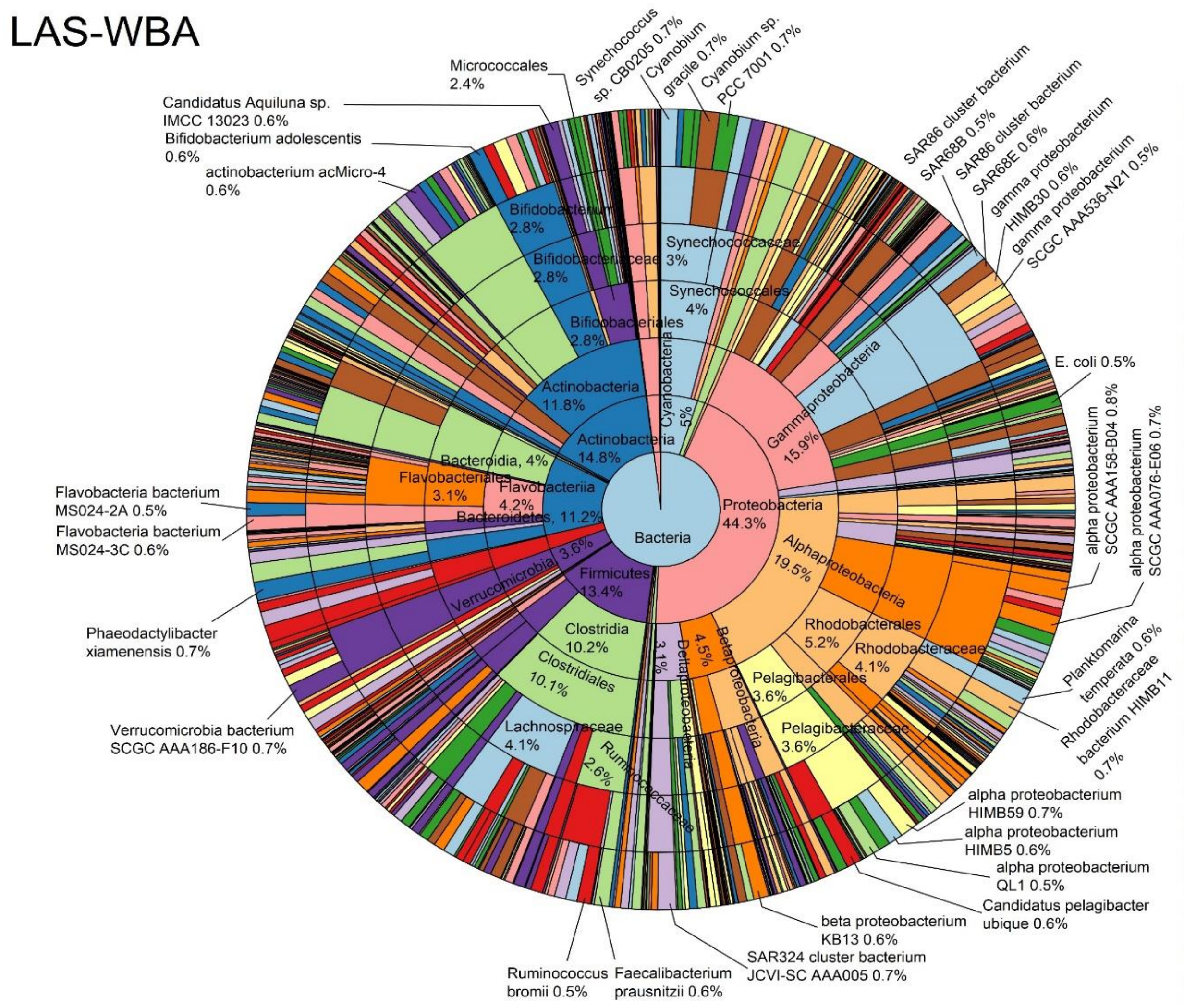

FIG S4: Relative log abundance distribution of bacterial taxa per sample type. LAS: log abundance score. WBA: Water from the bottom cages area.

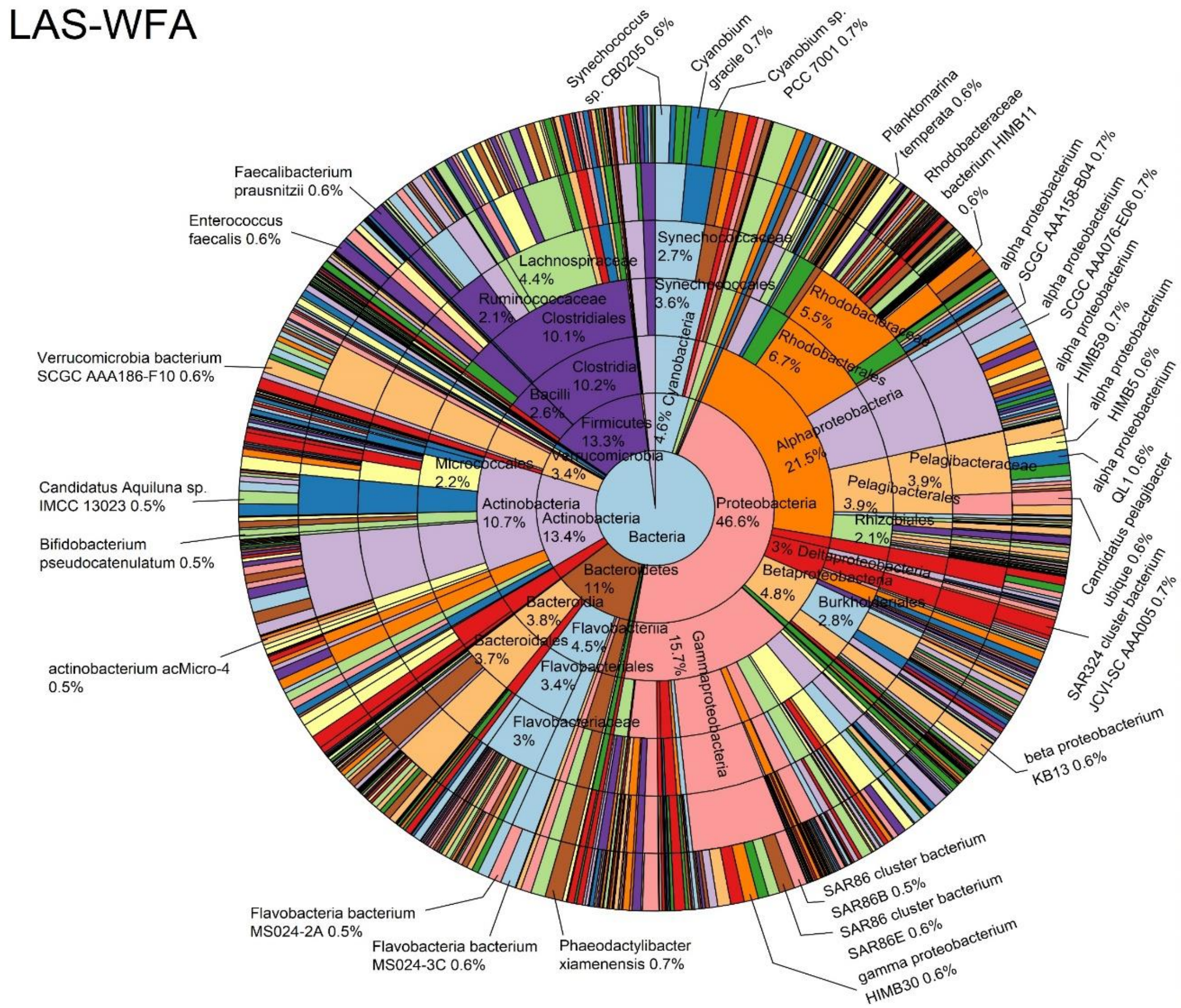

FIG S5: Relative log abundance distribution of bacterial taxa per sample type. LAS: log abundance score. WFA: Water from the floating cages area.

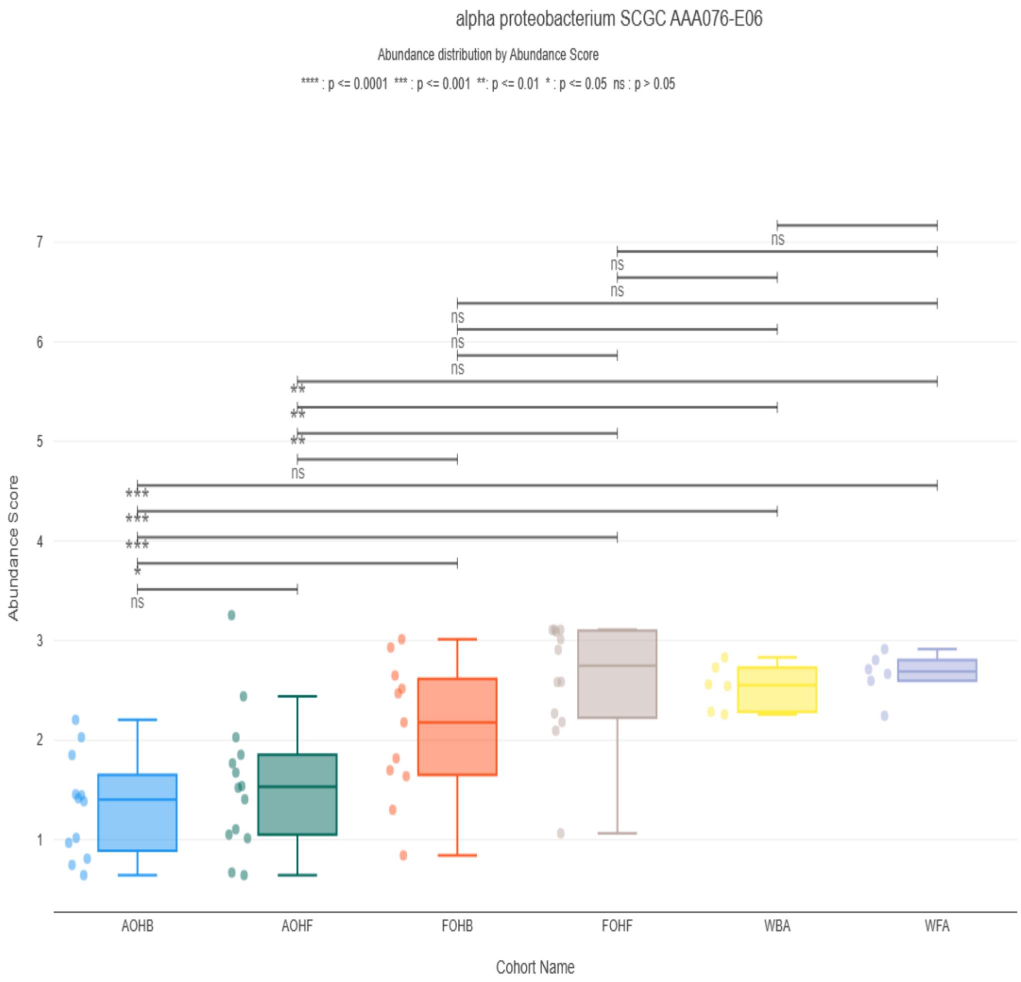

FIG S6: Log abundance distribution of alpha proteobacterium SCGC AAA076-E06 across all sample types.

FOHB: Fresh oyster homogenate from bottom cages; FOHF: Fresh oyster homogenate from floating cages; AOHB: Temperature-abused oyster homogenate from bottom cages; AOHF: Temperature-abused oyster homogenate from floating cages; WBA: Water from the bottom cages area; WFA: Water from the floating cages area.

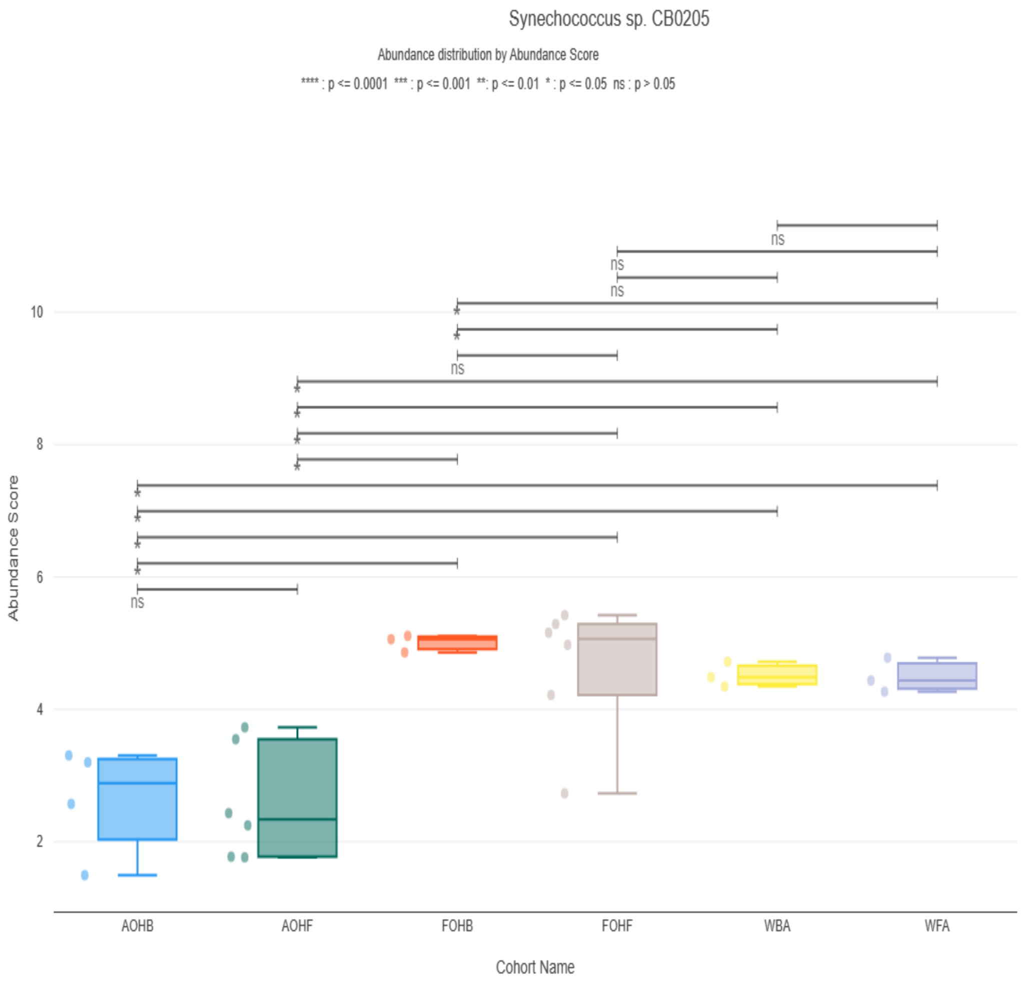

FIG S7: Log abundance distribution of *Synechococcus* sp. CB0205 across all sample types.

FOHB: Fresh oyster homogenate from bottom cages; FOHF: Fresh oyster homogenate from floating cages; AOHB: Temperature-abused oyster homogenate from bottom cages; AOHF: Temperature-abused oyster homogenate from floating cages; WBA: Water from the bottom cages area; WFA: Water from the floating cages area.

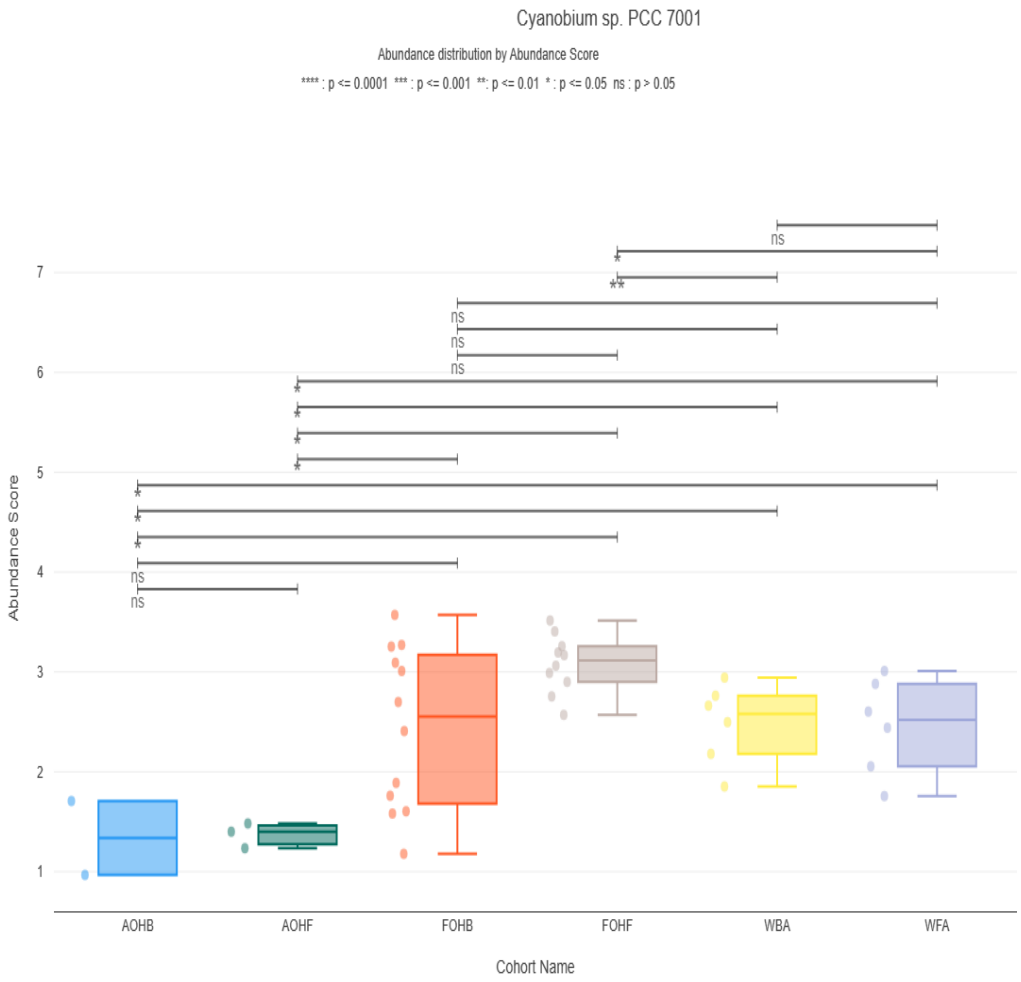

FIG S8: Log abundance distribution of *Cyanobium* sp. PCC7001 across all sample types.

FOHB: Fresh oyster homogenate from bottom cages; FOHF: Fresh oyster homogenate from floating cages; AOHB: Temperature-abused oyster homogenate from bottom cages; AOHF: Temperature-abused oyster homogenate from floating cages; WBA: Water from the bottom cages area; WFA: Water from the floating cages area.

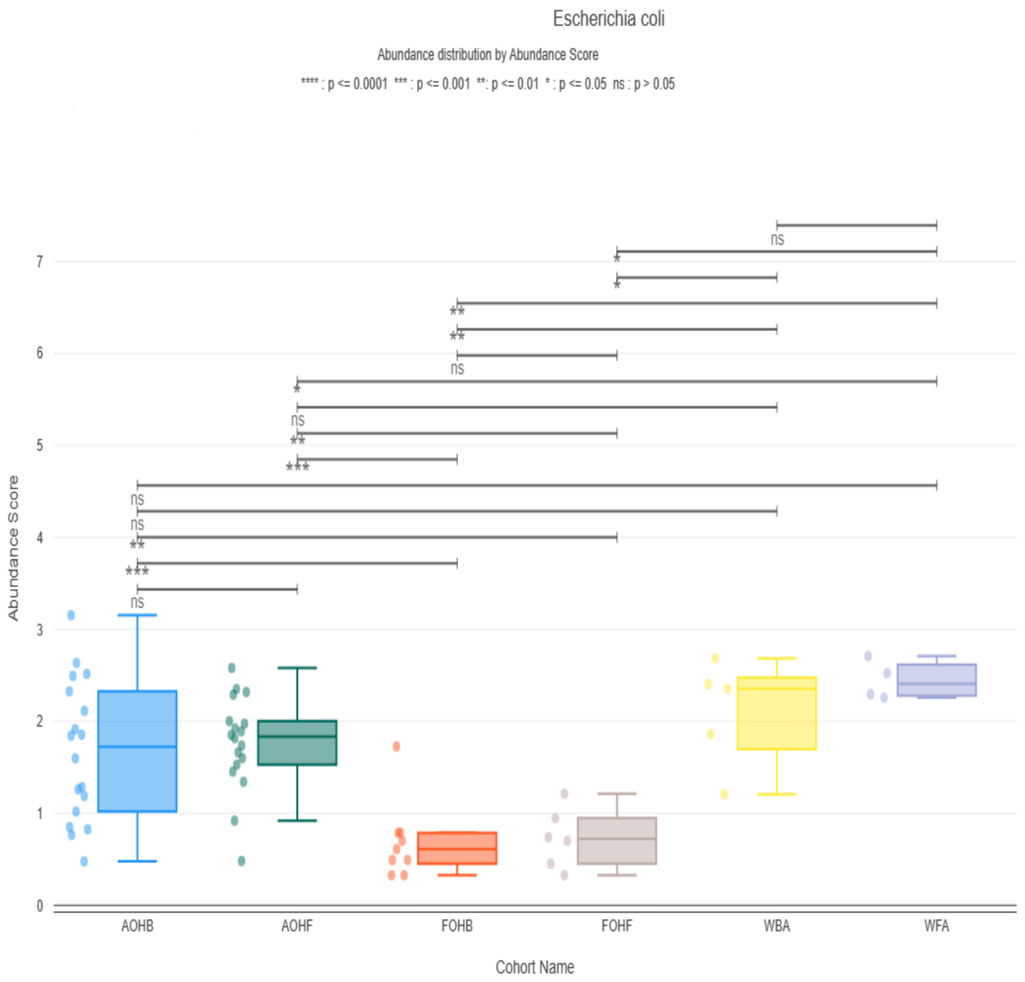

FIG S9: Log abundance distribution of *E. coli* across all sample types.

FOHB: Fresh oyster homogenate from bottom cages; FOHF: Fresh oyster homogenate from floating cages; AOHB: Temperature-abused oyster homogenate from bottom cages; AOHF: Temperature-abused oyster homogenate from floating cages; WBA: Water from the bottom cages area; WFA: Water from the floating cages area.

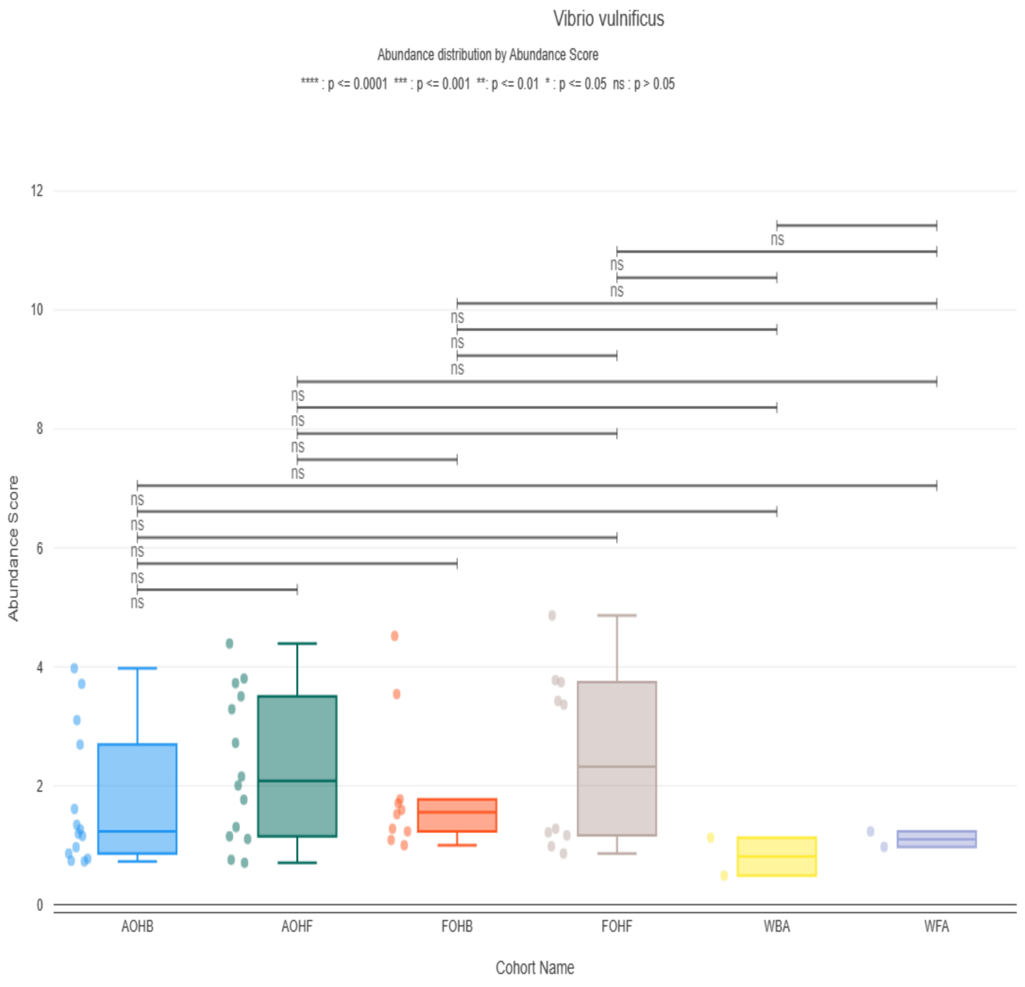

FIG S10: Log abundance distribution of *V. vulnificus* across all sample types.

FOHB: Fresh oyster homogenate from bottom cages; FOHF: Fresh oyster homogenate from floating cages; AOHB: Temperature-abused oyster homogenate from bottom cages; AOHF: Temperature-abused oyster homogenate from floating cages; WBA: Water from the bottom cages area; WFA: Water from the floating cages area.

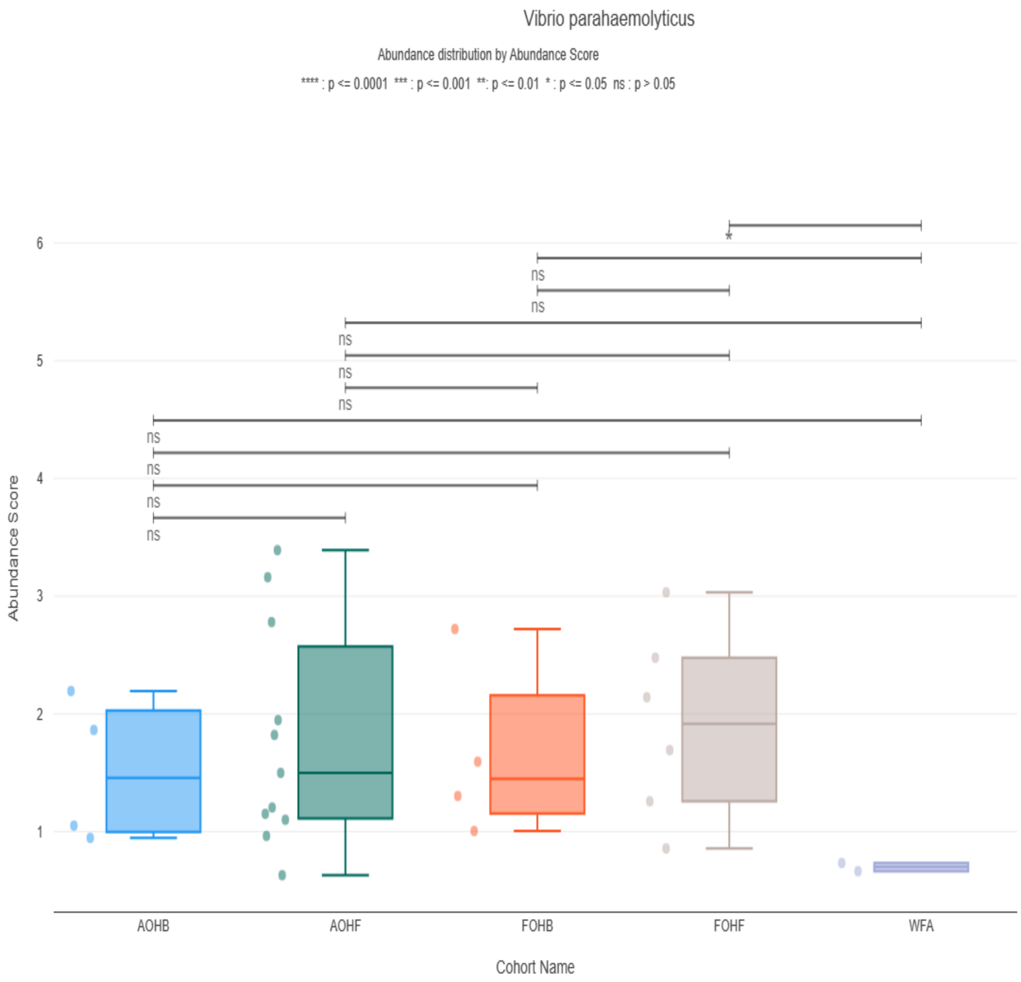

FIG S11: Log abundance distribution of *V. parahaemolyticus* across all sample types.

FOHB: Fresh oyster homogenate from bottom cages; FOHF: Fresh oyster homogenate from floating cages; AOHB: Temperature-abused oyster homogenate from bottom cages; AOHF: Temperature-abused oyster homogenate from floating cages; WFA: Water from the floating cages area.

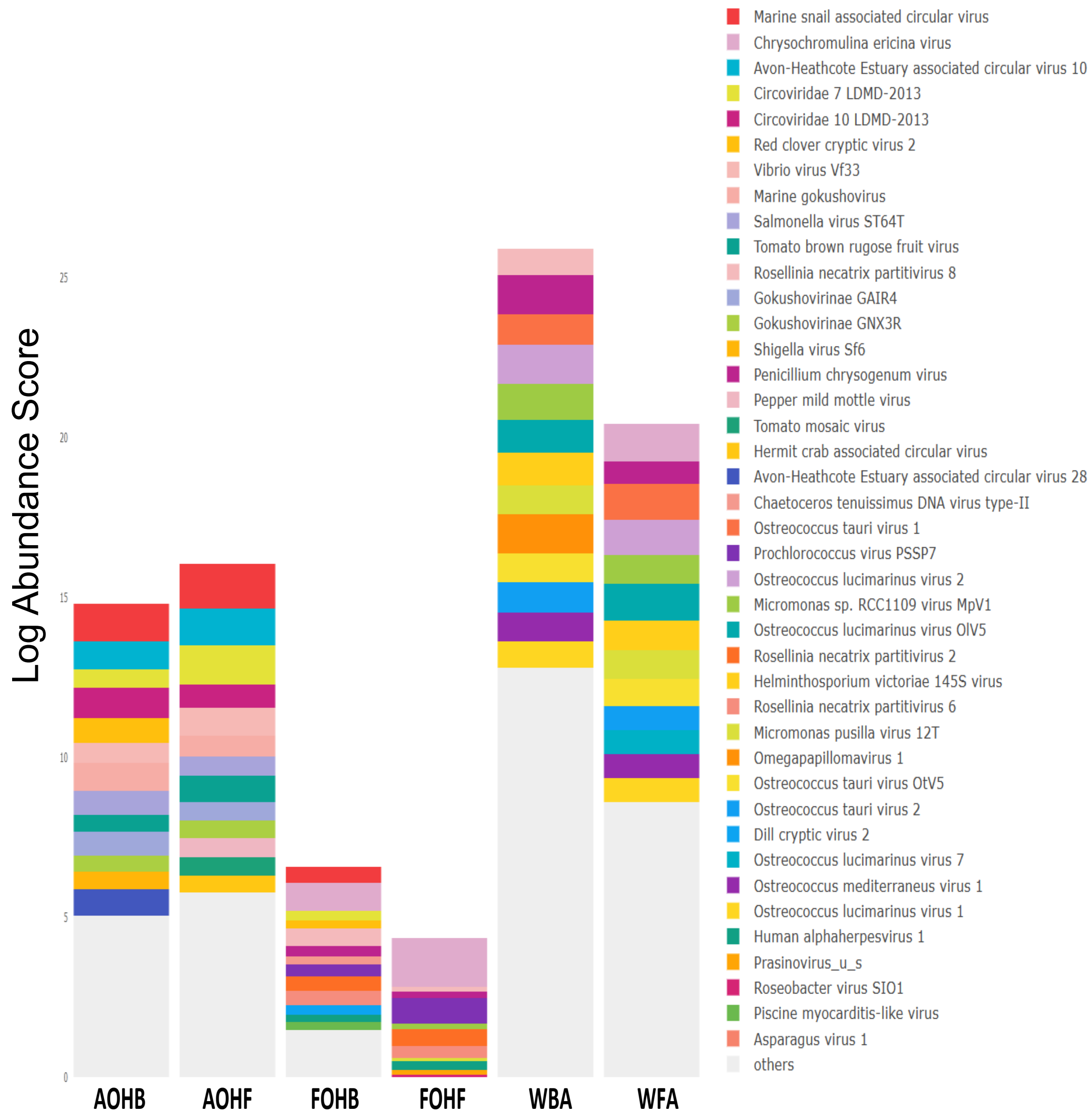

FIG S12: Relative log-abundance of viruses per sample type.

FOHB: Fresh oyster homogenate from bottom cages; FOHF: Fresh oyster homogenate from floating cages; AOHB: Temperature-abused oyster homogenate from bottom cages; AOHF: Temperature-abused oyster homogenate from floating cages; WBA: Water from the bottom cages area; WFA: Water from the floating cages area.

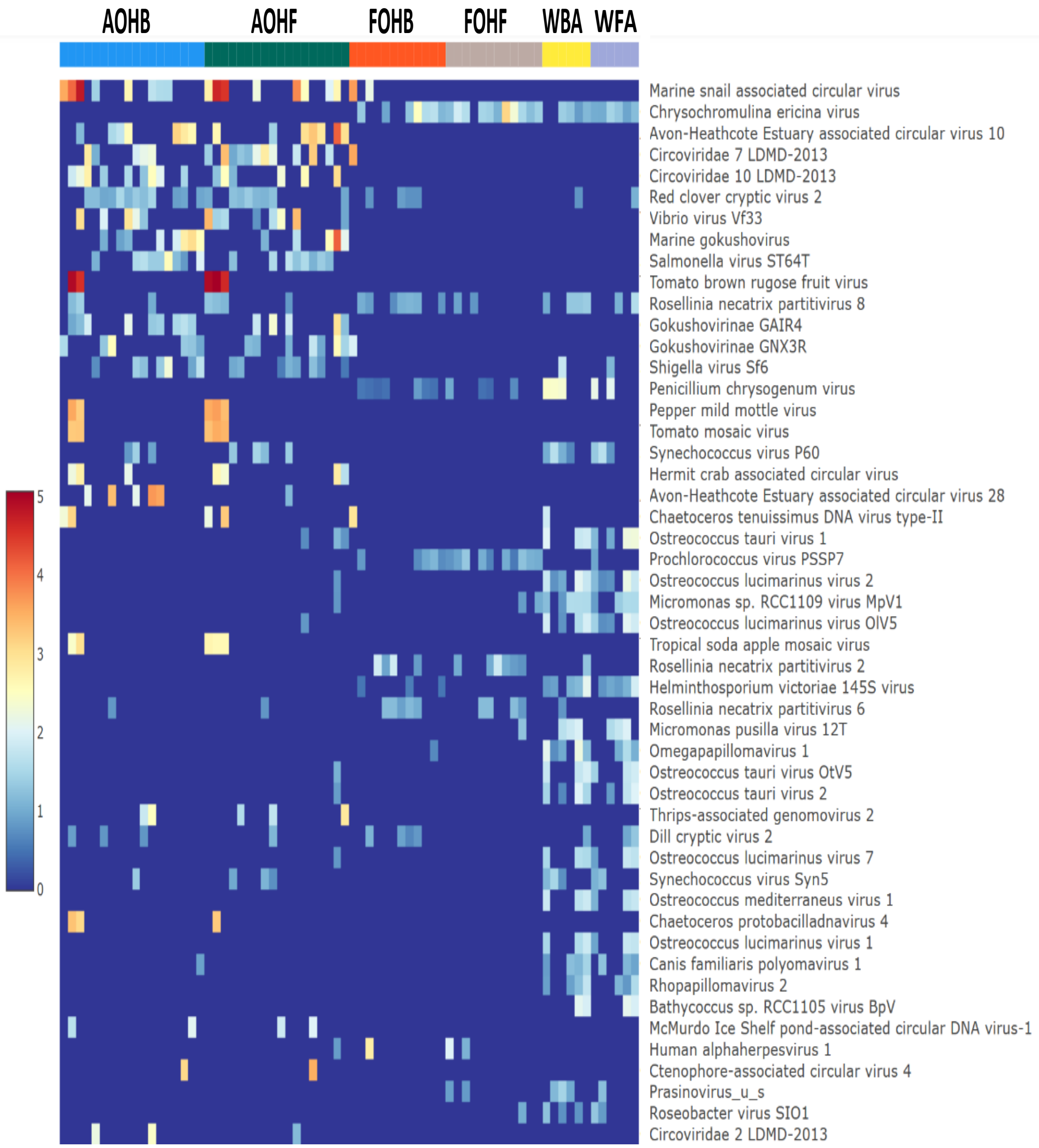

FIG S13: Mean log-abundance of viruses across all sample types.

FOHB: Fresh oyster homogenate from bottom cages; FOHF: Fresh oyster homogenate from floating cages; AOHB: Temperature-abused oyster homogenate from bottom cages; AOHF: Temperature-abused oyster homogenate from floating cages; WBA: Water from the bottom cages area; WFA: Water from the floating cages area.

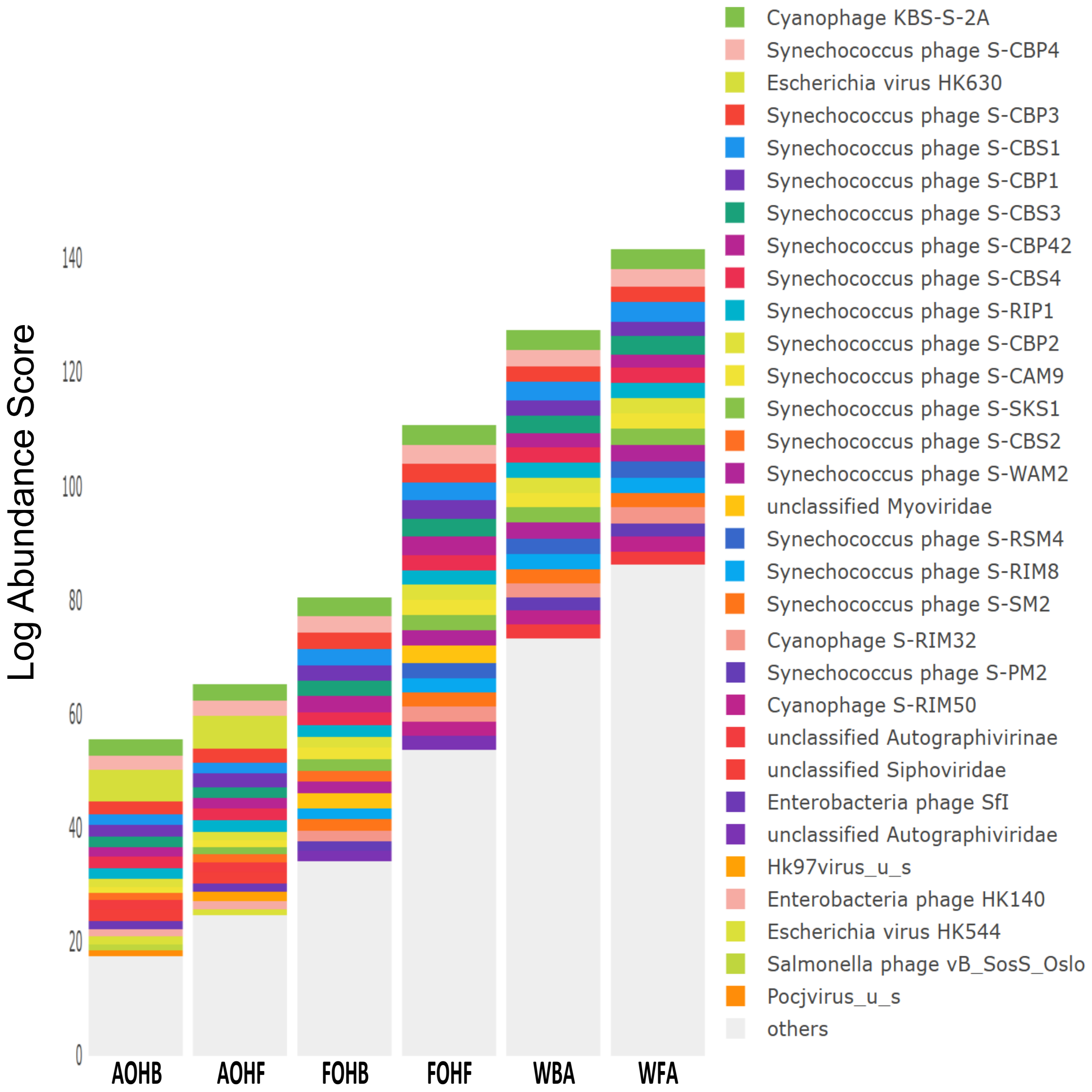

FIG S14: Relative log-abundance of phages per sample type.

FOHB: Fresh oyster homogenate from bottom cages; FOHF: Fresh oyster homogenate from floating cages; AOHB: Temperature-abused oyster homogenate from bottom cages; AOHF: Temperature-abused oyster homogenate from floating cages; WBA: Water from the bottom cages area; WFA: Water from the floating cages area.

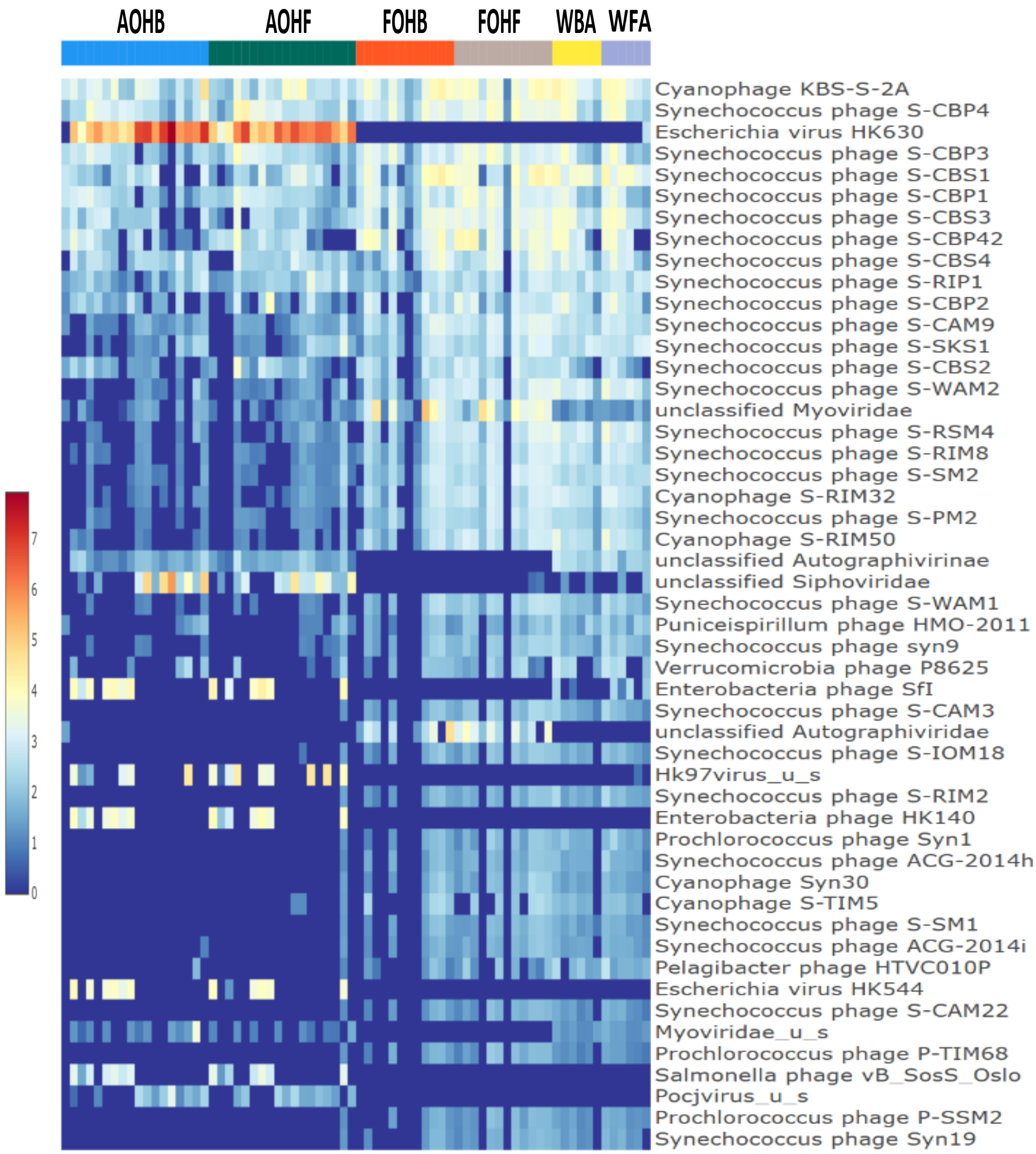

FIG S15: Mean log-abundance of phages across all sample types.

FOHB: Fresh oyster homogenate from bottom cages; FOHF: Fresh oyster homogenate from floating cages; AOHB: Temperature-abused oyster homogenate from bottom cages; AOHF: Temperature-abused oyster homogenate from floating cages; WBA: Water from the bottom cages area; WFA: Water from the floating cages area.

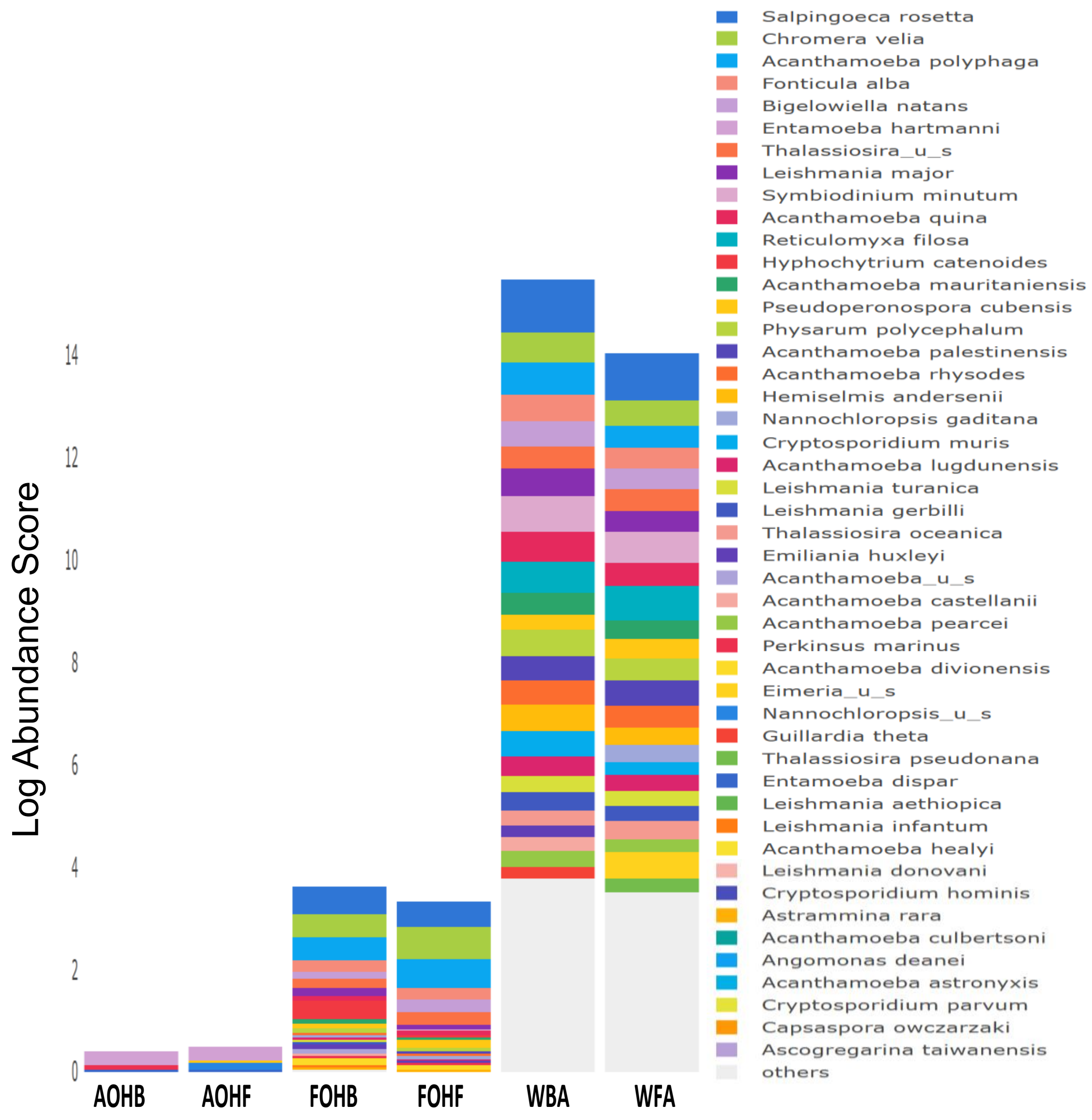

FIG S16: Relative log-abundance of protists per sample type.

FOHB: Fresh oyster homogenate from bottom cages; FOHF: Fresh oyster homogenate from floating cages; AOHB: Temperature-abused oyster homogenate from bottom cages; AOHF: Temperature-abused oyster homogenate from floating cages; WBA: Water from the bottom cages area; WFA: Water from the floating cages area.

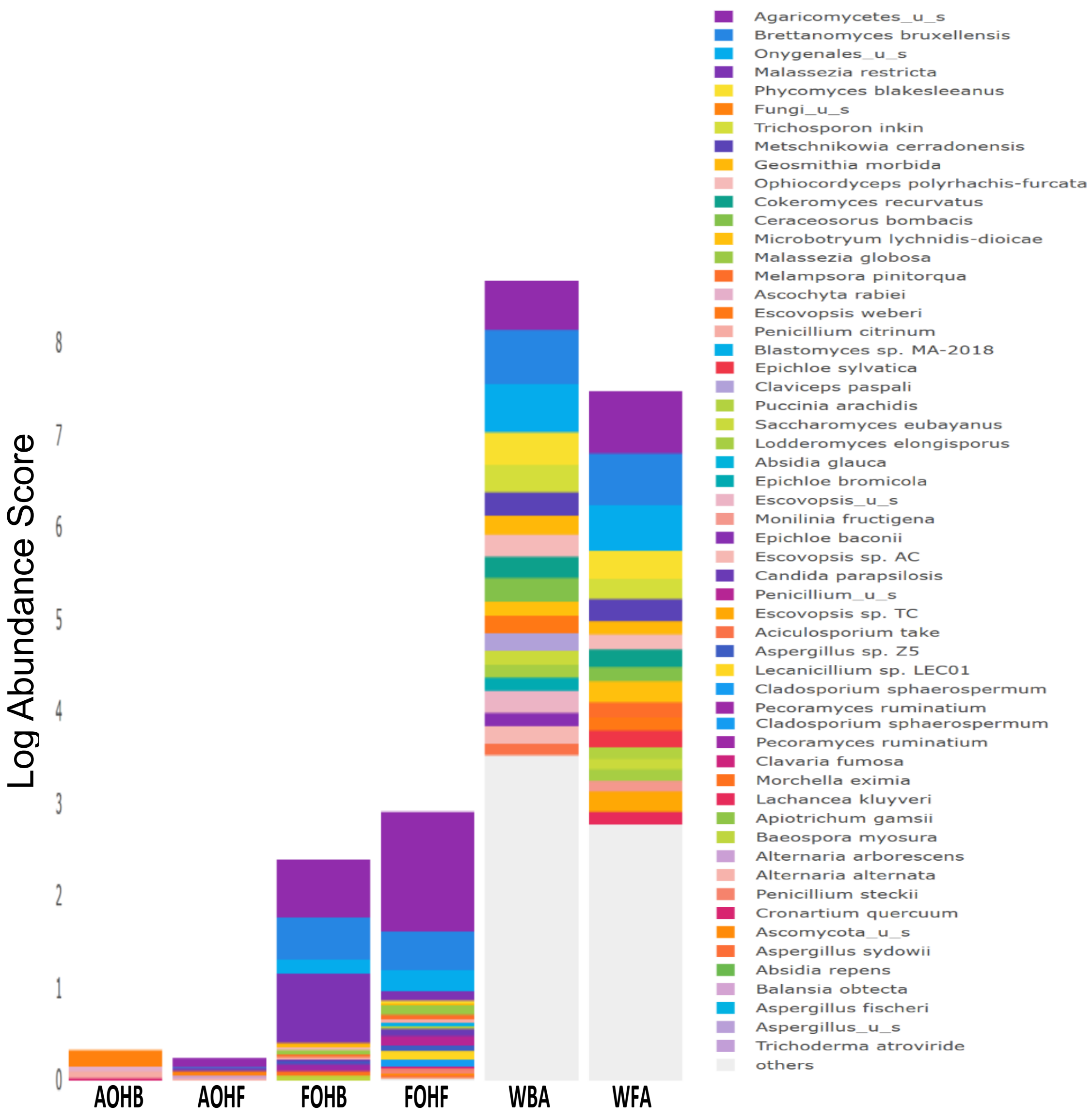

FIG S17: Relative log-abundance of fungi per sample type.

FOHB: Fresh oyster homogenate from bottom cages; FOHF: Fresh oyster homogenate from floating cages; AOHB: Temperature-abused oyster homogenate from bottom cages; AOHF: Temperature-abused oyster homogenate from floating cages; WBA: Water from the bottom cages area; WFA: Water from the floating cages area.

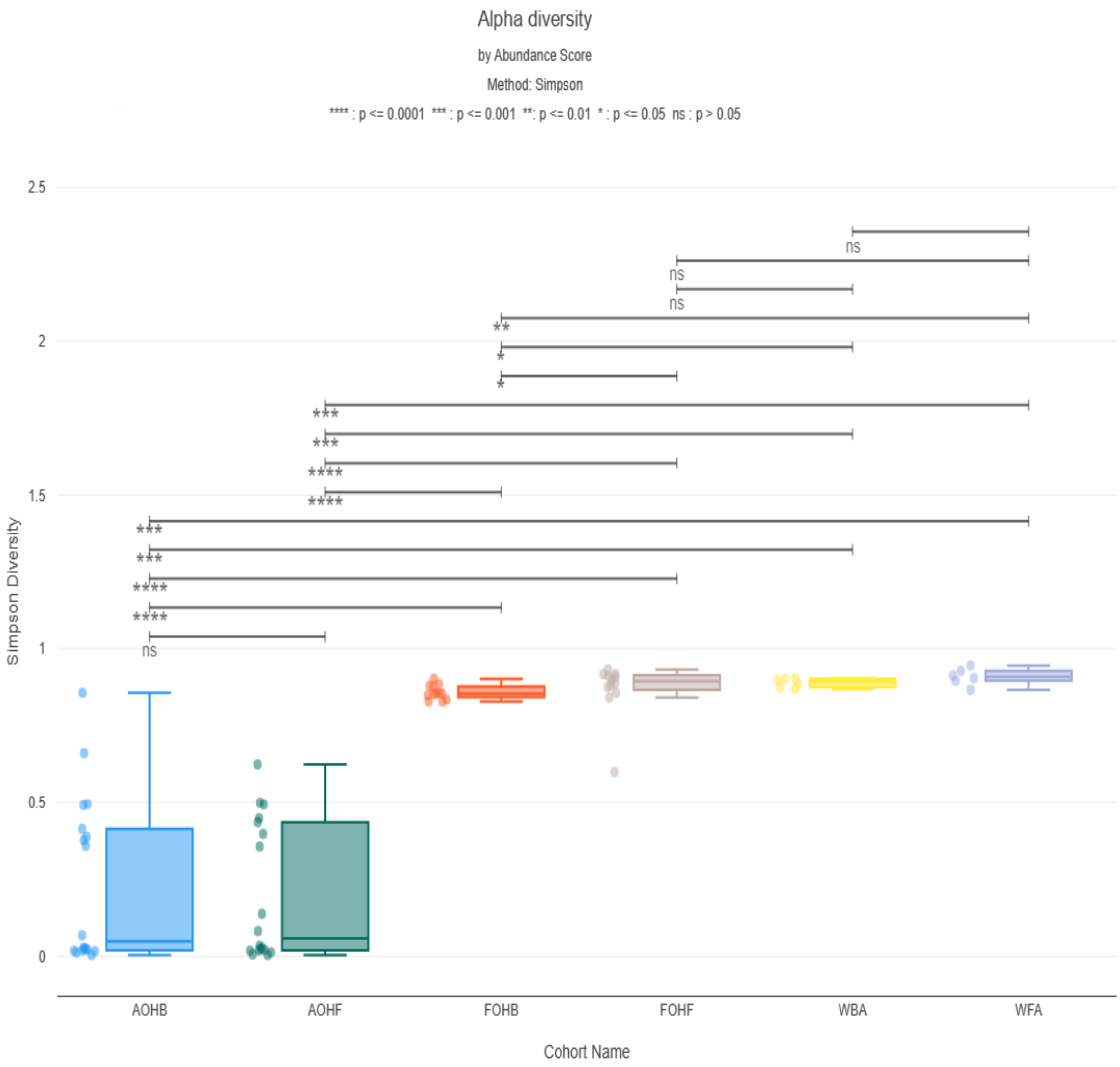

FIG S18: Simpson index representing the richness and evenness of phages within all sample types. Each dot represents the Simpson diversity value for an individual sample. Statistical comparisons of medians across sample types were performed using a Wilcoxon rank-sum test. FOHB: Fresh oyster homogenate from bottom cages; FOHF: Fresh oyster homogenate from floating cages; AOHB: Temperature-abused oyster homogenate from bottom cages; AOHF: Temperature-abused oyster homogenate from floating cages; WBA: Water from the bottom cages area; WFA: Water from the floating cages area.

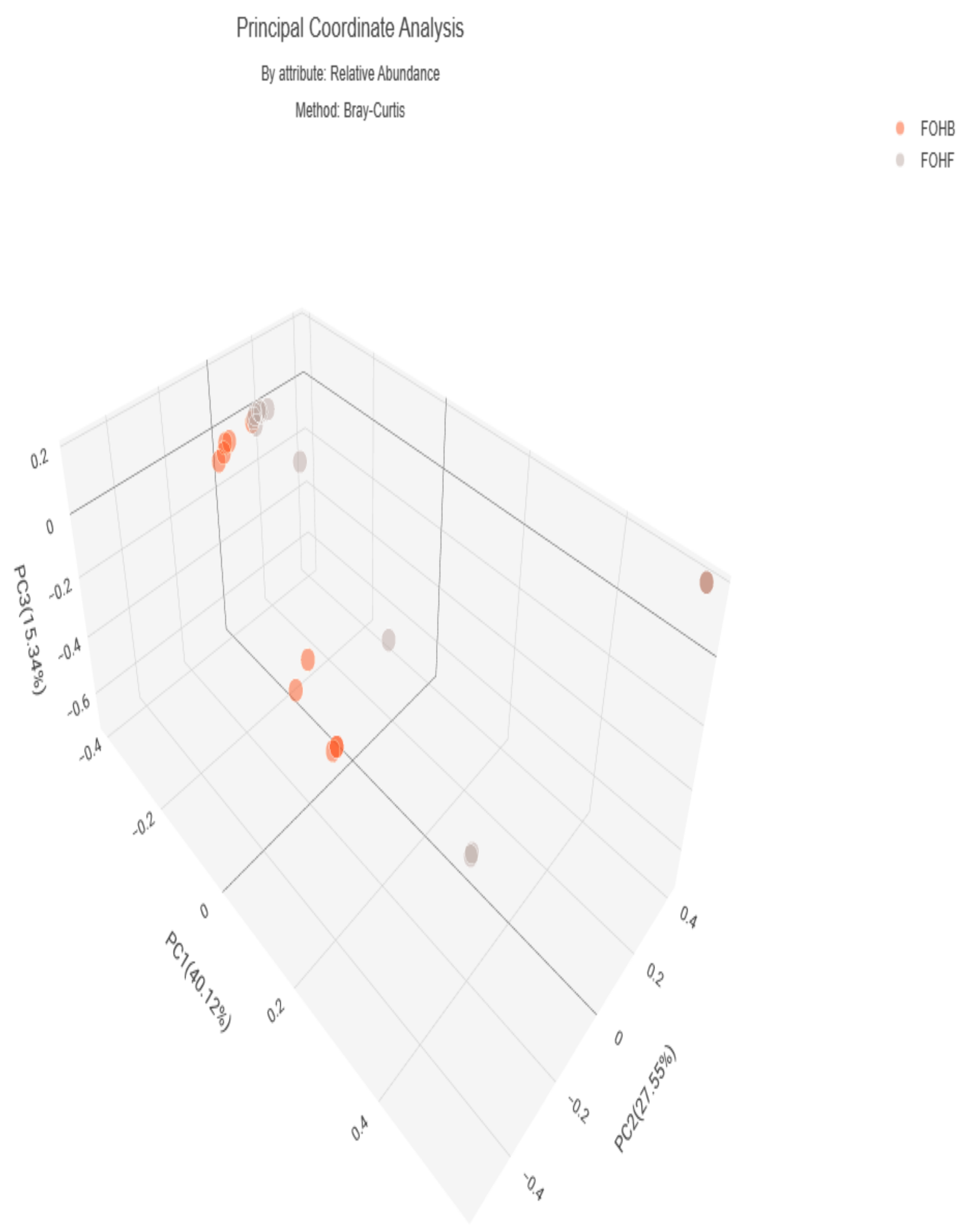

FIG S19: Bray-Curtis index representing the significant differences of fungi composition and their relative abundance between FOHB and FOHF.

FOHB: Fresh oyster homogenate from bottom cages; FOHF: Fresh oyster homogenate from floating cages.

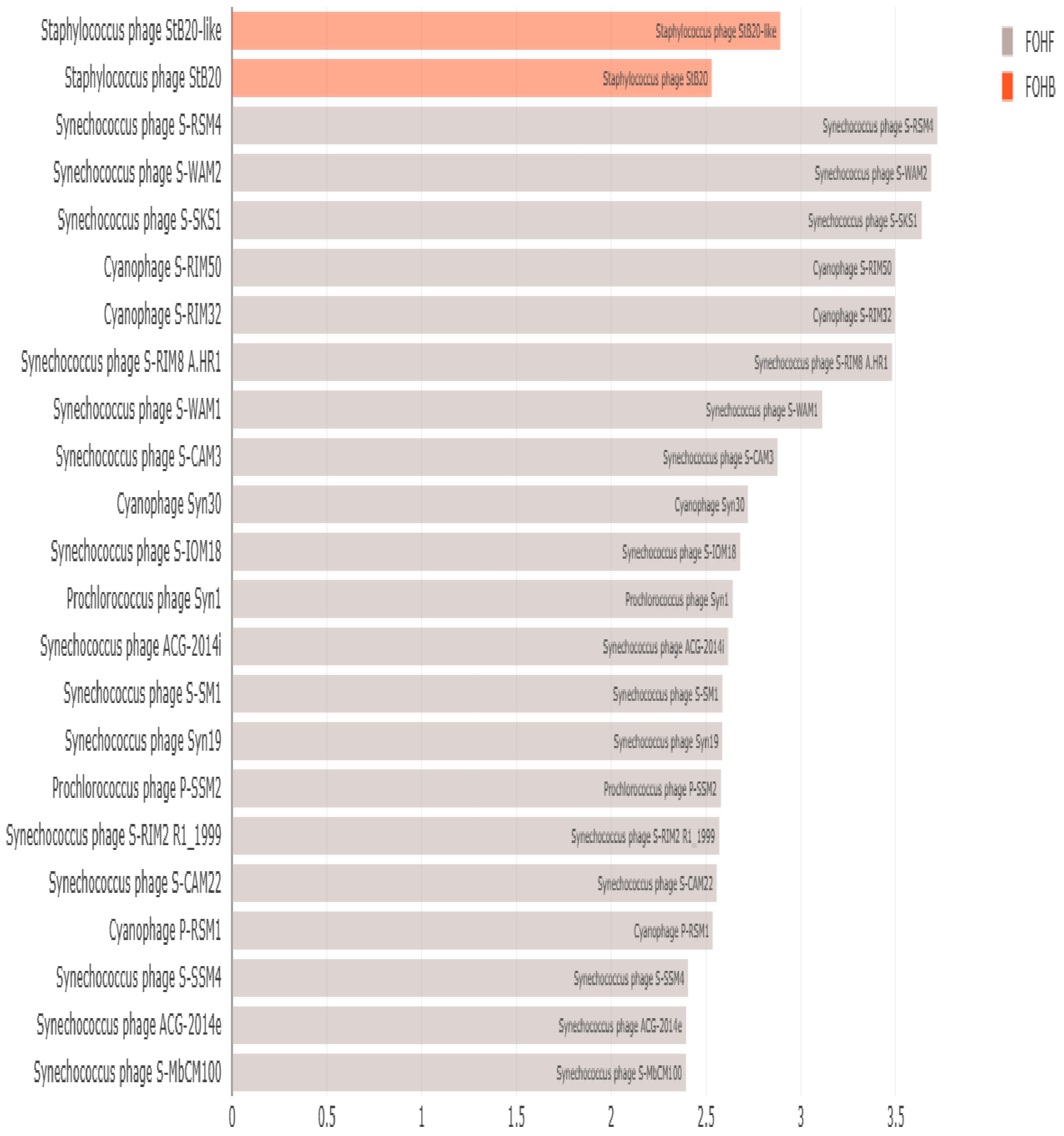

FIG S20: Linear Discriminant Analysis (LDA) effect size (LEfSe) analysis of distinctive phages identified in the FOHB and FOHF samples.

FOHB: Fresh oyster homogenate from bottom cages; FOHF: Fresh oyster homogenate from floating cages.

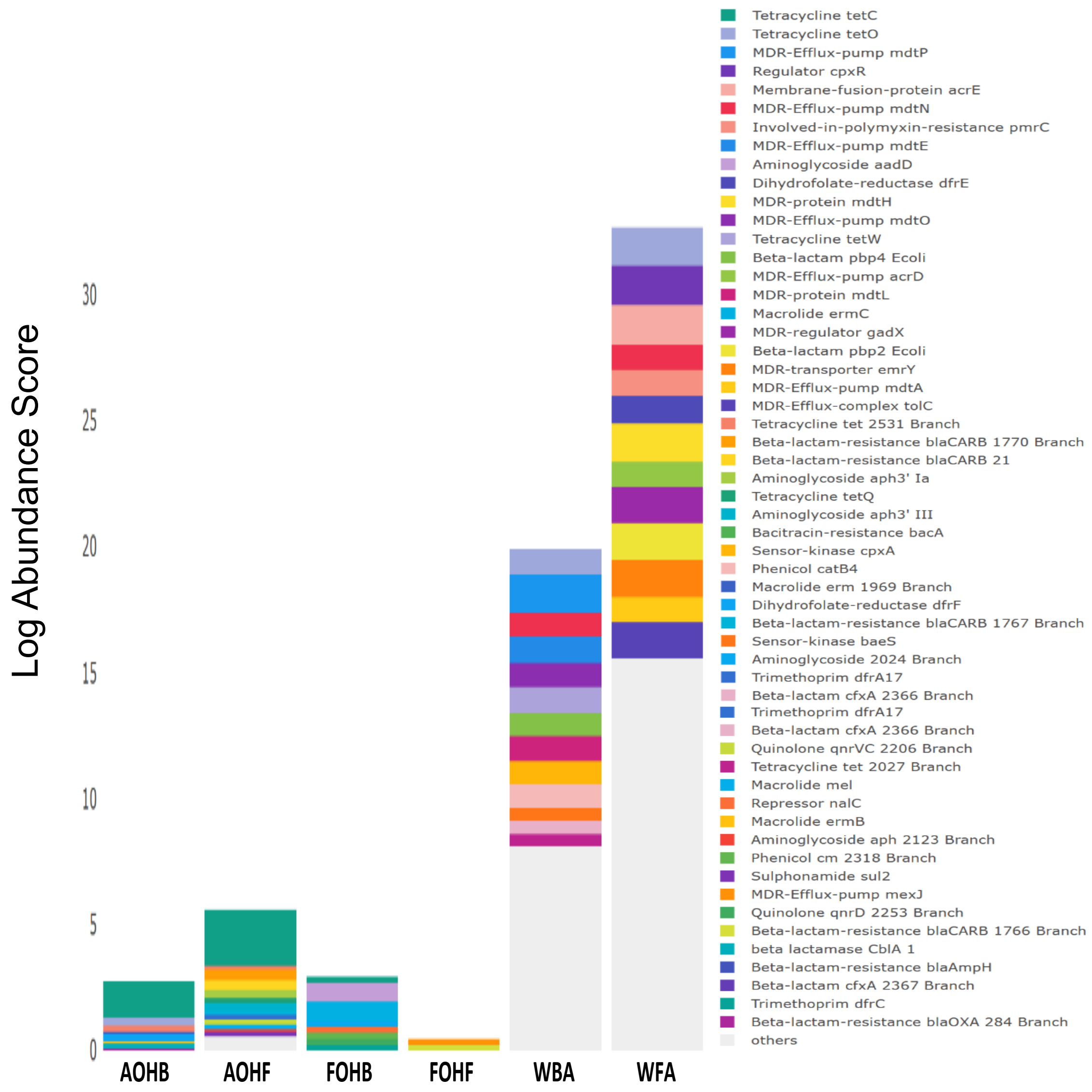

FIG S21: Relative log-abundance of antimicrobial resistance genes per sample type**.**

FOHB: Fresh oyster homogenate from bottom cages; FOHF: Fresh oyster homogenate from floating cages; AOHB: Temperature-abused oyster homogenate from bottom cages; AOHF: Temperature-abused oyster homogenate from floating cages; WBA: Water from the bottom cages area; WFA: Water from the floating cages area.

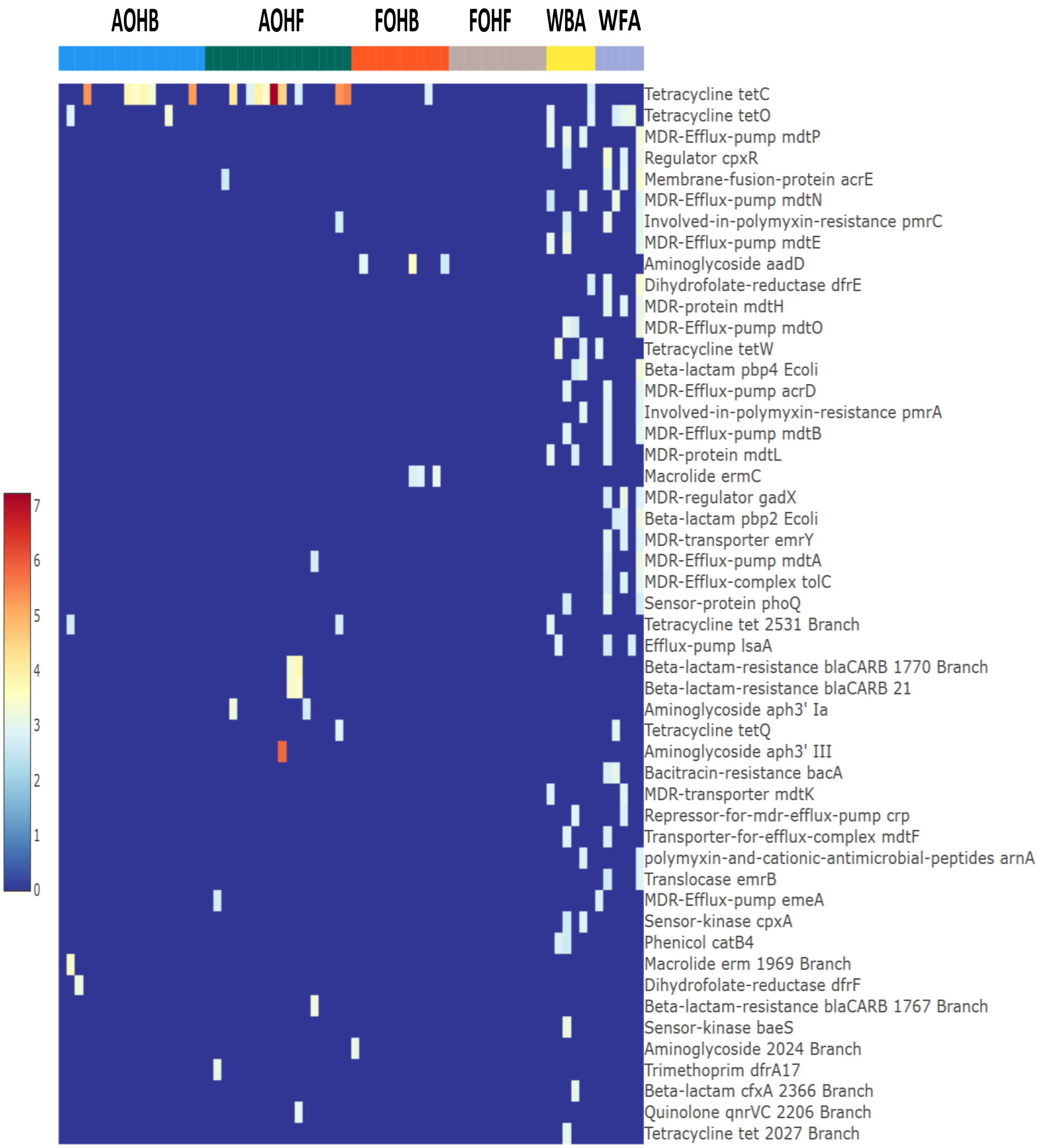

FIG S22: Mean log-abundance of antimicrobial resistance genes across all sample types. FOHB: Fresh oyster homogenate from bottom cages; FOHF: Fresh oyster homogenate from floating cages; AOHB: Temperature-abused oyster homogenate from bottom cages; AOHF: Temperature-abused oyster homogenate from floating cages; WBA: Water from the bottom cages area; WFA: Water from the floating cages area.

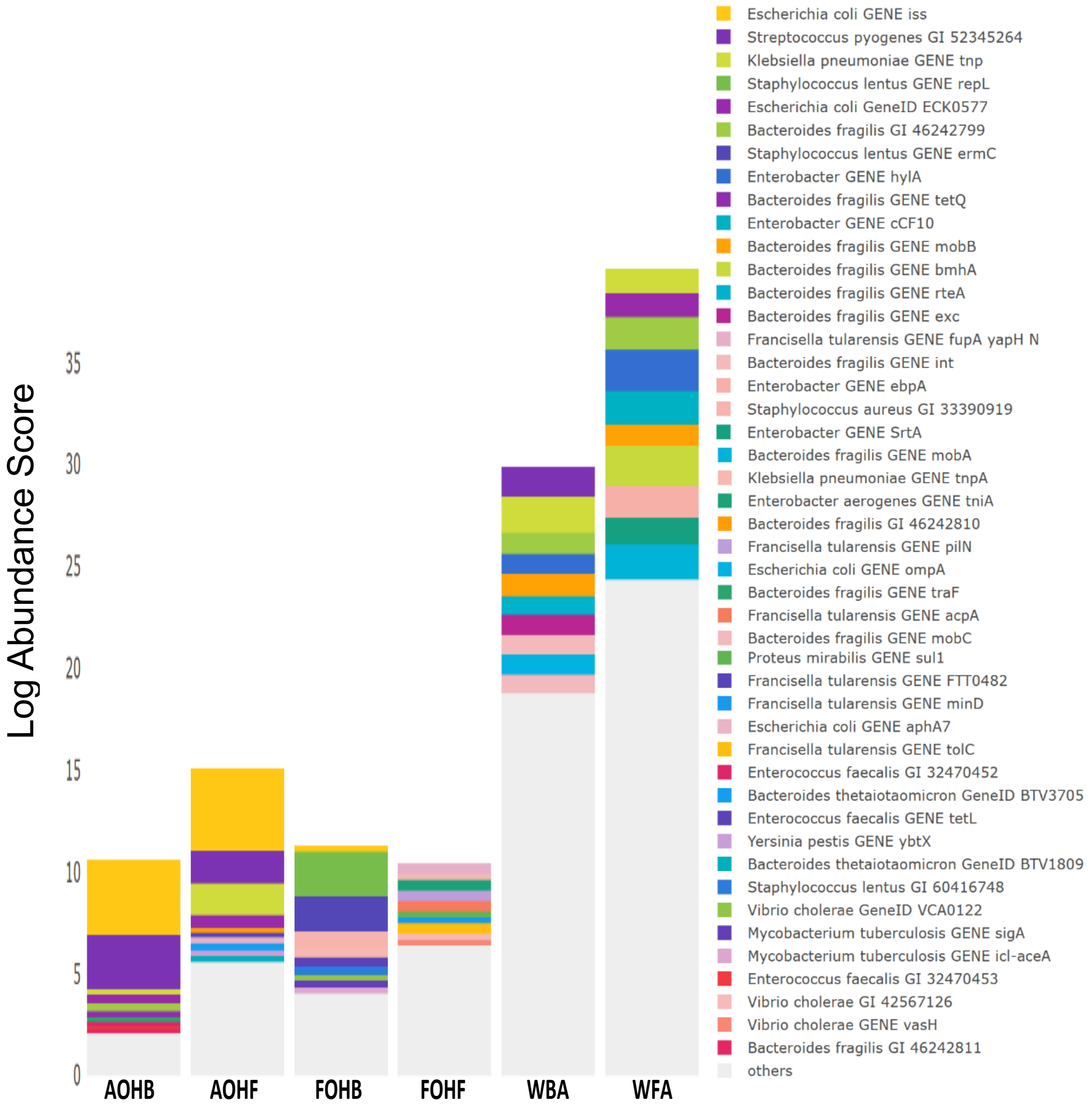

FIG S23: Relative log-abundance of virulence factor genes per sample type.

FOHB: Fresh oyster homogenate from bottom cages; FOHF: Fresh oyster homogenate from floating cages; AOHB: Temperature-abused oyster homogenate from bottom cages; AOHF: Temperature-abused oyster homogenate from floating cages; WBA: Water from the bottom cages area; WFA: Water from the floating cages area.

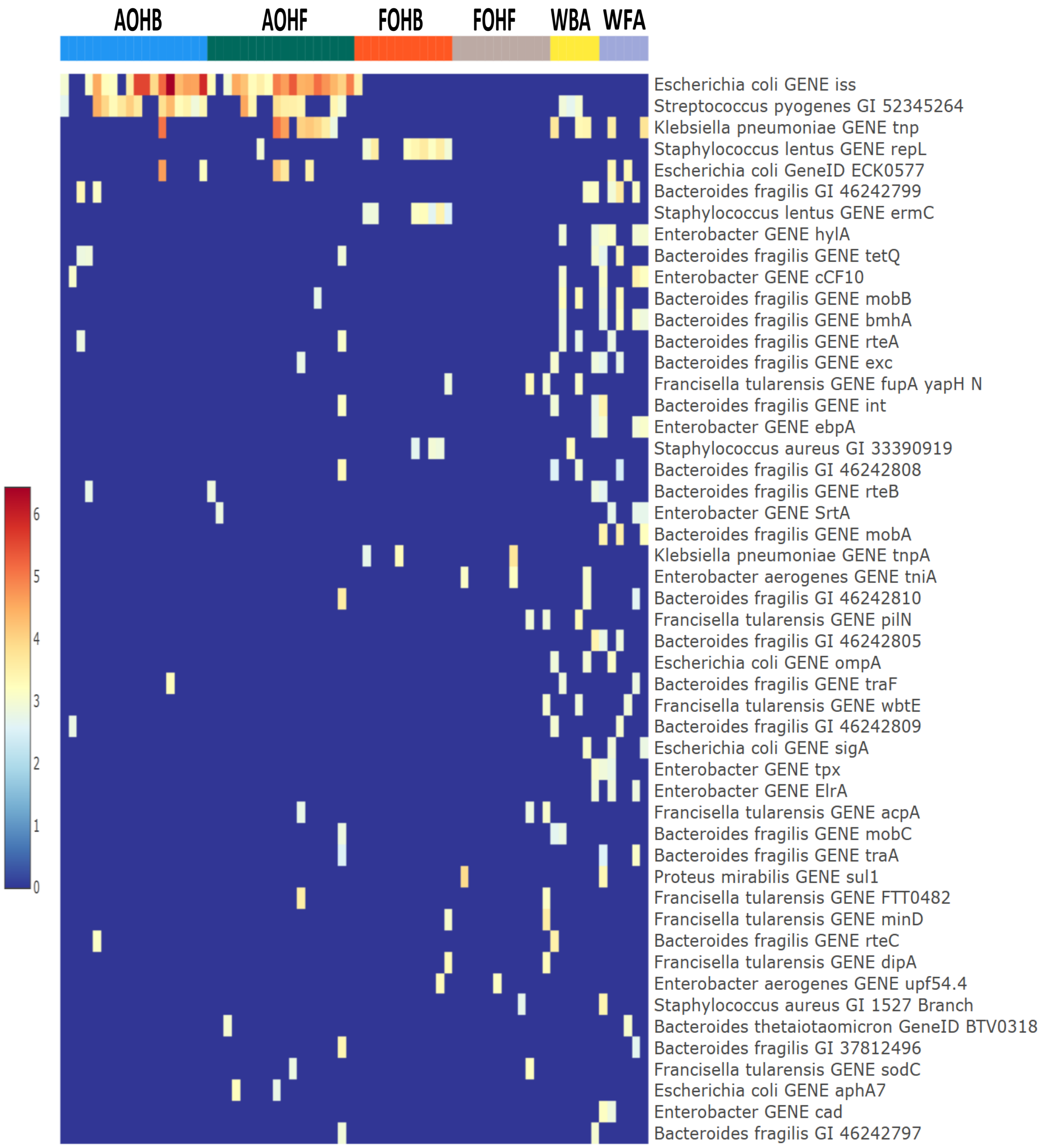

FIG S24: Mean log-abundance of virulence factor genes across all sample types.

FOHB: Fresh oyster homogenate from bottom cages; FOHF: Fresh oyster homogenate from floating cages; AOHB: Temperature-abused oyster homogenate from bottom cages; AOHF: Temperature-abused oyster homogenate from floating cages; WBA: Water from the bottom cages area; WFA: Water from the floating cages area.

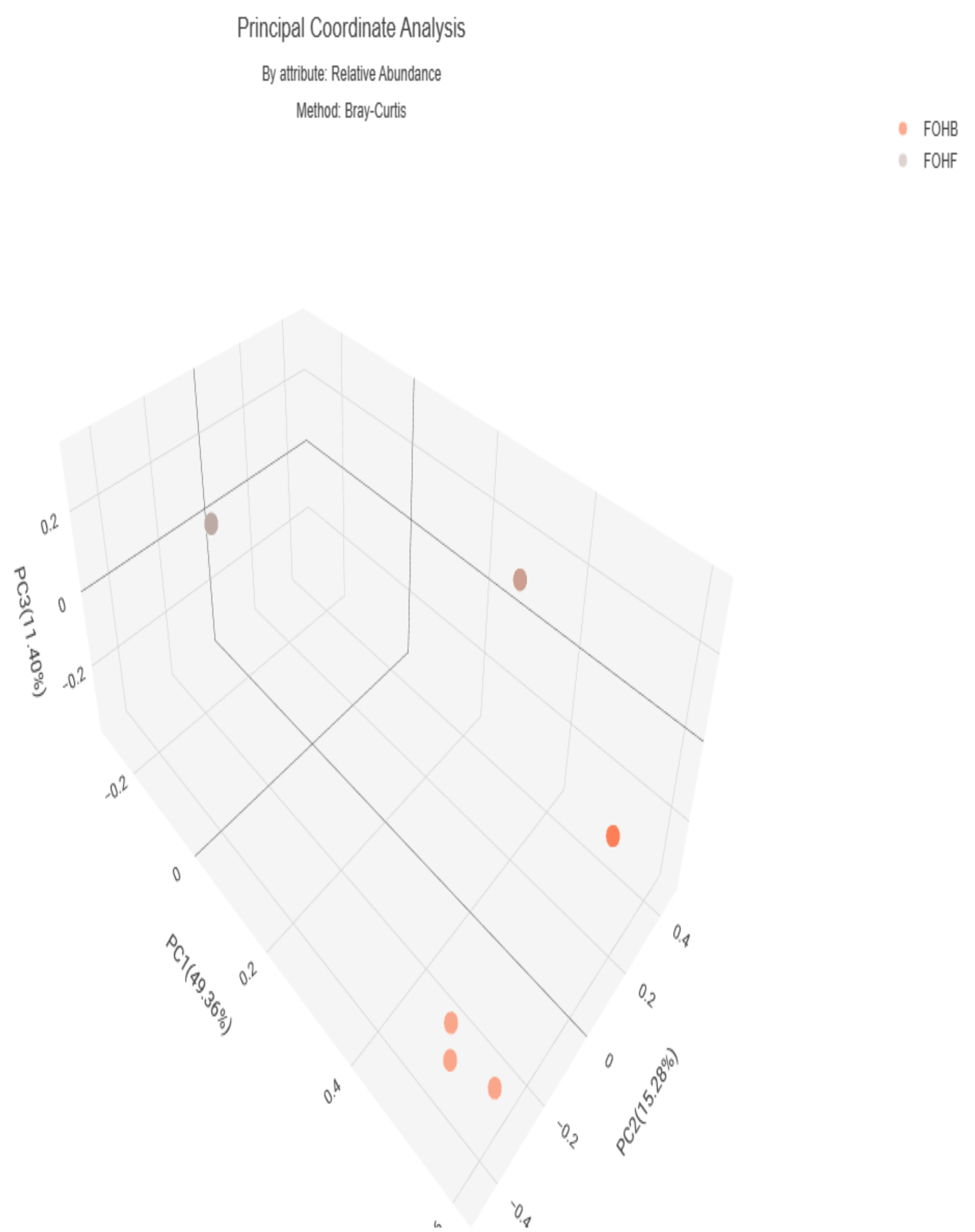

FIG S25: Bray-Curtis index representing the significant differences of antimicrobial resistance genes composition and their relative abundance between FOHB and FOHF.

FOHB: Fresh oyster homogenate from bottom cages; FOHF: Fresh oyster homogenate from floating cages.

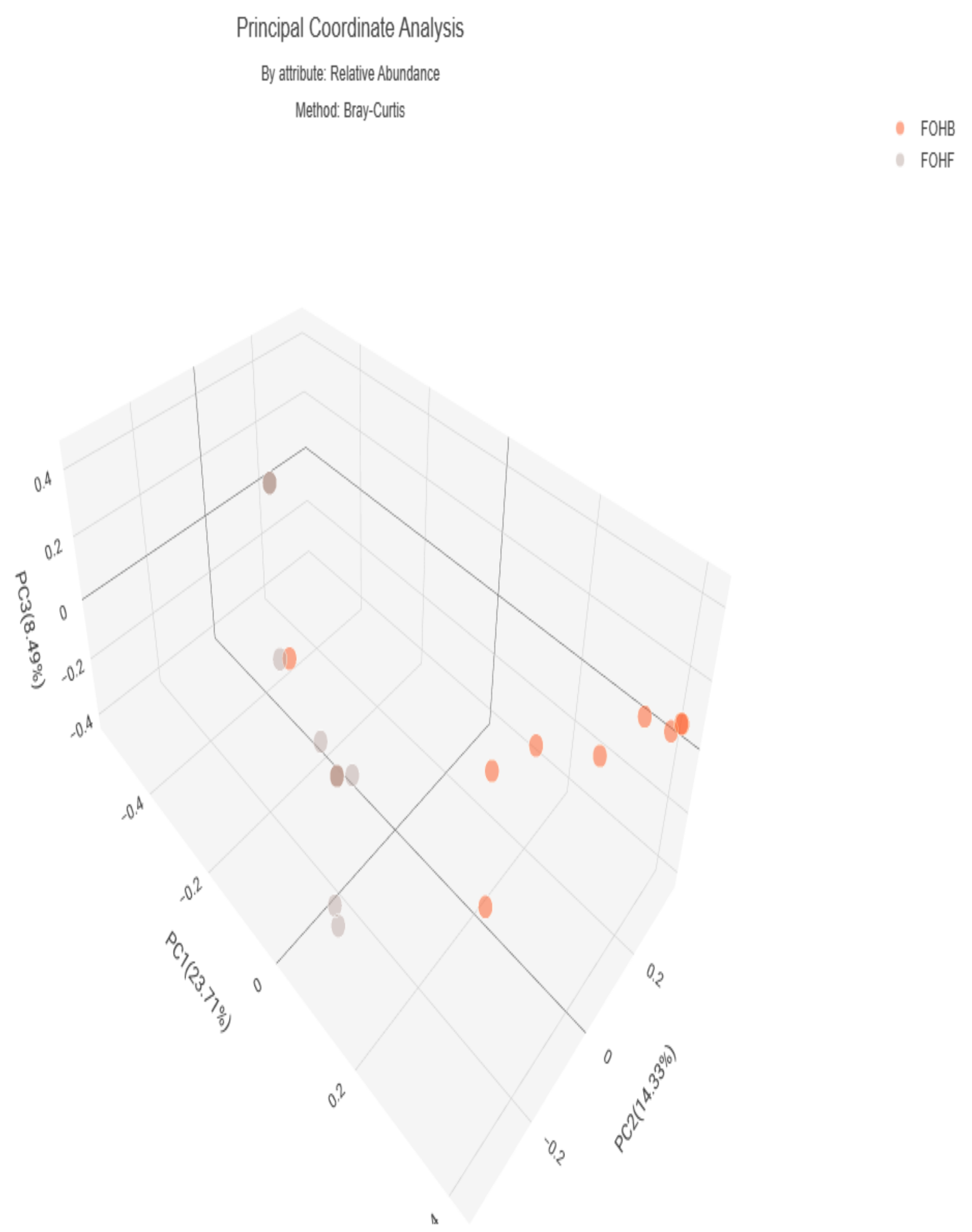

FIG S26: Bray-Curtis index representing the significant differences of virulence factor genes composition and their relative abundance between FOHB and FOHF.

FOHB: Fresh oyster homogenate from bottom cages; FOHF: Fresh oyster homogenate from floating cages.
